# Multitype SIR epidemics among a population partitioned into households with proportionate global mixing

**DOI:** 10.1007/s00285-026-02359-5

**Published:** 2026-04-26

**Authors:** Frank Ball, Liam Critcher

**Affiliations:** 1 School of Mathematical Sciences, University of Nottingham, University Park, Nottingham, NG7 2RD UK; 2 Department of Mathematics, University of Manchester, Alan Turing Building, Oxford Road, Manchester, M13 9PL UK

**Keywords:** Epidemic process, Proportionate global mixing, Final outcome, Branching process, Reproduction numbers, Multivariate central limit theorem, 92D30, 60F05, 60J80

## Abstract

A stochastic model for an SIR epidemic among a population of households that contains *J* types of individuals is considered. Infectives make two kinds of contacts: *local* contacts with individuals in their own household and *global* contacts with individuals from the entire population. Global mixing is *proportionate*. The behaviour of the model as the population size tends to infinity is analysed. An approximating branching process for the early stages of an epidemic is used to determine several different reproduction numbers and the early exponential growth rate. The means of certain final outcome quantities of an epidemic which takes off are determined, together with an associated multivariate central limit theorem. The assumption of proportionate global mixing leads to considerable simplification in both the calculation and proof of asymptotic properties, since key underlying processes are one-dimensional rather than *J*-dimensional.

**Supplementary Information:**

The online version contains supplementary material available at 10.1007/s00285-026-02359-5.

## Introduction

In the last thirty years there has been appreciable work, both theoretical and applied, on models for the spread of epidemics among populations partitioned into small mixing groups, such as households. This structure, which is present in almost all human populations, can have significant impact on both the spread and control of disease. In the *households model*, the population is partitioned into households with different infection rates for within-household and between-household infections; see, for example, Bartoszyński (1972); Becker and Dietz (1995) and Ball et al. (1997). The vast majority of theoretical work on the households model has been concerned with single-type epidemics, thus ignoring factors such as the effects of age, sex and response to vaccine on disease transmission. Becker and Hall (1996) used a multitype branching process approximation to obtain a threshold parameter for the spread of a multitype epidemic in a community of households. Ball and Lyne (2001) studied the spread of a stochastic SIR (susceptible$\rightarrow$infective$\rightarrow$recovered) epidemic amongst a closed, finite population that contains *J* types of individuals and is partitioned into households. By considering a sequence of such epidemics, in which the number of households tends to infinity, they used a coupling argument to show almost sure convergence to a *J*-type branching process, which yields a threshold parameter that determines whether an epidemic with few initial infectives can take off and lead to a major outbreak, and also the (asymptotic) probability of such an outbreak. They also used a heuristic argument to determine the (asymptotic) means of the numbers of each type infected in an epidemic which takes off and proved a multivariate central limit theorem for these and certain other final outcome quantities.

Although the model analysed in Ball and Lyne (2001) is very general, calculation of the probability of a major outbreak and of the mean vector and variance matrix of the above-mentioned multivariate central limit theorem is rather involved, and in particular requires the solution of systems of *J* nonlinear equations. It is well known that the analysis of non-household, multitype epidemic models is greatly simplified under the assumption of *proportionate mixing* (also known as *separable mixing*), i.e. when $\lambda _{ij}=\beta _i \kappa _j$ $(i,j=1,2,\dots , J)$, where $\lambda _{ij}$ is the infection rate between a type-*i* infective and a type-*j* susceptible; see, for example, Andreasen (2003) and Diekmann et al. (2012), Sect. 8.4, for deterministic models, and Becker and Marschner (1990) and Clancy and Pearce (2013), for stochastic models. Under this assumption, the type of an infectious individual does not affect the probability that a given contact is with an individual of a given type, but it does affect the rate at which they make contacts. Models with proportionate mixing arise naturally in several settings. There have been numerous studies in which individuals differ only in their susceptibility, so $\lambda _{ij}=\beta \kappa _j$, for example, Gart (1968), Andersson and Britton (1998) and Katriel (2012). Such differences occur with the leaky vaccine action model (Halloran et al. 1992), under which vaccinated individuals have reduced susceptibility but unchanged infectivity if infected. Another setting is when individuals differ in their activity level, so $\lambda _{ij}=\beta _i \beta _j$, see Britton et al. (2020) who used such a model to show that heterogeneities in activity can decrease appreciably the disease-induced herd immunity level.

In this paper, we show that the analysis of the stochastic multitype households SIR epidemic model is considerably simpler if the *global* (between-household) infection rates follow proportionate mixing. No such constraint is placed upon the *local* (within-household) infection rates, which may depend also on a household’s size and composition of types. Under the assumption of proportionate global mixing, the branching process that approximates the early stages of an epidemic is effectively single-type, leading to considerable simplification in the calculation of reproduction numbers, the probability of a major outbreak and the early exponential growth rate of a major outbreak. The assumption of proportionate global mixing also simplifies the calculation and proof of properties of the final outcome of a major epidemic, since the index set in key processes in the embedding argument is scalar rather than *J*-dimensional. We prove a multivariate central limit theorem for a vector of final state random variables (see Ball and O’Neill (1999)) in the event of a major outbreak. These final state random variables may be quite general and include, for example, the number of individuals of each type infected by the epidemic and the number of households infected (see Sect. 5.3 for further examples).

We also fill, in the present proportionate global mixing setting, a couple of gaps in Ball and Lyne (2001). First, the definition of a major outbreak in Ball and Lyne (2001), called there a global epidemic, is not fully satisfactory in that it is based on the limiting branching process. Here, an epidemic among a population of *m* households is defined to be a major outbreak if it infects at least $\log m$ households. The choice of $\log m$ is just for convenience; $\log m$ can be replaced by any function *f*(*m*) satisfying $f(m) \rightarrow \infty$ and $m^{-1}f(m) \rightarrow 0$ as $m \rightarrow \infty$. Indeed, for any specific parameter values, we can replace $\log m$ by *cm* but the choice of the constant $c>0$ depends on those parameter values. Secondly, the proof of the central limit theorem for the outcome of a major outbreak in Ball and Lyne (2001) ignores the conditioning on a major outbreak when solving the crossing problem for the embedded process, though it yields the correct asymptotic mean and variance. Here, we exploit a lack-of-memory property in the process underlying the embedding, which gives a simple, novel and widely applicable solution to this conditioning problem.

The paper is structured as follows. The stochastic multitype households SIR epidemic model is defined in Sect. 2. Two single-household multitype epidemic models, which play a key role in our analysis of the multitype households model, are described in Sect. 3, together with some associated notation and a related directed random graph. The early stages of an epidemic with few initial infectives is considered in Sect. 4. The approximating branching process is described in Sect. 4.1, with calculation and comparison of reproduction numbers being considered in Sect. 4.2, calculation of the early exponential growth rate outlined briefly in Sect. 4.3 and calculation of the probability of a major epidemic given in Sect. 4.4. The final outcome of a major outbreak is considered in Sect. 5. A heuristic argument yielding the (asymptotic) fraction of individuals of each type that are infected by a major outbreak is given in Sect. 5.1; these *J* fractions, one for each type, are expressed in terms of the solution of a single-variable nonlinear equation. These fractions are related to the extinction probability vector of a backward branching process, which again is effectively single-type, in Sect. 5.2. The above-mentioned central limit theorem is stated in Sect. 5.3. The variance matrix of the central limit theorem is necessarily rather complex; the special case of a highly locally infectious epidemic, in which infection of a member of a household necessarily results in the whole household becoming infected, is considered in Sect. 6, where more explicit results are obtained. In Sect. 7, the special case when all households have size 1 is considered. The model then reduces to a standard multitype SIR epidemic with proportionate mixing and we obtain a special case of the central limit theorem in Ball and Clancy (1993) but with an explicit expression for the elements of the variance matrix. Finally, some brief concluding comments are given in Sect. 8.

The proof of the central limit theorem uses the embedding technique introduced in Scalia-Tomba (1985) (see also Scalia-Tomba (1990) and Ball et al. (1997)) and is similar to that in Ball and Lyne (2001), though simpler as the problem is effectively reduced from *J* dimensions to just one. Nevertheless, the proof is still long and detailed. Thus, the details are given in Supplementary Information, together with a rigorous justification of the approximating branching process and some notes related to calculation of asymptotic properties of the epidemic.

## Model

We consider a multitype SIR model, denoted by $\mathcal {E}$, with household structure in a closed population. We assume that households contain at most $n_{{\text {max}}}$ individuals, where $n_{{\text {max}}}< \infty$. Suppose that there are *J* types of individuals in the population, with types belonging to $\mathcal {J}=\{1,2,\dots ,J\}$. Define the *household category* $\boldsymbol{n}$ as the *J*-tuple containing the number of members of each type in that household. Then, with $\mathbb {Z}_+=\{0,1,\dots \}$, let

$$\begin{aligned} \mathcal {N}=\left\{ \boldsymbol{n}=(n_1,n_2,\dots ,n_J) \in \mathbb {Z}_{+}^{J}:1\le \sum _{j=1}^Jn_j\le n_{{\text {max}}}\right\} \end{aligned}$$

denote the (finite) collection of possible household categories and, for $i \in \mathcal {J}$, let

$$ \mathcal {N}_i= \{\boldsymbol{n}\in \mathcal {N}: n_i >0\} $$

denote the set of household categories that contain at least one type-*i* individual. Suppose that there are $m_{\boldsymbol{n}}$ households of category $\boldsymbol{n}$ ($\boldsymbol{n}\in \mathcal {N}$), with $m=\sum _{\boldsymbol{n}\in \mathcal {N}}m_{\boldsymbol{n}}$ households in total. Writing $\Vert \boldsymbol{n}\Vert =\sum _{i=1}^Jn_i$, the population size (*N*) is then given by

$$\begin{aligned} N=\sum _{\boldsymbol{n}\in \mathcal {N}}\Vert \boldsymbol{n}\Vert m_{\boldsymbol{n}} , \end{aligned}$$

with the number of type-*i* individuals ($N_i$) given by

$$\begin{aligned} N_i=\sum _{\boldsymbol{n}\in \mathcal {N}}n_im_{\boldsymbol{n}} \qquad (i\in \mathcal {J}). \end{aligned}$$

The probability an individual chosen uniformly at random from the population is type *i* is then $\gamma _i=N_i/N$ ($i \in \mathcal {J}$). We assume that $\min _{i \in \mathcal {J}} \gamma _i>0$. The proportion of households of category $\boldsymbol{n}$ is given by $\alpha _{\boldsymbol{n}}=m_{\boldsymbol{n}}/m$ ($\boldsymbol{n}\in \mathcal {N}$). Finally, we denote, by $\alpha _i(\boldsymbol{n})$, the probability an individual chosen uniformly at random among all type-*i* individuals resides in a household of category $\boldsymbol{n}$, which satisfies

$$\begin{aligned} \alpha _i(\boldsymbol{n})=\frac{n_im_{\boldsymbol{n}}}{N_i} \qquad (i \in \mathcal {J}, \boldsymbol{n}\in \mathcal {N}). \end{aligned}$$

The model has infectious dynamics as follows. Individuals begin susceptible (other than a small number of initial infectives which we specify more precisely when necessary) and remain so until they are contacted by an infective, at which point they immediately become infective; there is no latent period in the model. (Most of our results are concerned with the final outcome of $\mathcal {E}$, the distribution of which is invariant to the introduction of a latent period.) For $i \in \mathcal {J}$, a type-*i* individual, upon being infected, remains infectious for a random time $I^{(i)}$, having an arbitrary but specified distribution, with finite mean $\mu _I^{(i)}$. During their infectious period, a type-*i* individual globally contacts any given type-*j* individual according to the points of a Poisson process with rate $\lambda ^G_{ij}/N$ (cf. $\lambda ^G_{ij}/N_j$ in Ball and Lyne (2001)). For $\boldsymbol{n}\in \mathcal {N}$, local (i.e. within-household) contacts occur from a type-*i* individual in a category-$\boldsymbol{n}$ household to any given type-*j* individual *within their household* according to the points of a Poisson process with rate $\lambda ^L_{ij}(\boldsymbol{n})$. (In Sect. 8, we outline how our results can be extended to corresponding models with nonconstant infectivity profiles.) Let ${\Lambda }^G=[\lambda ^G_{ij}]$ and ${\Lambda }^L(\boldsymbol{n})=[\lambda ^L_{ij}(\boldsymbol{n})]$ $(\boldsymbol{n}\in \mathcal {N})$. Individuals recover at the end of their infectious period and play no further role in the epidemic. Finally, the Poisson processes governing infections, as well as the random variables governing infectious period lengths, are all assumed to be mutually independent.

We assume that the global mixing rates follow *proportionate mixing*. i.e. that there exist $\beta _1,\beta _2,\dots ,\beta _J>0$ and $\kappa _1,\kappa _2,\dots ,\kappa _J>0$ such that $\lambda ^G_{ij}=\beta _i\kappa _j$. Further, without loss of generality, we assume that $\sum _{j=1}^J \kappa _j \gamma _j=1$.

## Single-household multitype SIR and related epidemics

We describe here some single-household SIR epidemic models and associated notation that are used in the subsequent analysis.

### Single-household multitype SIR epidemic

For $i \in \mathcal {J}$ and $\boldsymbol{n}\in \mathcal {N}_i$, let $\mathcal {E}_{\boldsymbol{n}, i}(\Lambda ^L(\boldsymbol{n}))$ denote the single-household epidemic model for a category-$\boldsymbol{n}$ household with local infection governed by $\Lambda ^L(\boldsymbol{n})$, infectious periods distributed as in Sect. 2, one initial infective, having type *i*, and all other household members susceptible. For $j \in \mathcal {J}$, let $Y_j$ be the total number of type-*j* individuals infected in $\mathcal {E}_{\boldsymbol{n}, i}(\Lambda ^L(\boldsymbol{n}))$, including the initial infective if $j=i$, and let $A_j$ be the sum of the infectious periods of those $Y_j$ infectives. Let $\mu _{\boldsymbol{n},i,j}(\Lambda ^L(\boldsymbol{n}))=\mathbb {E}[Y_j]$ and note that, by Wald’s identity for multitype SIR epidemics (Ball 1986, Corollary 3.2), $\mathbb {E}[A_j]=\mu _{\boldsymbol{n},i,j}(\Lambda ^L(\boldsymbol{n}))\mu _I^{(j)}$. A formula for $\mu _{\boldsymbol{n},i,j}(\Lambda ^L(\boldsymbol{n}))$ in terms of multivariate Gontcharoff polynomials (Lefèvre and Picard 1990) is given in  (10.1) in the Supplementary Information, Sect. 10.

### Single-household multitype SIR epidemic with outside infection

Consider the following model for an epidemic in a category-$\boldsymbol{n}$ household with outside infection (cf. Addy et al. (1991)), in which local infections are governed by $\Lambda ^L(\boldsymbol{n})$ and infectious periods are distributed as above. Initially, the whole household is susceptible. During the course of the epidemic household members avoid external infection independently, with probability $\pi _i$ for a type-*i* individual. Denote this epidemic by $\tilde{\mathcal {E}}_{\boldsymbol{n}}(\Lambda ^L(\boldsymbol{n}), \boldsymbol{\pi })$, where $\boldsymbol{\pi }=(\pi _1, \pi _2, \dots , \pi _J)$. For $j \in \mathcal {J}$, let $\tilde{Y}_j$ be the number of type-*j* individuals infected in $\tilde{\mathcal {E}}(\Lambda ^L(\boldsymbol{n}), \boldsymbol{\pi })$ and let $\tilde{A}_j$ be the sum of the infectious periods of those $\tilde{Y}_j$ infectives. Let $\tilde{\mu }_{\boldsymbol{n},j}(\Lambda ^L(\boldsymbol{n}),\boldsymbol{\pi })=\mathbb {E}[\tilde{Y}_j]$ and note that $\mathbb {E}[\tilde{A}_j]=\tilde{\mu }_{\boldsymbol{n},j}(\Lambda ^L(\boldsymbol{n}),\boldsymbol{\pi })\mu _I^{(j)}$. A formula for $\tilde{\mu }_{\boldsymbol{n},j}(\Lambda ^L(\boldsymbol{n}),\boldsymbol{\pi })$ in terms of multivariate Gontcharoff polynomials is given in (10.2) in the Supplementary Information, Sect. 10.

### Associated random directed graph

Let $\mathcal {G}_{\boldsymbol{n}}(\Lambda ^L(\boldsymbol{n}))$ be the following directed random graph associated with an epidemic among a category-$\boldsymbol{n}$ household; cf., for example, the *locally dependent random graph* in Kuulasmaa (1982) and the *epidemic percolation network* outlined in Kenah and Robins (2007) and Kenah and Miller (2011). The vertices in $\mathcal {G}_{\boldsymbol{n}}(\Lambda ^L(\boldsymbol{n}))$ are the individuals in the household. For each individual we draw up a list of who they would attempt to infect if they were to become infected, by sampling first from their infectious period and then from their Poisson processes governing local infection. For any two distinct individuals, *k*, *l* say, in the household, there is a directed edge from *k* to *l* in $\mathcal {G}_{\boldsymbol{n}}(\Lambda ^L(\boldsymbol{n}))$ if and only if *l* is in *k*’s list. For the epidemic $\mathcal {E}_{\boldsymbol{n}, i}(\Lambda ^L(\boldsymbol{n}))$, the random variable $Y_j$ is given by the number of type-*j* individuals in the household that in $\mathcal {G}_{\boldsymbol{n}}(\Lambda ^L(\boldsymbol{n}))$ have a chain of directed edges leading to them from the initial infective. If $\mathcal {G}_{\boldsymbol{n}}(\Lambda ^L(\boldsymbol{n}))$ is augmented by the infectious period of each individual in the household then the random variable $A_j$ is available. If $\mathcal {G}_{\boldsymbol{n}}(\Lambda ^L(\boldsymbol{n}))$ is augmented so that, for each directed edge, the time of the contact relative to when the infector was infected is recorded, then the entire time course of $\mathcal {E}_{\boldsymbol{n}, i}(\Lambda ^L(\boldsymbol{n}))$ can be reconstructed. Similarly, for the epidemic $\tilde{\mathcal {E}}_{\boldsymbol{n}}(\Lambda ^L(\boldsymbol{n}), \boldsymbol{\pi })$, once the set of individuals who are infected externally is determined, the random variable $\tilde{Y}_j$ is available from $\mathcal {G}_{\boldsymbol{n}}(\Lambda ^L(\boldsymbol{n}))$, as is the random variable $\tilde{A}_j$ if $\mathcal {G}_{\boldsymbol{n}}(\Lambda ^L(\boldsymbol{n}))$ is augmented by the infectious periods.

## Early phase of epidemic

### Approximating branching process

For $i,j \in \mathcal {J}$, a typical type-*i* infective makes global contacts with type-*j* individuals at rate $\beta _i \kappa _j N_j/N=\beta _i \kappa _j \gamma _j$. Thus, by standard properties of Poisson processes, a typical type-*i* infective makes global contacts at overall rate $\sum _{j=1}^J \beta _i \kappa _j \gamma _j=\beta _i$, since $\sum _{j=1}^J\gamma _j \kappa _j=1$, and each such global contact is with a type-*j* individual independently with probability $P_j=\beta _i \kappa _j \gamma _j/\left( \sum _{k=1}^J \beta _i \kappa _k \gamma _k\right) =\kappa _j \gamma _j$. Note that $P_j$ is independent of the type of the infector. During the early phase of an epidemic with few initial infectives in a large population, each global contact is highly likely to be with an individual who resides in a completely susceptible household. It follows that the early phase of the epidemic $\mathcal {E}$ in a large population can be approximated by the following branching process, denoted by $\mathcal {B}$.

Individuals in $\mathcal {B}$ correspond to single-household epidemics. Consider the epidemic $\mathcal {E}_{\boldsymbol{n}, i}(\Lambda ^L(\boldsymbol{n}))$ defined in Sect. 3.1 and suppose that infectives in that epidemic make global contacts at the points of independent Poisson processes, having rate $\beta _k$ for a type-*k* infective. Denote the epidemic $\mathcal {E}_{\boldsymbol{n}, i}(\Lambda ^L(\boldsymbol{n}))$ augmented with such global contacts by $\hat{\mathcal {E}}_{\boldsymbol{n}, i}(\Lambda ^L(\boldsymbol{n}), \boldsymbol{\beta })$, where $\boldsymbol{\beta }=(\beta _1,\beta _2,\dots , \beta _J)$. For $j \in \mathcal {J}$, each global contact is with a type-*j* individual independently with probability $P_j$. Further, for $j \in \mathcal {J}$ and $\boldsymbol{n}' \in \mathcal {N}$, each global contact with a type-*j* individual is with an individual who resides in a category-$\boldsymbol{n}'$ household independently with probability $\alpha _j(\boldsymbol{n}')$. Suppose that such a global contact is with a type-*j* individual residing in a category-$\boldsymbol{n}'$ household. Then a new individual is born in $\mathcal {B}$, whose offspring is determined in the obvious fashion from global contacts emanating from an independent realisation of $\hat{\mathcal {E}}_{\boldsymbol{n}', j}(\Lambda ^L(\boldsymbol{n}'), \boldsymbol{\beta })$. We say that such an individual has type $(j, \boldsymbol{n}')$ in $\mathcal {B}$. This typing is useful when describing the offspring law of $\mathcal {B}$ but note that $\mathcal {B}$ is effectively single-type, since the types, $(i, \boldsymbol{n})$, of individuals in $\mathcal {B}$ are independently and identically distributed. Note also that, depending on how the initial infective(s) are chosen, the ancestor(s) in $\mathcal {B}$ may follow a different law to all subsequent individuals.

The approximation of $\mathcal {E}$ by $\mathcal {B}$ is made rigorous in the Supplementary Information, Sect. 9.1, by considering a sequence of epidemics $\mathcal {E}^{(v)}$ $(v=1,2,\dots )$ in which the number of households $m^{(v)}\rightarrow \infty$ as $v \rightarrow \infty$ and using a coupling argument; see the proof of Theorem 9.1 and Remark 9.2. For $v=1,2,\dots$, the epidemic $\mathcal {E}^{(v)}$ occurs among a population comprising $m_{\boldsymbol{n}}^{(v)}$ households of category $\boldsymbol{n}$ $(\boldsymbol{n}\in \mathcal {N})$. It is assumed, for each $\boldsymbol{n}\in \mathcal {N}$, that $\alpha _{\boldsymbol{n}}^{(v)}=m_{\boldsymbol{n}}^{(v)}/ m^{(v)} \rightarrow \alpha _{\boldsymbol{n}}$ as $v \rightarrow \infty$, and that the configuration of initial infectives (i.e. their number and distribution among the household categories) is the same for all *v*. For simplicity, we assume that $n_{{\text {max}}}$ (and hence also $\mathcal {N}$) is independent of *v*. However, this can be relaxed, as in Ball and Lyne (2001), where

$$\begin{aligned} \mathcal {N}=\left\{ \boldsymbol{n}=(n_1,n_2,\dots ,n_J) \in \mathbb {Z}_{+}^{J}:\sum _{j=1}^Jn_j\ge 1 \right\} , \end{aligned}$$

so there is no maximal household size in the limit $v \rightarrow \infty$, but then the proofs are more complicated and further conditions are required on the convergence of $\alpha _{\boldsymbol{n}}^{(v)}$ to $\alpha _{\boldsymbol{n}}$ ($\boldsymbol{n}\in \mathcal {N}$).

### Reproduction numbers

Several reproduction numbers have been defined for single-type households models, see, for example, Becker and Dietz (1995); Goldstein et al. (2009) and Ball et al. (2016). These have natural extensions to multitype households models, though their calculation is often difficult and apart from $R_*$ (defined in Sect. 4.2.1 below) few have been considered in the literature. Indeed, the only example of which we are aware is Pellis et al. (2020), in which calculation of $R_0$ for a general 2-type households model is described. We consider the main reproduction numbers, apart from the exponential-growth associated reproduction number $R_r$ (see Goldstein et al. (2009)), and show that their calculation is generally straightforward under the assumption of proportionate global mixing. We also prove a comparison theorem for these reproduction numbers, which yields bounds for the critical vaccination coverage (Remark 4.2).

#### Households-based reproduction number $R_*$.

Let $R_*$ be the mean number of offspring of an individual in $\mathcal {B}$ who is not an ancestor. Such an individual has type $(i, \boldsymbol{n})$ with probability $P_i \alpha _i(\boldsymbol{n})=\kappa _i\gamma _i \alpha _i(\boldsymbol{n})$. The number of global contacts emanating from $\hat{\mathcal {E}}_{\boldsymbol{n}, i}(\Lambda ^L(\boldsymbol{n}), \boldsymbol{\beta })$ follows a mixed-Poisson distribution having random mean distributed as $\sum _{j=1}^J \beta _j A_j$, where $A_j$, defined in Sect. 3.1, has mean $\mu _{\boldsymbol{n},i,j}(\Lambda ^L(\boldsymbol{n}))\mu _I^{(j)}$. Hence,

4.1 $$\begin{aligned} R_*= \sum _{i=1}^J\kappa _i\gamma _i\sum _{\boldsymbol{n}\in \mathcal {N}_i}\alpha _i(\boldsymbol{n})\sum _{j=1}^J\mu _{\boldsymbol{n},i,j}(\Lambda ^L(\boldsymbol{n}))\mu _I^{(j)}\beta _j. \end{aligned}$$

The reproduction number $R_*$ serves as a threshold parameter for the epidemic $\mathcal {E}$; in the limit as $m \rightarrow \infty$ a major outbreak occurs with non-zero probability if and only if $R_*>1$. Moreover, the limiting probability of a major outbreak is given by the probability that $\mathcal {B}$ does not go extinct. This is made precise and justified rigorously in the Supplementary Information, Sect. 9.1, by considering the above-mentioned sequence of epidemics $\mathcal {E}^{(v)}$ $(v=1,2,\dots )$; see Theorem 9.1, in which a major outbreak is defined as one which infects at least $\log m^{(v)}$ households and it is shown that, as $v \rightarrow \infty$, the probability of a major outbreak converges to the non-extinction probability of $\mathcal {B}$. Moreover, in the definition of a major outbreak, $\log m$ can be replaced by any function $f:\mathbb {N} \rightarrow \mathbb {R}_+$ satisfying $f(m) \rightarrow \infty$ and $m^{-1}f(m) \rightarrow 0$ as $m \rightarrow \infty$; see Remark 9.5 at the end of Sect. 9.2.2 in the Supplementary Information.

#### Individual-based reproduction number $R_I$.

The reproduction number $R_*$ is relatively easy to calculate and is useful for determining whether or not a major outbreak is possible. However, it can be misleading when making comparisons between epidemics among populations with different household structures; see, for example, Ball et al. (2010), Figure 7. Thus several individual-based reproduction numbers have been proposed for households models. The individual-based reproduction number $R_I$, introduced in a single-type households setting by Becker and Dietz (1995), attributes all secondary cases in a household to the primary case in that household. In the general multitype household model this leads to an approximating branching process, $\mathcal {B}_I$ say, with 2*J* types, corresponding to primary and secondary cases of the *J* types, and $R_I$ is the maximal eigenvalue of the $2J \times 2J$ offspring mean matrix of $\mathcal {B}_I$; see Ball et al. (1997), Sect. 3.6, for the case $J=1$. However, under proportionate global mixing, the type space of $\mathcal {B}_I$ can be reduced to $J+1$ types: primary cases, whose type $(j, \boldsymbol{n}')$ in $\mathcal {B}$ is distributed as described in Sect. 4.1, and secondary cases, typed $1,2,\dots , J$ in the obvious fashion. The reproduction number $R_I$ is the maximal eigenvalue of the $(J+1) \times (J+1)$ offspring mean matrix $\boldsymbol{M}_I$ of $\mathcal {B}_I$.

Note that in $\mathcal {B}_I$, primary cases beget both primary and secondary cases but secondary cases beget only primary cases, so $\boldsymbol{M}_I$ takes the partitioned form

$$ \boldsymbol{M}_I= \begin{pmatrix} a & \boldsymbol{u}^{\top } \\ \boldsymbol{v}& \boldsymbol{0}_{J\times J} \end{pmatrix}, $$

where $\top$ denotes transpose. Here, $\boldsymbol{v}=(v_1, v_2, \dots , v_J)^{\top }$, where $v_k=\beta _k\mu _I^{(k)}$ is the mean number of global contacts made by a type-*k* infective. The scalar *a* is the mean number of global contacts made by a typical primary case; such an individual is type *i* with probability $P_i=\kappa _i \gamma _i$, so

$$ a=\sum _{i=1}^J \kappa _i \gamma _i\beta _i \mu _I^{(i)}. $$

Finally, $\boldsymbol{u}^{\top }=(u_1,u_2, \dots , u_J)$, where $u_j$ is the mean number of type-*j* secondary cases created by a typical primary case. Such a primary case has type $(i, \boldsymbol{n})$ in $\mathcal {B}$ with probability $\kappa _i\gamma _i \alpha _i(\boldsymbol{n})$, and all type-*j* secondary cases in the subsequent single-household epidemic are attributed to that primary case, so

$$ u_j=\sum _{i=1}^J \kappa _i\gamma _i \sum _{\boldsymbol{n}\in \mathcal {N}_i} \alpha _i(\boldsymbol{n})[\mu _{\boldsymbol{n},i,j}(\Lambda ^L(\boldsymbol{n}))-\delta _{ij}]\qquad (j \in \mathcal {J}), $$

where $\delta _{ij}$ denotes the Kronecker delta with $\delta _{ij}=1$ if $i=j$ and $\delta _{ij}=0$ otherwise.

Observe that $\boldsymbol{M}_I=\boldsymbol{A}\boldsymbol{B}$, where

4.2 $$\begin{aligned} \boldsymbol{A}=\begin{pmatrix} a & v_1 & \dots & v_J\\ 0 & -v_1 & \dots & -v_J \end{pmatrix}^{\top } \qquad {\text {and}}\qquad \boldsymbol{B}=\begin{pmatrix} 1 & \frac{u_1}{a} & \dots & \frac{u_J}{a}\\ 0 & \frac{u_1}{a} & \dots & \frac{u_J}{a} \end{pmatrix}. \end{aligned}$$

The non-zero eigenvalues of $\boldsymbol{A}\boldsymbol{B}$ and $\boldsymbol{B}\boldsymbol{A}$ are identical (see for example Mardia et al. (1979), page 468), so $R_I$ is given by the maximal eigenvalue of the $2 \times 2$ matrix $\boldsymbol{B}\boldsymbol{A}$ and a simple calculation yields

$$ R_I=\frac{a+\sqrt{a^2+4 \boldsymbol{u}^{\top }\boldsymbol{v}}}{2}. $$

#### Basic reproduction number $R_0$.

The individual-based reproduction number $R_I$ is not fully satisfactory as normally some secondary cases in a household are infected by other secondary cases, and not by the primary case. Pellis et al. (2012) extended the definition of the basic reproduction number $R_0$ to models with small mixing groups, such as households, by viewing the epidemic on a generation basis. Consider a realisation of the epidemic $\mathcal {E}_{\boldsymbol{n}, i}(\Lambda ^L(\boldsymbol{n}))$ obtained using the random directed graph $\mathcal {G}_{\boldsymbol{n}}(\Lambda ^L(\boldsymbol{n}))$, as described in Sect. 3.3. For that epidemic, the initial (type-*i*) infective belongs to generation 0 and the generation of any secondary case is given by the number of edges in the shortest path from the initial infective to that individual in $\mathcal {G}_{\boldsymbol{n}}(\Lambda ^L(\boldsymbol{n}))$. Thus, generation 1 comprises all individuals that are contacted by the initial infective, generation 2 comprises all individuals that are contacted by generation-1 individuals but are not contacted by the initial infective, and so on.

Consider a typical type-$(i, \boldsymbol{n})$ individual, $i_*$ say, in the approximating branching process $\mathcal {B}$. For $k=0,1,\dots$, all global contacts that emanate from generation-*k* individuals in the epidemic $\mathcal {E}_{\boldsymbol{n}, i}(\Lambda ^L(\boldsymbol{n}))$ are deemed to have occurred when $i_*$ has age $k+1$. Then $R_0$ is given by the asymptotic (Malthusian) geometric growth rate of $\mathcal {B}$; see Ball et al. (2016) for further details in the single-type setting. Note that this definition of $R_0$ coincides with the usual definition (see, for example, Heesterbeek and Dietz (1996)) when all households have size 1. For $i, j \in \mathcal {J}$, $\boldsymbol{n}\in \mathcal {N}$ and $k=0,1,\dots$, let $\mu _{\boldsymbol{n},i,j}^{(k)}(\Lambda ^L(\boldsymbol{n}))$ be the mean number of type-*j* individuals in generation *k* of the epidemic $\mathcal {E}_{\boldsymbol{n}, i}(\Lambda ^L(\boldsymbol{n}))$. For $k=1,2,\dots$, let $\nu _k$ be the mean number of offspring a typical individual in $\mathcal {B}$ has at age *k*. Then $\nu _1=a$, defined in Sect. 4.2.2, and recalling that a typical individual in $\mathcal {B}$ has type $(i, \boldsymbol{n})$ with probability $\kappa _i\gamma _i \alpha _i(\boldsymbol{n})$,

4.3 $$\begin{aligned} \nu _k=\sum _{i=1}^J \kappa _i \gamma _i \sum _{\boldsymbol{n}\in \mathcal {N}_i} \alpha _i(\boldsymbol{n}) \sum _{j=1}^J \mu _{\boldsymbol{n},i,j}^{(k-1)}(\Lambda ^L(\boldsymbol{n}))\mu _I^{(j)} \beta _j\qquad (k=2,3,\dots ). \end{aligned}$$

It follows that $R_0$ is given by the unique positive solution of the discrete-time Lotka-Euler equation $g_0(\lambda )=0$, where

4.4 $$\begin{aligned} g_0(\lambda )=1-\sum _{k=1}^{\infty } \frac{\nu _k}{\lambda ^{k}}. \end{aligned}$$

The means $\mu _{\boldsymbol{n},i,j}^{(k)}(\Lambda ^L(\boldsymbol{n}))$ $(i, j \in \mathcal {J}, \boldsymbol{n}\in \mathcal {N}_i, k=0,1,\dots )$ can be computed by extending the recursive method in Pellis et al. (2012), Appendix A, to multitype epidemics, though the details are complicated. Consider the special case where $I^{(i)}{\mathop {=}\limits ^{D}}I^{(j)}$ for all $i, j \in \mathcal {J}$, where ${\mathop {=}\limits ^{D}}$ denotes equal in distribution, and for each $\boldsymbol{n}\in \mathcal {N}$ with $\Vert \boldsymbol{n}\Vert \ge 2$, $\lambda ^L_{ij}(\boldsymbol{n}) =\lambda ^L(\boldsymbol{n})$ $(i,j \in \mathcal {J})$. Thus local infection is homogeneously mixing, with rate that may depend on household category. Then, for $k \ge 1$, everyone in a household apart from the initial infective is equally likely to be a generation-*k* case, so

$$ \mu _{\boldsymbol{n},i,j}^{(k)}(\Lambda ^L(\boldsymbol{n}))=\frac{n_i-\delta _{ij}}{\Vert \boldsymbol{n}\Vert -1}\mu _{\boldsymbol{n}}^{(k)}(\lambda ^L(\boldsymbol{n})) \qquad (i,j \in \mathcal {J}, \boldsymbol{n}\in \mathcal {N}_i, \Vert \boldsymbol{n}\Vert \ge 2, k=1,2,\dots ), $$

where $\mu _{\boldsymbol{n}}^{(k)}(\lambda ^L(\boldsymbol{n}))$ is the mean size of generation *k* in a single-household epidemic in a category-$\boldsymbol{n}$ household. Thus in this case $\mu _{\boldsymbol{n},i,j}^{(k)}(\Lambda ^L(\boldsymbol{n}))$ is computed easily using Pellis et al. (2012), Appendix A.

#### Perfect vaccine-associated reproduction number $R_V$.

Suppose that $R_*>1$ and a fraction *p* of the population, chosen uniformly at random, is vaccinated with a vaccine that guarantees immunity. Let $R_*(p)$ be the post-vaccination value of $R_*$ and $p_C$ be the unique solution in (0, 1) of $R_*(p)=1$. Then $R_V=(1-p_C)^{-1}$, see Goldstein et al. (2009), who introduced this reproduction number for a single-type households model. The rationale underlying this definition of $R_V$ is that the critical vaccination coverage $p_C=1-R_V^{-1}$, paralleling the formula $1-R_0^{-1}$ for a homogeneously mixing epidemic model.

#### Comparison of reproduction numbers.

Following Ball et al. (2016), we call an epidemic growing if $R_*>1$ and declining if $R_*<1$.

##### Theorem 4.1

- $$R_*=1 \iff R_I=1 \iff R_0=1$$.
- In a growing epidemic,
  $$ R_*\ge R_I \ge R_V \ge R_0 >1. $$
  In a declining epidemic
  $$ R_* \le R_I \le R_0<1. $$

##### Proof

The proof parallels that of Ball et al. (2016), Theorem 1. We give the details for the comparisons involving $R_*, R_I$ and $R_0$. The proof of $R_I \ge R_V \ge R_0$ for a growing epidemic is lengthy and left as an exercise.

The characteristic polynomial of $\boldsymbol{B}\boldsymbol{A}$, see (4.2), is $x^2-ax -\boldsymbol{u}^{\top }\boldsymbol{v}$, so $R_I$ is given by the unique positive solution of $g_I(\lambda )=0$, where

4.5 $$\begin{aligned} g_I(\lambda )=1-\frac{a}{\lambda }-\frac{\boldsymbol{u}^{\top }\boldsymbol{v}}{\lambda ^2}. \end{aligned}$$

Now

4.6 $$\begin{aligned} \boldsymbol{u}^{\top }\boldsymbol{v}=\sum _{j=1}^J u_j v_j&= \sum _{j=1}^J\left( \sum _{i=1}^J \kappa _i\gamma _i \sum _{\boldsymbol{n}\in \mathcal {N}_i} \alpha _i(\boldsymbol{n})[\mu _{\boldsymbol{n},i,j}(\Lambda ^L(\boldsymbol{n}))-\delta _{ij}]\right) \beta _j \mu _I^{(j)}\\&=\sum _{j=1}^J\sum _{i=1}^J \kappa _i\gamma _i \sum _{\boldsymbol{n}\in \mathcal {N}_i} \alpha _i(\boldsymbol{n})\mu _{\boldsymbol{n},i,j}(\Lambda ^L(\boldsymbol{n})) \beta _j \mu _I^{(j)}-\sum _{j=1}^J \kappa _j\gamma _j \beta _j \mu _I^{(j)}\nonumber \\&=R_*-a,\nonumber \end{aligned}$$

so $R_*$ is given by the unique positive solution of $g_*(\lambda )=0$, where

4.7 $$\begin{aligned} g_*(\lambda )= 1-\frac{a+\boldsymbol{u}^{\top }\boldsymbol{v}}{\lambda }. \end{aligned}$$

Further, recalling that $\nu _1=a$ and noting that $\sum _{k=2}^{\infty } \mu _{\boldsymbol{n},i,j}^{(k-1)}(\Lambda ^L(\boldsymbol{n}))=\mu _{\boldsymbol{n},i,j}(\Lambda ^L(\boldsymbol{n}))-\delta _{ij}$ $(i,j \in \mathcal {J})$, we have, using (4.4), that

4.8 $$\begin{aligned} g_0(\lambda )=1-\frac{a}{\lambda }-\sum _{k=2}^{\infty } \frac{\nu _k}{\lambda ^{k}} \end{aligned}$$

and, using (4.3) and (4.6), that $\sum _{k=2}^{\infty } \nu _k=\boldsymbol{u}^{\top }\boldsymbol{v}$.

It follows immediately from (4.5), (4.7) and (4.8) that $g_*(1)=g_I(1)=g_0(1)$ $(=1-a-\boldsymbol{u}^{\top }\boldsymbol{v})$, whence $R_*=1 \iff R_I=1 \iff R_0=1$. The inequalities involving $R_*, R_I$ and $R_0$ in part (b) also follow immediately, since $g_*(\lambda ) \le g_I(\lambda ) \le g_0(\lambda )$ if $\lambda >1$ and $g_*(\lambda ) \ge g_I(\lambda ) \ge g_0(\lambda )$ if $\lambda <1$. $\square$

##### Remark 4.1

It follows immediately from the above proof that the inequalities between $R_*$ and $R_I$ are strict if and only if $\boldsymbol{u}^{\top }\boldsymbol{v}>0 \iff \nu _2>0$, i.e. some household transmission is possible, and the inequalities between $R_I$ and $R_0$ are strict if and only if $\nu _3>0$, i.e. generation-2 cases are possible for at least one household category. Similar arguments to the proof of Ball et al. (2016), Theorem 1, show that $R_I>R_V$ unless $\nu _3=0$ and $R_V > R_0$ unless $\nu _4=0$. (Note that $\nu _k=0$ implies $\nu _l=0$ for all $l \ge k$.)

##### Remark 4.2

In the highly locally infectious epidemic (see Sect. 6), all susceptibles in a household are infected by the primary case, so $\nu _3=0$, whence $R_V=R_I$ and the critical vaccination coverage $p_C=1-R_I^{-1}$.

For a growing epidemic, which is not highly locally infectious, $p_C=1-R_0^{-1}$ if $n_{\max } \le 3$, otherwise $1-R_0^{-1}< p_C < 1-R_I^{-1}$.

### Early exponential growth rate *r*

The early exponential growth rate is often used in estimating reproduction numbers in an emerging epidemic, see, for example, Wallinga and Lipsitch (2007) and, in a households model setting, Fraser (2007). Recall that individuals in the branching process $\mathcal {B}$ correspond to single-household epidemics and an individual in $\mathcal {B}$ has a child whenever a global contact emanates from the corresponding single-household epidemic. Recall also the augmented single-household epidemic $\hat{\mathcal {E}}_{\boldsymbol{n}, i}(\Lambda ^L(\boldsymbol{n}), \boldsymbol{\beta })$, defined in Sect. 4.1, i.e. the epidemic $\mathcal {E}_{\boldsymbol{n}, i}(\Lambda ^L(\boldsymbol{n}))$ together with the times when global contacts emanate from it. Provided the number of households is sufficiently large, the early exponential growth rate of the epidemic $\mathcal {E}$, defined in Sect. 2, may be approximated by the Malthusian parameter, *r* say, of the branching process $\mathcal {B}$ run in real time. For most multitype branching processes calculation of *r* is rather unwieldy and involves the solution of a $J \times J$ determinantal equation (see, for example, Doney (1976)). However, it is simpler in the proportionate global mixing setting, since $\mathcal {B}$ is effectively single-type and only a one-dimensional equation needs solving.

For $i \in \mathcal {J}$ and $\boldsymbol{n}\in \mathcal {N}_i$, let $\beta _{\boldsymbol{n}, i}(t)$ $(t \ge 0)$ be the intensity function of the point process of global contacts emanating from $\hat{\mathcal {E}}_{\boldsymbol{n}, i}(\Lambda ^L(\boldsymbol{n}), \boldsymbol{\beta })$, given that epidemic starts at time $t=0$. Recall that, apart from the ancestor(s), any individual in $\mathcal {B}$ independently has type $(i, \boldsymbol{n})$ with probability $\kappa _i \gamma _i \alpha _i(\boldsymbol{n})$. It follows that *r* is given by the unique positive solution of

4.9 $$\begin{aligned} \int _0^{\infty } {\textrm{e}}^{-rt}\beta (t)\,\textrm{d}t=1, \end{aligned}$$

where

4.10 $$\begin{aligned} \beta (t)=\sum _{i=1}^J \kappa _i \gamma _i \sum _{\boldsymbol{n}\in \mathcal {N}_i} \alpha _i(\boldsymbol{n}) \beta _{\boldsymbol{n}, i}(t). \end{aligned}$$

Calculation of $\beta _{\boldsymbol{n}, i}(t)$ is generally difficult, though if the infectious periods are exponentially distributed, $\int _0^{\infty } {\textrm{e}}^{-rt} \alpha _i(\boldsymbol{n}) \beta _{\boldsymbol{n}, i}(t)\,\textrm{d}t$ can be evaluated using the matrix method described in Pellis et al. (2011), Sect. 4.2. As in Sect. 4.2.3, the calculations simplify appreciably if local infection is homogeneously mixing.

### Probability of major outbreak

As noted in Sect. 4.2.1, the asymptotic probability of a major outbreak is $1-p_\textrm{ext}$, where $p_\textrm{ext}$ is the probability that the branching process $\mathcal {B}$ goes extinct. We now outline the calculation of $p_\textrm{ext}$. Note that $p_\textrm{ext}$ depends on the number and configuration of initial infectives.

Suppose first that there is a single initial infective, who is chosen by first sampling its type *j* from $P_i$ $(i \in \mathcal {J})$ and then choosing an individual uniformly at random from the $N_j$ type-*j* individuals in the population. Then all individuals in $\mathcal {B}$ have the same offspring random variable, $\tilde{X}$ say, and $R_*=\mathbb {E}[\tilde{X}]$. For $i \in \mathcal {J}$ and $\boldsymbol{n}\in \mathcal {N}_i$, let $Z^G_{\boldsymbol{n}, i}$ be the number of global contacts that emanate from the augmented single-household epidemic $\hat{\mathcal {E}}_{\boldsymbol{n}, i}(\Lambda ^L(\boldsymbol{n}), \boldsymbol{\beta })$ defined in Sect. 4.1. Then $\tilde{X}$ is a mixture of $Z^G_{\boldsymbol{n}, i}$ $(i \in \mathcal {J}, \boldsymbol{n}\in \mathcal {N}_i)$, with $\mathbb {P}(\tilde{X}=Z^G_{\boldsymbol{n}, i})=P_i \alpha _i(\boldsymbol{n})=\kappa _i\gamma _i \alpha _i(\boldsymbol{n})$ $(i \in \mathcal {J}, \boldsymbol{n}\in \mathcal {N}_i)$. Let $f_{\tilde{X}}(s)=\mathbb {E}[s^{\tilde{X}}]$ $(0 \le s \le 1)$ be the probability-generating function (PGF) of $\tilde{X}$. Then

$$ f_{\tilde{X}}(s)=\sum _{i=1}^J \kappa _i\gamma _i \sum _{\boldsymbol{n}\in \mathcal {N}_i} \alpha _i(\boldsymbol{n}) f_{Z^G_{\boldsymbol{n}, i}}(s), $$

where $f_{Z^G_{\boldsymbol{n}, i}}(s)=\mathbb {E}\left[ s^{Z^G_{\boldsymbol{n}, i}}\right]$ is the PGF of $Z^G_{\boldsymbol{n}, i}$. Let $\tilde{p}_\textrm{ext}$ denote the extinction probability of $\mathcal {B}$, with this choice of initial condition. By standard branching process theory, $\tilde{p}_\textrm{ext}$ is given by the smallest solution of $f_{\tilde{X}}(s)=s$ in [0, 1] and $\tilde{p}_\textrm{ext}<1$ if and only if $R_*>1$.

Suppose instead that the epidemic is initiated by a single type-*i* individual chosen uniformly at random from all type-*i* individuals in the population and let $p^{(i)}_\textrm{ext}$ be the corresponding extinction probability of $\mathcal {B}$. The offspring random variable, *X* say, of the ancestor in $\mathcal {B}$ is now a mixture of $Z^G_{\boldsymbol{n}, i}$ $(\boldsymbol{n}\in \mathcal {N}_i)$ with $\mathbb {P}(X=Z^G_{\boldsymbol{n}, i})=\alpha _i(\boldsymbol{n})$ $(\boldsymbol{n}\in \mathcal {N}_i)$. All subsequent individuals in $\mathcal {B}$ have offspring random variable $\tilde{X}$, so taking expectations with respect to *X* yields

$$ p^{(i)}_\textrm{ext}=\sum _{\boldsymbol{n}\in \mathcal {N}_i} \alpha _i(\boldsymbol{n}) f_{Z^G_{\boldsymbol{n}, i}}(\tilde{p}_\textrm{ext})\qquad (i \in \mathcal {J}). $$

Other choices for the initial infective can be treated in a similar fashion, as can cases with more than one initial infective, though it is more complicated if there are two or more initial infectives in the same household.

To calculate $\tilde{p}_\textrm{ext}$ and $p^{(i)}_\textrm{ext}$ $(i \in \mathcal {J})$ we need $f_{Z^G_{\boldsymbol{n}, i}}(s)$ $(i \in \mathcal {J}, \boldsymbol{n}\in \mathcal {N}_i, 0 \le s \le 1)$. Recall from Sect. 4.2.1 that, in the notation of Sect. 3.1, $Z^G_{\boldsymbol{n}, i}$ has a mixed-Poisson distribution with (random) mean distributed as $\sum _{j=1}^J \beta _j A_j$. Hence, for $0 \le s \le 1$,

$$\begin{aligned} f_{Z^G_{\boldsymbol{n}, i}}(s)=\mathbb {E}\left[ s^{Z^G_{\boldsymbol{n}, i}}\right]&=\mathbb {E}\left[ \mathbb {E}\left[ s^{Z^G_{\boldsymbol{n}, i}}\left| \right. A_1, A_2, \dots , A_J\right] \right] \\&=\mathbb {E}\left[ \exp \left( -(1-s)\sum _{j=1}^J\beta _jA_j\right) \right] \\&=\phi _{\boldsymbol{n},i}((1-s)\boldsymbol{\beta }), \end{aligned}$$

where $\phi _{\boldsymbol{n},i}(\boldsymbol{\theta })$ $(\boldsymbol{\theta }=(\theta _1, \theta _2, \dots , \theta _J)^{\top } \in \mathbb {R}_+^J)$ is the joint Laplace transform of $(A_1, A_2, \dots , A_J)$. A formula for $\phi _{\boldsymbol{n},i}(\boldsymbol{\theta })$ in terms of multivariate Gontcharoff polynomials is given in (10.3) in the Supplementary Information, Sect. 10.

## Final outcome of a major outbreak

### Fraction infected of each type

Suppose that *N* is large, there are few initial infectives and a major outbreak occurs. For $j \in \mathcal {J}$, let $\Upsilon _j$ be the sum of the infectious periods of all type-*j* individuals infected in the epidemic (the severity owing to type-*j* individuals). Let $\Upsilon =\sum _{j=1}^J \beta _j \Upsilon _j$ and $\delta =\Upsilon /N$. Thus, $\Upsilon$ is a weighted severity of the epidemic and $\delta$ a scaled weighted severity. We use a heuristic argument to derive a balance equation for $\delta$ in the limit as $N \rightarrow \infty$, from which other properties of the final outcome of a major outbreak follow easily.

Consider a typical type-*i* individual who is susceptible at the start of the epidemic. For $j \in \mathcal {J}$, the probability that this type-*i* individual avoids global infection from all type-*j* infectives during the epidemic is $\exp (-\beta _j \kappa _i \Upsilon _j/N)$, so the probability that they avoid global infection throughout the epidemic is

$$ \pi _i=\prod _{j=1}^J \exp \left( -\beta _j \kappa _i \Upsilon _j/N\right) =\exp \left( -\kappa _i \sum _{j=1}^J \beta _j \Upsilon _j/N\right) ={\textrm{e}}^{-\delta \kappa _i}. $$

Note that $\delta$ can be interpreted as the expected contribution to the weighted severity made by an individual, $i_*$ say, chosen uniformly at random from the population. As there are few initial infectives, the probability that $i_*$ belongs to an initially fully susceptible household is close to one for large *N*. For $\boldsymbol{n}\in \mathcal {N}$, let $\tilde{\alpha }_{\boldsymbol{n}}=\Vert \boldsymbol{n}\Vert m_{\boldsymbol{n}}/N$ be the probability that $i_*$ resides in a category-$\boldsymbol{n}$ household. Suppose that $i_*$ resides in a category-$\boldsymbol{n}$ household. In the limit $N \rightarrow \infty$, individuals avoid global infection independently, so the final outcome of the epidemic within that household is distributed as the final outcome of $\tilde{\mathcal {E}}_{\boldsymbol{n}}(\Lambda ^L(\boldsymbol{n}), \boldsymbol{\pi })$, defined in Sect. 3.2, with $\boldsymbol{\pi }=({\textrm{e}}^{-\delta \kappa _1},{\textrm{e}}^{-\delta \kappa _2},\dots ,{\textrm{e}}^{-\delta \kappa _J})$. Given $i_*$ belongs to a category-$\boldsymbol{n}$ household, the probability $i_*$ has type *j* is $n_j/\Vert \boldsymbol{n}\Vert$. Suppose $i_*$ has type *j*. Then the probability that $i_*$ is infected in $\tilde{\mathcal {E}}_{\boldsymbol{n}}(\Lambda ^L(\boldsymbol{n}), \boldsymbol{\pi })$ is $n_j^{-1} \tilde{\mu }_{\boldsymbol{n},j}(\Lambda ^L(\boldsymbol{n}),\boldsymbol{\pi })$ and the contribution of $i_*$ to $\delta$ is $n_j^{-1} \tilde{\mu }_{\boldsymbol{n},j}(\Lambda ^L(\boldsymbol{n}),\boldsymbol{\pi }) \mu _I^{(j)}\beta _j$. Let $\boldsymbol{\kappa }=(\kappa _1, \kappa _2, \dots , \kappa _J)$ and ${\textrm{e}}^{-\delta \boldsymbol{\kappa }}=({\textrm{e}}^{-\delta \kappa _1},{\textrm{e}}^{-\delta \kappa _2},\dots ,{\textrm{e}}^{-\delta \kappa _J})$. Then the above argument yields

5.1 $$\begin{aligned} \delta&=\sum _{\boldsymbol{n}\in \mathcal {N}_j} \tilde{\alpha }_{\boldsymbol{n}}\sum _{j=1}^J \frac{n_j}{\Vert \boldsymbol{n}\Vert }n_j^{-1} \tilde{\mu }_{\boldsymbol{n},j}(\Lambda ^L(\boldsymbol{n}),\boldsymbol{\pi }) \mu _I^{(j)}\beta _j \nonumber \\&=\sum _{\boldsymbol{n}\in \mathcal {N}}\frac{\tilde{\alpha }_{\boldsymbol{n}}}{\Vert \boldsymbol{n}\Vert }\sum _{j=1}^J \tilde{\mu }_{\boldsymbol{n},j}(\Lambda ^L(\boldsymbol{n}),{\textrm{e}}^{-\delta \boldsymbol{\kappa }}) \mu _I^{(j)}\beta _j, \end{aligned}$$

since $$\tilde{\mu }_{\boldsymbol{n},j}(\Lambda ^L(\boldsymbol{n}),{\textrm{e}}^{-\delta \boldsymbol{\kappa }})=0$$ if $$n_j=0$$.

Observe that $\delta =0$ is always a solution of (5.1). We show at the end of Sect. 5.2 that a (unique) second solution $\delta ^* \in (0,\infty )$, giving the scaled weighted severity in the event of a major outbreak, exists if and only if $R_*>1$.

For $i \in \mathcal {J}$, let $\tilde{z}_i$ be the fraction of type-*i* individuals that are infected in a major outbreak. Then $\tilde{z}_i$ is also the probability that an individual, $j_*$ say, chosen uniformly at random from all type-*i* individuals in the population is infected by a major outbreak. Now $j_*$ belongs to a category-$\boldsymbol{n}$ household with probability $\alpha _i(\boldsymbol{n})$, so conditioning on $j_*$’s household category and arguing as above yields

5.2 $$\begin{aligned} \tilde{z}_i=\sum _{\boldsymbol{n}\in \mathcal {N}_i} \alpha _i(\boldsymbol{n}) \tilde{\mu }_{\boldsymbol{n},i}(\Lambda ^L(\boldsymbol{n}),{\textrm{e}}^{-\delta ^* \boldsymbol{\kappa }})/n_i \qquad (i \in \mathcal {J}). \end{aligned}$$

#### Remark 5.1

Other properties of the final outcome of a major outbreak, such as the fraction of households of a given category that are infected or the fraction of households with no type-1 individuals infected, can be derived in a similar fashion using properties of the epidemics $\tilde{\mathcal {E}}_{\boldsymbol{n}}(\Lambda ^L(\boldsymbol{n}), {\textrm{e}}^{-\delta ^* \boldsymbol{\kappa }})$ $(\boldsymbol{n}\in \mathcal {N})$. For example, the probability that a given fully-susceptible category-$\boldsymbol{n}$ household avoids infection in a major outbreak is given by the probability that no-one is infected in the epidemic $\tilde{\mathcal {E}}_{\boldsymbol{n}}(\Lambda ^L(\boldsymbol{n}), {\textrm{e}}^{-\delta ^* \boldsymbol{\kappa }})$, which equals $\exp \left( -\delta ^*\sum _{i=1}^J n_i \kappa _i\right) =\exp (-\delta ^*\boldsymbol{n}\boldsymbol{\kappa }^{\top })$. Thus the fraction of households infected by a major outbreak is $z_E=\sum _{\boldsymbol{n}\in \mathcal {N}} \alpha _{\boldsymbol{n}}(1-{\textrm{e}}^{-\delta ^*\boldsymbol{n}\boldsymbol{\kappa }^{\top }})$. This is made fully rigorous in the Supplementary Information, Sect. 9.2.2; see Theorem 9.4, which shows that the fraction of households infected by a major outbreak converges in distribution to a point mass at $z_E$ as $v \rightarrow \infty$, and Remark 9.6.

### Backward branching process

We now describe how $\tilde{z}_i$ $(i \in \mathcal {J})$ can be found using the extinction probability of a (backward) *J*-type branching process, which again is essentially single-type owing to proportionate global mixing. A key tool is the concept of a *susceptibility set* (see, for example, Ball and Neal (2002) and Ball (2019)), which corresponds to the *in-component* of an individual in an epidemic percolation network (see, for example, Kenah and Robins (2007) and Kenah and Miller (2011)).

Consider first the directed random graph $\mathcal {G}_{\boldsymbol{n}}(\Lambda ^L(\boldsymbol{n}))$ defined in Sect. 3.3. For $i \in \mathcal {J}$, label the type-*i* individuals in the household $(i,1), (i,2), \dots , (i,n_i)$. Let $\mathcal {V}^{(\boldsymbol{n})}=\{(i,j):i=1,2,\dots , J, j=1,2,\dots , n_i\}$ be the vertex set of $\mathcal {G}_{\boldsymbol{n}}(\Lambda ^L(\boldsymbol{n}))$ and, for $i \in \mathcal {J}$, let $\mathcal {V}^{(\boldsymbol{n})}_i=\{(i,j):j=1,2,\dots , n_i\}$ be the set of type-*i* vertices. For $((i,j), (i', j')) \in (\mathcal {V}^{(\boldsymbol{n})})^2$, write $(i,j) \leadsto (i',j')$ if there is a chain of directed edges from (*i*, *j*) to $(i',j')$ in $\mathcal {G}_{\boldsymbol{n}}(\Lambda ^L(\boldsymbol{n}))$, with the convention that $(i,j) \leadsto (i,j)$. For $(i,j) \in \mathcal {V}^{(\boldsymbol{n})}$, let $\mathcal {S}^{(\boldsymbol{n})}_{ij}=\{(i',j') \in \mathcal {V}^{(\boldsymbol{n})}: (i',j') \leadsto (i,j)\}$ be the local susceptibility set of individual (*i*, *j*) and, for $k \in \mathcal {J}$, let $\mathcal {S}^{(\boldsymbol{n})}_{ij}(k)= \mathcal {S}^{(\boldsymbol{n})}_{ij} \cap \mathcal {V}^{(\boldsymbol{n})} _k$ be the set of type-*k* individuals in (*i*, *j*)’s local susceptibility set and $S_{ij}^{(\boldsymbol{n})}(k)=|\mathcal {S}^{(\boldsymbol{n})}_{ij}(k)|$. (Note that $\mathcal {S}^{(\boldsymbol{n})}_{ij}$ is defined only if $\boldsymbol{n}\in \mathcal {N}_i$ and $\mathcal {S}^{(\boldsymbol{n})}_{ij}(k)$ is the empty set (and $S_{ij}^{(\boldsymbol{n})}(k)=0$) if $\boldsymbol{n}\notin \mathcal {N}_k$.)

Note that if $\mathcal {G}_{\boldsymbol{n}}(\Lambda ^L(\boldsymbol{n}))$ is used in the construction of a realisation of the epidemic $\tilde{\mathcal {E}}(\Lambda ^L(\boldsymbol{n}), \boldsymbol{\pi })$, as described in Sect. 3.3, then individual (*i*, *j*) avoids infection in that epidemic if and only if all members of (*i*, *j*)’s local susceptibility set $\mathcal {S}^{(\boldsymbol{n})}_{ij}$ avoid external infection. Recall from Sect. 5.1 that, if $\boldsymbol{n}\in \mathcal {N}_i$, then the probability that a given type-*i* individual is infected in $\tilde{\mathcal {E}}(\Lambda ^L(\boldsymbol{n}), \boldsymbol{\pi })$ is given by $n_i^{-1} \tilde{\mu }_{\boldsymbol{n},i}(\Lambda ^L(\boldsymbol{n}),\boldsymbol{\pi })$. Thus,

5.3 $$\begin{aligned} n_i^{-1} \tilde{\mu }_{\boldsymbol{n},i}(\Lambda ^L(\boldsymbol{n}),\boldsymbol{\pi })=\mathbb {E}\left[ \prod _{k=1}^J\pi _k^{S_{i1}^{(\boldsymbol{n})}(k)}\right] \qquad (i \in \mathcal {J}, \boldsymbol{n}\in \mathcal {N}_i). \end{aligned}$$

An analogous directed random graph $\mathcal {G}_{\mathcal {E}}$ can be constructed for the epidemic $\mathcal {E}$ defined in Sect. 2 and the susceptibility set $\mathcal {S}_{i_*}$ of an individual $i_*$ can be defined accordingly. As with the network-households model in Ball et al. (2010), the susceptibility set $\mathcal {S}_{i_*}$ can be constructed by considering successive generations of households and approximated by a Galton-Watson branching process, $\mathcal {B}_B$ say. First, we construct the local susceptibility set, $\mathcal {S}_{i_*}^L$ say, of $i_*$ in their household. This corresponds to the ancestor in $\mathcal {B}_B$. Then, we look to see which individuals, who are not in $\mathcal {S}_{i_*}^L$, make global contact with members of $\mathcal {S}_{i_*}^L$. With high probability, these individuals will be in distinct households (and not belong to $i_*$’s household) and their households correspond to the first generation in $\mathcal {B}_B$. We then construct the local susceptibility sets of those individuals in their households, and continue the process in the obvious fashion. Suppose that $i_*$ has type *i*. Then the (asymptotic) fraction of type-*i* individuals infected by a major outbreak is given by the probability that $\mathcal {B}_B$ does not go extinct. (See Ball et al. (2014) for a formal proof for an SIR epidemic on a random intersection graph.)

Consider a typical type-*i* individual, $i_*$ say. For $j \in \mathcal {J}$, let $Z_{ji}$ be the number of type-*j* individuals that make global contact with $i_*$. Then $Z_{ji}$ $(j \in \mathcal {J})$ are independent, with $Z_{ji} \sim \textrm{Bin}(N_j,1- \phi _{I^{(j)}}(\lambda _{ji}^G/N))$, if $j \ne i$, and $Z_{ii} \sim \textrm{Bin}(N_i-1,1- \phi _{I^{(i)}}(\lambda _{ii}^G/N))$, where $\phi _{I^{(j)}}(\theta )=\mathbb {E} [\exp (-\theta I^{(j)})]$ $(\theta \ge 0)$ is the Laplace transform of $I^{(j)}$ and $\textrm{Bin}(n,p)$ denotes a Binomial random variable with *n* trials and success probability *p*. Recall that $\lambda _{ji}^G=\beta _j \kappa _i$ $(j,i \in \mathcal {J})$ and $\gamma _j=N_j/N$ $(j \in \mathcal {J})$. It follows that, in the limit $N \rightarrow \infty$, the random variables $Z_{ji}$ $(j \in \mathcal {J})$ are independent, with $Z_{ji} \sim \textrm{Po}(\gamma _j \beta _j \mu _I^{(j)} \kappa _i)$, where $\textrm{Po}(\mu )$ denotes a Poisson random variable with mean $\mu$. Let $Z_i=Z_{1i}+Z_{2i}+\dots +Z_{Ji}$ be the total number of individuals that make global contact with $i_*$. Then using standard properties of Poisson random variables, $Z_i \sim \textrm{Po}(c\kappa _i)$, where $c=\sum _{j \in \mathcal {J}} \gamma _j \beta _j \mu _I^{(j)}$ and, conditional upon $Z_i$, each global contact with $i_*$ is from a type-*j* individual independently with probability $P^B_j=\gamma _j \beta _j \mu _I^{(j)}/c$. Observe that $P^B_j$ does not depend on the type *i* of $i_*$. Thus, in the construction of the backward branching process $\mathcal {B}_B$, it is sufficient to consider the total number of individuals that make global contact with members of an individual’s local susceptibility set. Each such contact is from a type-*j* individual independently with probability $P^B_j$ and given it is from a type-*j* individual, it is from an individual who resides in a category-$\boldsymbol{n}$ household with probability $\alpha _j(\boldsymbol{n})$ $(\boldsymbol{n}\in \mathcal {N}_j)$. Note the similarity with the construction of the forward branching process $\mathcal {B}$ in Sect. 4.1.

For $i \in \mathcal {J}$ and $\boldsymbol{n}\in \mathcal {N}_i$, let $\tilde{Z}_{\boldsymbol{n},i}^G$ be the total number of individuals that make global contact with members of the local susceptibility set of a typical type-*i* individual residing in a category-$\boldsymbol{n}$ household. The above argument shows that (asymptotically) $\tilde{Z}_{\boldsymbol{n},i}^G$ has a mixed-Poisson distribution with random mean distributed as $c\sum _{j \in \mathcal {J}} S_{i1}^{(\boldsymbol{n})}(j)\kappa _j$. Hence,

5.4 $$\begin{aligned} f_{\tilde{Z}^G_{\boldsymbol{n}, i}}(s)=\mathbb {E}\left[ s^{\tilde{Z}^G_{\boldsymbol{n}, i}}\right]&=\mathbb {E}\left[ \mathbb {E}\left[ s^{\tilde{Z}^G_{\boldsymbol{n}, i}}\left| \right. S_{i1}^{(\boldsymbol{n})}(1), S_{i1}^{(\boldsymbol{n})}(2), \dots , S_{i1}^{(\boldsymbol{n})}(J)\right] \right] \nonumber \\&=\mathbb {E}\left[ \exp \left( -c (1-s) \sum _{j \in \mathcal {J}} S_{i1}^{(\boldsymbol{n})}(j)\kappa _j\right) \right] \nonumber \\&=1-\frac{1}{n_i} \tilde{\mu }_{\boldsymbol{n},i}(\Lambda ^L(\boldsymbol{n}),{\textrm{e}}^{-c(1-s) \boldsymbol{\kappa }}) \qquad (i \in \mathcal {J}, \boldsymbol{n}\in \mathcal {N}_i), \end{aligned}$$

where the final line has used (5.3).

Let $\tilde{X}^B$ denote the offspring random variable of an individual in $\mathcal {B}_B$ who is not the ancestor. The above argument shows that $\tilde{X}^B$ is a mixture of $\tilde{Z}_{\boldsymbol{n},i}^G$ $(i \in \mathcal {J}, \boldsymbol{n}\in \mathcal {N}_i)$, with $\mathbb {P}(\tilde{X}^B=\tilde{Z}_{\boldsymbol{n},i}^G)=P^B_i \alpha _i(\boldsymbol{n})$. Hence, recalling that $P^B_i=\gamma _i \beta _i \mu _I^{(i)}/c$, the PGF of $\tilde{X}^B$ is given by

$$ f_{\tilde{X}^B}(s)=1-\sum _{i \in \mathcal {J}} \frac{\gamma _i \beta _i \mu _I^{(i)}}{c}\sum _{\boldsymbol{n}\in \mathcal {N}_i} \alpha _i(\boldsymbol{n}) \frac{1}{n_i} \tilde{\mu }_{\boldsymbol{n},i}(\Lambda ^L(\boldsymbol{n}),{\textrm{e}}^{-c(1-s) \boldsymbol{\kappa }})\qquad (0 \le s \le 1). $$

Let $\tilde{p}_\textrm{ext}^B$ be the extinction probability of the branching process in which all individuals have offspring random variable $\tilde{X}^B$. Then $\tilde{p}_\textrm{ext}^B$ is given by the smallest solution in [0, 1] of $f_{\tilde{X}^B}(s)=s$ and hence also of

5.5 $$\begin{aligned} c(1-s)=\sum _{i \in \mathcal {J}} \gamma _i \beta _i \mu _I^{(i)} \sum _{\boldsymbol{n}\in \mathcal {N}_i} \alpha _i(\boldsymbol{n}) \frac{1}{n_i} \tilde{\mu }_{\boldsymbol{n},i}(\Lambda ^L(\boldsymbol{n}),{\textrm{e}}^{-c(1-s) \boldsymbol{\kappa }}). \end{aligned}$$

For $i \in \mathcal {J}$, a type-*i* individual chosen uniformly at random from the population resides in a category-$\boldsymbol{n}$ household with probability $\alpha _i(\boldsymbol{n})$. Hence, conditioning appropriately on the number of offspring of the ancestor in $\mathcal {B}_B$,

5.6 $$\begin{aligned} \tilde{z}_i&=1-\sum _{\boldsymbol{n}\in \mathcal {N}_i} \alpha _i(\boldsymbol{n}) f_{\tilde{Z}^G_{\boldsymbol{n}, i}}(\tilde{p}_\textrm{ext}^B)\nonumber \\&= \sum _{\boldsymbol{n}\in \mathcal {N}_i} \alpha _i(\boldsymbol{n}) \frac{1}{n_i} \tilde{\mu }_{\boldsymbol{n},i}(\Lambda ^L(\boldsymbol{n}),{\textrm{e}}^{-c(1-\tilde{p}_\textrm{ext}^B) \boldsymbol{\kappa }}) \qquad (i \in \mathcal {J}), \end{aligned}$$

using (5.4).

Note from (5.5) that $\delta =c(1-s)$ satisfies (5.1), since

5.7 $$\begin{aligned} \gamma _i \alpha _i(\boldsymbol{n}) \frac{1}{n_i}=\frac{N_i}{N} \frac{n_i m_{\boldsymbol{n}}}{N_i} \frac{1}{n_i}=\frac{m_{\boldsymbol{n}}}{N}=\frac{\tilde{\alpha }_{\boldsymbol{n}}}{\Vert \boldsymbol{n}\Vert }. \end{aligned}$$

Thus, $\delta ^*=c(1-\tilde{p}_\textrm{ext}^B)$ and (5.6) coincides with (5.2).

#### Remark 5.2

Other asymptotic final outcome quantities of a major outbreak can be derived in a similar fashion by considering the offspring of the corresponding ancestor in $\mathcal {B}_B$. For example, consider an initially fully-susceptible category-$\boldsymbol{n}$ household, as in Remark 5.1. The offspring of the ancestor in $\mathcal {B}_B$ is given by the total number of global contacts made with any individual in that household, which asymptotically has a Poisson distribution with mean $c\sum _{i \in \mathcal {J}} \kappa _i n_i=c \boldsymbol{n}\boldsymbol{\kappa }^{\top }$. The probability of that household avoiding infection in a major outbreak is given by the extinction probability of $\mathcal {B}_B$, which, taking expectation with respect to the numbers of offspring of the *J* types of the ancestor, is given by $\exp (-(1-\tilde{p}_\textrm{ext}^B)c\boldsymbol{n}\boldsymbol{\kappa }^{\top })=\exp (-\delta ^*\boldsymbol{n}\boldsymbol{\kappa }^{\top })$, in agreement with Remark 5.1.

We end this subsection by showing that $\mathbb {E}[\tilde{X}^B]=R_*$, so $\tilde{p}_\textrm{ext}^B<1$ (and $\delta ^*>0$) if and only if $R_*>1$. Recalling the mixture representation of $\tilde{X}^B$ and the mixed Poisson distribution of $\tilde{Z}_{\boldsymbol{n},i}^G$ given above,

$$\begin{aligned} \mathbb {E}[\tilde{X}^B]&=\sum _{i \in \mathcal {J}} \sum _{\boldsymbol{n}\in \mathcal {N}_i}P^B_i \alpha _i(\boldsymbol{n}) c \sum _{j \in \mathcal {J}} \mathbb {E}[S_{i1}^{(\boldsymbol{n})}(j)]\kappa _j\\&=\sum _{i \in \mathcal {J}} \gamma _i \beta _i \mu _I^{(i)} \sum _{\boldsymbol{n}\in \mathcal {N}_i} \alpha _i(\boldsymbol{n})\sum _{j \in \mathcal {J}} \mathbb {E}[S_{i1}^{(\boldsymbol{n})}(j)]\kappa _j. \end{aligned}$$

Note that $\mathbb {E}[S_{i1}^{(\boldsymbol{n})}(j)]=0$ if $\boldsymbol{n}\notin \mathcal {N}_j$. For $i \in \mathcal {J}$ and $\boldsymbol{n}\in \mathcal {N}_i \cap \mathcal {N}_j$,

$$\begin{aligned} n_i \mathbb {E}[S_{i1}^{(\boldsymbol{n})}(j)]=\sum _{k=1}^{n_i}\mathbb {E}[S_{ik}^{(\boldsymbol{n})}(j)]&=\sum _{k=1}^{n_i} \sum _{\ell =1}^{n_j}\mathbb {P}((j,\ell )\leadsto (i,k))\\&=n_j \sum _{k=1}^{n_i}\mathbb {P}((j,1)\leadsto (i,k))=n_j \mu _{\boldsymbol{n},j,i}(\Lambda ^L(\boldsymbol{n})), \end{aligned}$$

so

$$\begin{aligned} \mathbb {E}[\tilde{X}^B]=\sum _{i \in \mathcal {J}} \gamma _i \beta _i \mu _I^{(i)} \sum _{j \in \mathcal {J}} \sum _{\boldsymbol{n}\in \mathcal {N}_i \cap \mathcal {N}_j} \alpha _i(\boldsymbol{n}) \kappa _j \frac{n_j}{n_i} \mu _{\boldsymbol{n},j,i}(\Lambda ^L(\boldsymbol{n})). \end{aligned}$$

Now, using (5.7),

$$ \gamma _i \alpha _i(\boldsymbol{n}) \frac{n_j}{n_i}=\frac{m_{\boldsymbol{n}}n_j}{N}=\frac{m_{\boldsymbol{n}}n_j}{N_j} \frac{N_j}{N}=\alpha _j(\boldsymbol{n})\gamma _j, $$

so, since $\mu _{\boldsymbol{n},j,i}(\Lambda ^L(\boldsymbol{n}))=0$ if $\boldsymbol{n}\notin \mathcal {N}_i$,

$$\begin{aligned} \mathbb {E}[\tilde{X}^B]=\sum _{i \in \mathcal {J}} \beta _i \mu _I^{(i)} \sum _{j \in \mathcal {J}} \sum _{\boldsymbol{n}\in \mathcal {N}_j} \alpha _j(\boldsymbol{n})\kappa _j \gamma _j \mu _{\boldsymbol{n},j,i}(\Lambda ^L(\boldsymbol{n}))=R_*. \end{aligned}$$

### Central limit theorem

We now describe a multivariate central limit theorem for quantities associated with the final outcome of a major outbreak. Consider the epidemic $\mathcal {E}$ defined in Sect. 2. For $\boldsymbol{n}\in \mathcal {N}$, give the $m_{\boldsymbol{n}}$ households of category $\boldsymbol{n}$ the labels $(\boldsymbol{n},1), (\boldsymbol{n},2),\dots , (\boldsymbol{n},m_{\boldsymbol{n}})$. For $i \in \mathcal {J}$ and $k=1,2,\dots , m_{\boldsymbol{n}}$ let $A_i^{(\boldsymbol{n},k)}$ be the sum of the infectious periods of all type-*i* individuals in household $(\boldsymbol{n}, k)$ that are infected during $\mathcal {E}$. Let $A^{(\boldsymbol{n},k)}=\sum _{i=1}^J \beta _i A_i^{(\boldsymbol{n},k)}$ be the contribution of household $(\boldsymbol{n}, k)$ to the weighted severity of the epidemic $\mathcal {E}$. Define also on each household a vector $\boldsymbol{R}'=(R_1', R_2',\dots , R_p')^{\top }$ of *p* final outcome quantities, with $\boldsymbol{R}^{(\boldsymbol{n},k)}$ being the vector for household $(\boldsymbol{n}, k)$. Let

$$ A=\sum _{\boldsymbol{n}\in \mathcal {N}}\sum _{k=1}^{m_{\boldsymbol{n}}} A^{(\boldsymbol{n},k)} \qquad {\text {and}}\qquad \boldsymbol{R}=(R_1, R_2, \dots , R_p)^{\top }=\sum _{\boldsymbol{n}\in \mathcal {N}}\sum _{k=1}^{m_{\boldsymbol{n}}} \boldsymbol{R}^{(\boldsymbol{n},k)}. $$

The final outcome quantities can be any quantity for which summing over all households to get a corresponding quantity for the epidemic $\mathcal {E}$ makes sense. For example, $R_1'$ could be the number of type-1 individuals infected in the household, in which case $R_1$ is the number of type-1 individuals infected in $\mathcal {E}$; $R_2'$ could be the number of individuals infected in the household, in which case $R_2$ is number of individuals infected in $\mathcal {E}$; and $R_3'$ could be the indicator function of the household being infected in $\mathcal {E}$, in which case $R_3$ is the number of households infected in $\mathcal {E}$. The weighted severity of the household is another example but we use a separate notation for that as it plays a special role in the analysis. Other examples, in an inferential setting, are the pseudo-score-statistics for parameters of interest, such as $\lambda _{11}^L(\boldsymbol{n})$ (see Ball et al. (2004), Sect. 3.1). There are numerous other possibilities.

We give a multivariate central limit theorem for $$\begin{pmatrix} \boldsymbol{R}\\ A \end{pmatrix} $$ in the event of a major outbreak. The theorem is proved by adapting the embedding approach of Scalia-Tomba (1985, 1990), along similar lines to that used in Ball et al. (1997) and Ball and Lyne (2001). The proof, given in the Supplementary Information, Sect. 9.2.3, is similar to that of Ball and Lyne (2001), Theorem 5.3, though simpler owing to the assumption of proportionate global mixing. In order to be precise about the conditions that the final outcome quantities are assumed to satisfy in the theorem, we need to give some details of the construction underlying the embedding argument.

Fix $\boldsymbol{n}\in \mathcal {N}$ and let $\mathcal {G}_{\boldsymbol{n}}(\Lambda ^L(\boldsymbol{n}))$ be a realisation of the directed random graph $\mathcal {G}_{\boldsymbol{n}}(\Lambda ^L(\boldsymbol{n}))$ defined in Sect. 3.3, with individuals labelled as in Sect. 5.2. For $i \in \mathcal {J}$ and $j=1,2,\dots , n_i$, let $I_{ij}$ be the infectious period of individual (*i*, *j*) if they were to become infected. Let $L_{ij}$ $(i \in \mathcal {J}, j=1,2,\dots , n_i)$ be an independent collection of independent exponential random variables, where $L_{ij}$ has rate $\kappa _i$ (and hence mean $\kappa _i^{-1}$). For each $t \ge 0$, we construct an epidemic $\tilde{\mathcal {E}}_{\boldsymbol{n}}(t)$ as follows. We determine first who is infected globally (i.e. externally); specifically, individual (*i*, *j*) is infected globally if and only if $L_{ij} \le t$. We then use the random directed graph $\mathcal {G}_{\boldsymbol{n}}(\Lambda ^L(\boldsymbol{n}))$ to determine who is infected locally; these local infections take place instantaneously with respect to *t*. Note that in this construction *t* refers to global infectious pressure and *not* to time. For $t \ge 0$ and $i \in \mathcal {J}$, let $\tilde{A}^{(\boldsymbol{n})}_i(t)$ denote the sum of the infectious periods of *all* type-*i* individuals infected in the epidemic $\tilde{\mathcal {E}}_{\boldsymbol{n}}(t)$, and let $A^{(\boldsymbol{n})}(t)=\sum _{i=1}^J\beta _i\tilde{A}^{(\boldsymbol{n})}_i(t)$ be the corresponding weighted severity; also let $\boldsymbol{R}^{(\boldsymbol{n},k)}(t)=(R^{(\boldsymbol{n},k)}_1(t), R^{(\boldsymbol{n},k)}_2(t), \dots , R^{(\boldsymbol{n},k)}_p(t))^{\top }$ be the vector of final outcomes $\boldsymbol{R}'$ for the epidemic $\tilde{\mathcal {E}}_{\boldsymbol{n}}(t)$.

Observe that by construction the process $A^{(\boldsymbol{n})}(t)$ $(t \ge 0)$ is a nondecreasing, piecewise constant function of *t*, with $A^{(\boldsymbol{n})}(0)=0$ and jumps can occur only when $t=L_{ij}$ for some (*i*, *j*). (A jump does not occur when $t=L_{ij}$ if individual (*i*, *j*) is infected (locally) in the epidemic $\tilde{\mathcal {E}}_{\boldsymbol{n}}(u)$ for some $u<t$.) Similarly, $R^{(\boldsymbol{n},k)}_i(t)$ $(t \ge 0)$ is piecewise constant for each $i=1,2,\dots ,p$. For each fixed $t \ge 0$, the epidemic $\tilde{\mathcal {E}}_{\boldsymbol{n}}(t)$ is a realisation of the epidemic $\tilde{\mathcal {E}}_{\boldsymbol{n}}(\Lambda ^L(\boldsymbol{n}), {\textrm{e}}^{-t\boldsymbol{\kappa }})$ defined in Sect. 3.2. Thus, $\mathbb {E}[\tilde{A}^{(\boldsymbol{n})}_i(t)]=\tilde{\mu }_{\boldsymbol{n},i}(\Lambda ^L(\boldsymbol{n}),{\textrm{e}}^{-t\boldsymbol{\kappa }}) \mu _I^{(i)}$.

For $\boldsymbol{n}\in \mathcal {N}$, let $\tilde{\mathcal {E}}_{\boldsymbol{n}}$ denote the process $\{\tilde{\mathcal {E}}_{\boldsymbol{n}}(t):t \ge 0\}$. Let $\tilde{\mathcal {E}}_{\boldsymbol{n},k}=\{\tilde{\mathcal {E}}_{\boldsymbol{n},k}(t):t \ge 0\}$ $(\boldsymbol{n}\in \mathcal {N}, k=1,2,\dots )$ be independent, with $\tilde{\mathcal {E}}_{\boldsymbol{n},k}$ distributed as $\tilde{\mathcal {E}}_{\boldsymbol{n}}$, and for $t \ge 0$, let $A^{(\boldsymbol{n},k)}(t)$ and $\boldsymbol{R}^{(\boldsymbol{n},k)}(t)$ be respectively the weighted severity and final outcome vector of $\tilde{\mathcal {E}}_{\boldsymbol{n},k}(t)$. Let

$$ A_{\bullet }(t)=\sum _{\boldsymbol{n}\in \mathcal {N}} \sum _{k=1}^{m_{\boldsymbol{n}}} A^{(\boldsymbol{n},k)}(t) \qquad {\text {and}}\qquad \boldsymbol{R}_{\bullet }(t)=\sum _{\boldsymbol{n}\in \mathcal {N}} \sum _{k=1}^{m_{\boldsymbol{n}}} \boldsymbol{R}^{(\boldsymbol{n},k)}(t) \qquad (t \ge 0). $$

We show in the Supplementary Information (see equation (9.6) and Remark 9.3) that $(A, \boldsymbol{R}^{\top })$, the weighted severity and final outcome vector of the epidemic $\mathcal {E}$, are asymptotically distributed as $(A_{\bullet }(\bar{T}_{\infty }),\boldsymbol{R}_{\bullet }(\bar{T}_{\infty })^{\top })$, where $\bar{T}_{\infty }=N^{-1} T_{\infty }$ and $T_{\infty }$ is a stopping time associated with the process $\{A_{\bullet }(t):t \ge 0\}$. The asymptotic distribution of $(A_{\bullet }(\bar{T}_{\infty }),\boldsymbol{R}_{\bullet }(\bar{T}_{\infty })^{\top })$ can be determined, since $\{(A_{\bullet }(t), \boldsymbol{R}_{\bullet }(t)^{\top }):t \ge 0\}$ is a sum of independent processes.

Before stating the theorem, some more notation is required. For $\boldsymbol{n}\in \mathcal {N}$ and $t \ge 0$, let

$$ a_{\boldsymbol{n}}(t)=\mathbb {E}[A^{(\boldsymbol{n})}(t)]=\sum _{i=1}^J\beta _i\mathbb {E}\left[ \tilde{A}^{(\boldsymbol{n})}_i(t)\right] =\sum _{i=1}^J\tilde{\mu }_{\boldsymbol{n},i}(\Lambda ^L(\boldsymbol{n}),{\textrm{e}}^{-t\boldsymbol{\kappa }}) \mu _I^{(i)}\beta _i \quad (\boldsymbol{n}\in \mathcal {N}) $$

and $\boldsymbol{r}_{\boldsymbol{n}}(t)=\mathbb {E}\left[ \boldsymbol{R}^{(\boldsymbol{n})}(t)\right]$. Also, for $t \ge 0$, let

$$\begin{aligned} c_{AA}^{(\boldsymbol{n})}(t)&={\textrm{var}}\left( A^{(\boldsymbol{n})}(t)\right) =\sum _{i=1}^J \sum _{j=1}^J \beta _i \beta _j {\textrm{cov}}\left( \tilde{A}^{(\boldsymbol{n})}_i(t), \tilde{A}^{(\boldsymbol{n})}_j(t)\right) ,\\ \boldsymbol{C}_{RR}^{(\boldsymbol{n})}(t)&={\textrm{var}}\left( \boldsymbol{R}^{(\boldsymbol{n})}(t)\right) =\left[ {\textrm{cov}}\left( R_i^{(\boldsymbol{n})}(t),R_j^{(\boldsymbol{n})}(t)\right) \right] ,\\ \boldsymbol{c}_{AR}^{(\boldsymbol{n})}(t)&=\left( {\textrm{cov}}\left( A^{(\boldsymbol{n})}(t), R_1^{(\boldsymbol{n})}(t)\right) , {\textrm{cov}}\left( A^{(\boldsymbol{n})}(t), R_2^{(\boldsymbol{n})}(t)\right) , \dots , {\textrm{cov}}\left( A^{(\boldsymbol{n})}(t), R_p^{(\boldsymbol{n})}(t)\right) \right) \end{aligned}$$

and $\boldsymbol{c}_{RA}^{(\boldsymbol{n})}(t)=\left( \boldsymbol{c}_{AR}^{(\boldsymbol{n})}(t)\right) ^{\top }$. Note that

$$ {\textrm{cov}}\left( A^{(\boldsymbol{n})}(t), R_i^{(\boldsymbol{n})}(t)\right) =\sum _{j=1}^J \beta _j {\textrm{cov}}\left( \tilde{A}^{(\boldsymbol{n})}_j(t), R_i^{(\boldsymbol{n})}(t)\right) \qquad (i=1,2,\dots ,p). $$

Let

5.8 $$\begin{aligned} \boldsymbol{C}^{(\boldsymbol{n})}(t)=\begin{pmatrix} \boldsymbol{C}_{RR}^{(\boldsymbol{n})}(t)& \boldsymbol{c}_{RA}^{(\boldsymbol{n})}(t) \\ \boldsymbol{c}_{AR}^{(\boldsymbol{n})}(t)& c_{AA}^{(\boldsymbol{n})}(t) \end{pmatrix}. \end{aligned}$$

We consider a sequence of epidemics $\mathcal {E}^{(v)}$ $(v=1,2,\dots )$, as described at the end of Sect. 4.1. Let $A^{(v)}$ and $\boldsymbol{R}^{(v)}$ be respectively the weighted severity and final outcome vector of $\mathcal {E}^{(v)}$. For $t \ge 0$, let

$$ a(t)=\sum _{\boldsymbol{n}\in \mathcal {N}} \alpha _{\boldsymbol{n}} a_{\boldsymbol{n}}(t),\quad \boldsymbol{r}(t)=\sum _{\boldsymbol{n}\in \mathcal {N}} \alpha _{\boldsymbol{n}} \boldsymbol{r}_{\boldsymbol{n}}(t)\quad {\text {and}}\quad \boldsymbol{C}(t)= \sum _{\boldsymbol{n}\in \mathcal {N}} \alpha _{\boldsymbol{n}}\boldsymbol{C}^{(\boldsymbol{n})}(t). $$

The function *a*(*t*) is strictly increasing and concave on $[0, \infty )$ and satisfies $a(0)=0$, $a^{\prime }(0)=m_HR_*$, where $m_H=\sum _{\boldsymbol{n}\in \mathcal {N}} \alpha _{\boldsymbol{n}} \Vert \boldsymbol{n}\Vert$ is the asymptotic mean household size, and $a(\infty )=\sum _{\boldsymbol{n}\in \mathcal {N}}\alpha _{\boldsymbol{n}}\sum _{i=1}^J \beta _i n_i \mu _I^{(i)}$; see Theorem 9.2 near the start of Sect. 9.2.2 in the Supplementary Information. Consider the equation

5.9 $$\begin{aligned} t=m_H^{-1}a(t). \end{aligned}$$

Observe that $t=0$ is a solution of (5.9). The above properties of *a*(*t*) imply that this is the only solution in $[0, \infty )$ if $R_* \le 1$ and, if $R_*>1$, then there is a unique solution, $\tau$ say, in $(0, \infty )$ and $a^{\prime }(\tau )<m_H$. Note that (5.9) is the same as (5.1), with $\delta$ replaced by *t*, since $\tilde{\alpha }_{\boldsymbol{n}}=m_H^{-1}\alpha _{\boldsymbol{n}}\Vert \boldsymbol{n}\Vert$ $(\boldsymbol{n}\in \mathcal {N})$, and that $\tau = \delta ^*$. The following Theorem is proved in the Supplementary Information; see Theorem 9.7 in Sect. 9.2.3.

#### Theorem 5.1

Suppose that $R_*>1$ and,

(i) $$\sqrt{m^{(v)}}\left( \alpha ^{(v)}_{\boldsymbol{n}}-\alpha _{\boldsymbol{n}}\right) \rightarrow 0\;$$ $$(\boldsymbol{n}\in \mathcal {N})$$,
(ii) there exists $\zeta >0$ such that $\mathbb {E}[(I^{(i)})^{2+\zeta }]<\infty$ $(i \in \mathcal {J})$ and
  5.10 $$\begin{aligned} \mathbb {E}\left[ \left( \sup _{t\ge 0}\left| R_j^{(\boldsymbol{n})}(t)\right| \right) ^{2+{\zeta }}\right] <\infty \qquad (\boldsymbol{n}\in \mathcal {N}, j=1,2,\dots ,p). \end{aligned}$$

Then as $v \rightarrow \infty$, conditional upon the event of a major outbreak occurring, the law of $${m^{(v)}}^{-\frac{1}{2}}\left( \begin{pmatrix} \boldsymbol{R}^{(v)} \\ A^{(v)} \end{pmatrix}-m^{(v)}\begin{pmatrix} \boldsymbol{r}(\tau ) \\ a(\tau ) \end{pmatrix}\right) $$ converges to the multivariate normal distribution $\textrm{N}(\boldsymbol{0}_{p+1}, \boldsymbol{H}\boldsymbol{C}(\tau )\boldsymbol{H}^{\top })$, where

5.11 $$\begin{aligned} \boldsymbol{H}=\begin{pmatrix} \boldsymbol{I}_p& \frac{1}{m_H-a^{\prime }(\tau )}\boldsymbol{r}^{\prime }(\tau ) \\ \boldsymbol{0}_p^T & \frac{m_H}{m_H-a^{\prime }(\tau )} \end{pmatrix}, \end{aligned}$$

with $$\boldsymbol{r}^{\prime }(\tau )=(r_1^{\prime }(\tau ),r_2^{\prime }(\tau ),\dots ,r_p^{\prime }(\tau ))^{\top }$$.

#### Remark 5.3

The condition (5.10) is often trivial to show. For example, if $R_j^{(\boldsymbol{n})}(t)$ is the number of individuals infected in the epidemic $\tilde{\mathcal {E}}_{\boldsymbol{n}}(t)$, then $0 \le R_j^{(\boldsymbol{n})}(t) \le \Vert \boldsymbol{n}\Vert$ for all *t*. If $R_j^{(\boldsymbol{n})}(t)$ is nonnegative and nondecreasing in *t* then $\sup _{t\ge 0}\left| R_j^{(\boldsymbol{n})}(t)\right| =R_j^{(\boldsymbol{n})}(\infty )$ and $\mathbb {E}[(R_j^{(\boldsymbol{n})}(\infty ))^{2+\zeta }]$ is often easily bounded as the whole household is infected in $\tilde{\mathcal {E}}_{\boldsymbol{n}}(\infty )$.

#### Remark 5.4

To use Theorem 5.1 in practice, we need to calculate, for each $\boldsymbol{n}\in \mathcal {N}$, the functions $a_{\boldsymbol{n}}(t), \boldsymbol{r}_{\boldsymbol{n}}(t)$ and $\boldsymbol{C}^{(\boldsymbol{n})}(t)$. The function $a_{\boldsymbol{n}}(t)$ can be calculated using the formula for $\tilde{\mu }_{\boldsymbol{n},j}(\Lambda ^L(\boldsymbol{n}),\boldsymbol{\pi })$ given in (10.2) in the Supplementary Information, Sect. 10. The other two functions depend on the choice of final outcome vector $\boldsymbol{R}'$. Many final outcome quantities are obtained by summing quantities over all individuals infected by the epidemic. The weighted severity is one such quantity, as is the total number of individuals infected of a given type. Calculation of $\boldsymbol{r}_{\boldsymbol{n}}(t)$ and $\boldsymbol{C}^{(\boldsymbol{n})}(t)$ for such final outcome quantities is considered in the Supplementary Information, Sect. 10. Note that if $R'$ is the indicator function of a household being infected, then $R^{(\boldsymbol{n})}(t) \sim \textrm{Bin}(1, 1-{\textrm{e}}^{-t \boldsymbol{n}\boldsymbol{\kappa }^{\top }})$, so $r^{(\boldsymbol{n})}(t)=1-{\textrm{e}}^{-t \boldsymbol{n}\boldsymbol{\kappa }^{\top }}$, and $A^{(\boldsymbol{n})}(t)= A^{(\boldsymbol{n})}(t) R^{(\boldsymbol{n})}(t)$, so ${\textrm{cov}}\left( A^{(\boldsymbol{n})}(t), R^{(\boldsymbol{n})}(t)\right) =a_{\boldsymbol{n}}(t){\textrm{e}}^{-t \boldsymbol{n}\boldsymbol{\kappa }^{\top }}$.

## Highly locally infectious epidemics

We illustrate Theorem 5.1 by considering the special case of a highly locally infectious disease, in which infection of a member of a household necessarily results in the whole household becoming infected (see, for example, Becker and Dietz (1995) and, in a multitype setting, Becker and Hall (1996)). For $v=1,2,\dots$, let $\boldsymbol{Z}^{(v)}=(Z^{(v)}_1, Z^{(v)}_2,\dots , Z^{(v)}_J)^{\top }$, where $Z^{(v)}_i$ is the total number of type-*i* individuals infected in the epidemic $\mathcal {E}^{(v)}$. We derive a central limit theorem for $\boldsymbol{Z}^{(v)}$, conditional upon a major outbreak, with an explicit expression for the (asymptotic) covariance matrix.

For $\boldsymbol{n}\in \mathcal {N}$, $t \ge 0$ and $i \in \mathcal {J}$, let $R^{(\boldsymbol{n})}_i(t)$ be the number of type-*i* individuals infected in the epidemic $\tilde{\mathcal {E}}_{\boldsymbol{n}}(\Lambda ^L(\boldsymbol{n}), {\textrm{e}}^{-t\boldsymbol{\kappa }})$, so with this choice of final outcome quantities, Theorem 5.1 yields a central limit theorem for $\boldsymbol{Z}^{(v)}$ conditional upon a major outbreak. The highly locally infectious assumption implies that, for $\boldsymbol{n}\in \mathcal {N}$,

$$ \left( R^{(\boldsymbol{n})}_1(t), R^{(\boldsymbol{n})}_2(t), \dots , R^{(\boldsymbol{n})}_J(t), A^{(\boldsymbol{n})}(t)\right) {\mathop {=}\limits ^{D}}D^{(\boldsymbol{n})}(t)\left( n_1,n_2, \dots , n_J, \sum _{j=1}^J \beta _j \sum _{k=1}^{n_j} I_k^{(j)}\right) , $$

where $D^{(\boldsymbol{n})}(t)\sim \textrm{Bin}(1, 1-{\textrm{e}}^{-t \boldsymbol{n}\boldsymbol{\kappa }^{\top }})$ and $I_k^{(j)} {\mathop {=}\limits ^{D}}I^{(j)}$ $(j \in \mathcal {J}, k=1,2,\dots , n_j)$ are independent random variables. For $\boldsymbol{n}\in \mathcal {N}$, let $b_1(\boldsymbol{n})= \sum _{k=1}^J \beta _kn_k\mu _I^{(k)}$ and $b_2(\boldsymbol{n})= \sum _{k=1}^J \beta _k^2 n_k\sigma ^2_{I,k}$, where $\sigma ^2_{I,k}={\textrm{var}}(I^{(k)})$. Elementary calculation yields that, for $\boldsymbol{n}\in \mathcal {N}$ and $t \ge 0$,

6.1 $$\begin{aligned} r_i^{(\boldsymbol{n})}(t)&=\mathbb {E}[R^{(\boldsymbol{n})}_i(t)]=n_i(1-{\textrm{e}}^{-t \boldsymbol{n}\boldsymbol{\kappa }^{\top }})\qquad (i \in \mathcal {J}),\nonumber \\ a^{(\boldsymbol{n})}(t)&=\mathbb {E}[A^{(\boldsymbol{n})}(t)]=b_1(\boldsymbol{n})(1-{\textrm{e}}^{-t \boldsymbol{n}\boldsymbol{\kappa }^{\top }}),\nonumber \\ {\textrm{cov}}(R^{(\boldsymbol{n})}_i(t),R^{(\boldsymbol{n})}_j(t))&=n_i n_j(1-{\textrm{e}}^{-t \boldsymbol{n}\boldsymbol{\kappa }^{\top }}){\textrm{e}}^{-t \boldsymbol{n}\boldsymbol{\kappa }^{\top }} \qquad (i,j \in \mathcal {J}), \end{aligned}$$

6.2 $$\begin{aligned} {\textrm{cov}}(R^{(\boldsymbol{n})}_i(t),A^{(\boldsymbol{n})}(t))&=n_i b_1(\boldsymbol{n})(1-{\textrm{e}}^{-t \boldsymbol{n}\boldsymbol{\kappa }^{\top }}){\textrm{e}}^{-t \boldsymbol{n}\boldsymbol{\kappa }^{\top }} \qquad (i \in \mathcal {J}), \end{aligned}$$

6.3 $$\begin{aligned} {\textrm{var}}(A^{(\boldsymbol{n})}(t))&=b_2(\boldsymbol{n})(1-{\textrm{e}}^{-t \boldsymbol{n}\boldsymbol{\kappa }^{\top }})+b_1(\boldsymbol{n})^2 (1-{\textrm{e}}^{-t \boldsymbol{n}\boldsymbol{\kappa }^{\top }}){\textrm{e}}^{-t \boldsymbol{n}\boldsymbol{\kappa }^{\top }}. \end{aligned}$$

Thus,

$$ a(t)=\sum _{\boldsymbol{n}\in \mathcal {N}} \alpha _{\boldsymbol{n}}a^{(\boldsymbol{n})}(t)=\sum _{\boldsymbol{n}\in \mathcal {N}}\alpha _{\boldsymbol{n}}b_1(\boldsymbol{n})(1-{\textrm{e}}^{-t \boldsymbol{n}\boldsymbol{\kappa }^{\top }})\qquad (t \ge 0) $$

and

$$ R_*=m_H^{-1}a^{\prime }(0)=m_H^{-1}\sum _{\boldsymbol{n}\in \mathcal {N}}\alpha _{\boldsymbol{n}}b_1(\boldsymbol{n}) \boldsymbol{n}\boldsymbol{\kappa }^{\top }. $$

Suppose that $R_*>1$ and let $\tau$ be the unique solution in $(0, \infty )$ of $t=m_H^{-1}a(t)$. For $t \ge 0$ and $i \in \mathcal {J}$, let

$$ r_i(t)=\sum _{\boldsymbol{n}\in \mathcal {N}} \alpha _{\boldsymbol{n}}r_i^{(\boldsymbol{n})}(t)=\sum _{\boldsymbol{n}\in \mathcal {N}} \alpha _{\boldsymbol{n}}n_i(1-{\textrm{e}}^{-t \boldsymbol{n}\boldsymbol{\kappa }^{\top }}), $$

so

$$ \boldsymbol{r}(t)=\sum _{\boldsymbol{n}\in \mathcal {N}} \alpha _{\boldsymbol{n}}(1-{\textrm{e}}^{-t \boldsymbol{n}\boldsymbol{\kappa }^{\top }})\tilde{\boldsymbol{n}}, $$

where $\tilde{\boldsymbol{n}}=(n_1, n_2,\dots , n_J, b_1(\boldsymbol{n}))^{\top }$. Also, $\boldsymbol{C}(\tau )=\sum _{\boldsymbol{n}\in \mathcal {N}} \alpha _{\boldsymbol{n}}\boldsymbol{C}^{(\boldsymbol{n})}(\tau )$, where using (6.1)-(6.3),

$$ \boldsymbol{C}^{(\boldsymbol{n})}(\tau )=(1-{\textrm{e}}^{-\tau \boldsymbol{n}\boldsymbol{\kappa }^{\top }}){\textrm{e}}^{-\tau \boldsymbol{n}\boldsymbol{\kappa }^{\top }}\tilde{\boldsymbol{n}}\tilde{\boldsymbol{n}}^{\top }+(1-{\textrm{e}}^{-\tau \boldsymbol{n}\boldsymbol{\kappa }^{\top }})b_2(\boldsymbol{n})\tilde{\boldsymbol{e}}\tilde{\boldsymbol{e}}^{\top }, $$

and $\tilde{\boldsymbol{e}}$ denotes the $(J+1)\times 1$ vector $(0,0, \dots , 0,1)^{\top }$. Let $\boldsymbol{z}=(z_1,z_2,\dots ,z_J)^{\top }$, where $z_i=r_i(\tau )$ $(i \in \mathcal {J})$. To connect with Sect. 5.1, note that $z_i=\gamma _i \tilde{z}_i$.

Let $\boldsymbol{H}$ be given by (5.11). Noting that, for $\boldsymbol{n}\in \mathcal {N}$,

$$\begin{aligned}&\boldsymbol{H}\tilde{\boldsymbol{n}}= \begin{pmatrix} \boldsymbol{n}^{\top }+b_1(\boldsymbol{n})(m_H-a^{\prime }(\tau ))^{-1}\boldsymbol{r}^{\prime }(\tau ) m_H(m_H-a^{\prime }(\tau ))^{-1}b_1(\boldsymbol{n}) \end{pmatrix} \quad {\text {and}}\\&\quad \boldsymbol{H}\tilde{\boldsymbol{e}}=(m_H-a^{\prime }(\tau ))^{-1} \begin{pmatrix}\boldsymbol{r}^{\prime }(\tau ) m_H \end{pmatrix}, \end{aligned}$$

it follows using Theorem 5.1 that as $v \rightarrow \infty$, conditional upon a major outbreak,

6.4 $$\begin{aligned} {m^{(v)}}^{-\frac{1}{2}}\left( \boldsymbol{Z}^{(v)}-m^{(v)}\boldsymbol{z}\right) {\mathop {\longrightarrow }\limits ^{{\textrm{D}}}}\textrm{N}(\boldsymbol{0}_{J}, \boldsymbol{\Sigma }), \end{aligned}$$

where

6.5 $$\begin{aligned} \boldsymbol{\Sigma }=\sum _{\boldsymbol{n}\in \mathcal {N}} \alpha _{\boldsymbol{n}}(1-{\textrm{e}}^{-\tau \boldsymbol{n}\boldsymbol{\kappa }^{\top }})(\boldsymbol{S}_1^{(\boldsymbol{n})}+{\textrm{e}}^{-\tau \boldsymbol{n}\boldsymbol{\kappa }^{\top }}\boldsymbol{S}_2^{(\boldsymbol{n})}), \end{aligned}$$

with

$$\begin{aligned} \boldsymbol{S}_1^{(\boldsymbol{n})}&=\frac{b_2(\boldsymbol{n})}{({m_H-a^{\prime }(\tau )})^2}\boldsymbol{r}^{\prime }(\tau )\boldsymbol{r}^{\prime }(\tau )^{\top },\\ \boldsymbol{S}_2^{(\boldsymbol{n})}&=\boldsymbol{n}^{\top }\boldsymbol{n}+\frac{b_1(\boldsymbol{n})}{m_H-a^{\prime }(\tau )}(\boldsymbol{n}^{\top }\boldsymbol{r}^{\prime }(\tau )^{\top }+\boldsymbol{r}^{\prime }(\tau )\boldsymbol{n}) +\left( \frac{b_1(\boldsymbol{n})}{m_H-a^{\prime }(\tau )}\right) ^2\boldsymbol{r}^{\prime }(\tau )\boldsymbol{r}^{\prime }(\tau )^{\top }. \end{aligned}$$

## Standard SIR multitype epidemics

If all households have size 1, the model reduces to a special case of the standard SIR multitype epidemic model (Andersson and Britton 2000, Chapter 6) in which mixing is proportionate. We use notation and results from Sect. 6. There are *J* distinct household categories and $\mathcal {N}=\{\boldsymbol{e}_1, \boldsymbol{e}_2,\dots , \boldsymbol{e}_J\}$, where, for $i \in \mathcal {J}$, $\boldsymbol{e}_i$ denotes the unit vector of length *J*, in which the $i^\textrm{th}$ element is 1 and all other elements are 0. For $k \in \mathcal {J}$, $\alpha _{\boldsymbol{e}_k}=\gamma _k$, $b_1(\boldsymbol{e}_k)=\beta _k\mu _I^{(k)}$, $b_2(\boldsymbol{e}_k)=\beta _k^2\sigma _{I,k}^2$ and $r_i^{(\boldsymbol{e}_k)}(t)=\delta _{ki}(1-{\textrm{e}}^{-\kappa _i t})$ $(i \in \mathcal {J})$. Hence, $r_i(t)=\gamma _i(1-{\textrm{e}}^{-\kappa _i t})$ $(i \in \mathcal {J})$ and $a(t)=\sum _{k=1}^J \gamma _k \beta _k \mu _I^{(k)} (1-{\textrm{e}}^{-\kappa _k t})$. Noting that $m_H=1$, it follows that $R_0$ $(=R_*) =a^{\prime }(0)=\sum _{k=1}^J \gamma _k \beta _k \kappa _k \mu _I^{(k)}$.

Suppose that $R_0>1$ and let $\tau$ be the unique solution in $(0, \infty )$ of $t=a(t)$. For $i \in \mathcal {J}$, let $z_i=r_i(\tau )=\gamma _i(1-{\textrm{e}}^{-\kappa _i \tau })$ and $\rho _i=\gamma _i-z_i$. Then, $r_i^{\prime }(\tau )=\kappa _i \rho _i$ $(i \in \mathcal {J})$ and $a^{\prime }(\tau )=\sum _{k=1}^J\kappa _k \beta _k \rho _k \mu _I^{(k)}$. For $k \in \mathcal {J}$, note that $\alpha _{\boldsymbol{e}_k}(1-{\textrm{e}}^{-\boldsymbol{e}_k \boldsymbol{\kappa }^{\top }\tau })=z_k$, $\alpha _{\boldsymbol{e}_k}(1-{\textrm{e}}^{-\boldsymbol{e}_k \boldsymbol{\kappa }^{\top }\tau }){\textrm{e}}^{-\boldsymbol{e}_k \boldsymbol{\kappa }^{\top }\tau }=\gamma _k^{-1}z_k \rho _k$ and $[\boldsymbol{e}_k^{\top }\boldsymbol{r}^{\prime }(\tau )^{\top }+\boldsymbol{r}^{\prime }(\tau )\boldsymbol{e}_k]_{ij}=\delta _{ik}\kappa _j\rho _j+\delta _{jk}\kappa _i\rho _i$ $(i,j, \in \mathcal {J})$. It then follows from (6.4) and (6.5), and a little algebra, that as $v \rightarrow \infty$, conditional upon a major outbreak,

$$ {m^{(v)}}^{-\frac{1}{2}}\left( \boldsymbol{Z}^{(v)}-m^{(v)}\boldsymbol{z}\right) \,|\, G^{(v)}{\mathop {\longrightarrow }\limits ^{{\textrm{D}}}}\textrm{N}(\boldsymbol{0}_{J}, \boldsymbol{\Sigma }), $$

where $\boldsymbol{z}=(z_1,z_2, \dots , z_J)^{\top }$ and $\Sigma =[\sigma _{ij}]$ with, for $i, j \in \mathcal {J}$,

7.1 $$\begin{aligned} \sigma _{ij}&=\delta _{ij}\frac{z_i \rho _i}{\gamma _i}+\frac{\rho _i\rho _j}{1-\sum _{k=1}^J \kappa _k \beta _k \rho _k \mu _I^{(k)}}\left( \frac{\beta _i \mu _I^{(i)} z_i \kappa _j}{\gamma _i}+\frac{\beta _j \mu _I^{(j)} z_j \kappa _i}{\gamma _j}\right) \nonumber \\&\qquad \qquad +\frac{\kappa _i\rho _i\kappa _j\rho _j}{\left( 1-\sum _{k=1}^J \kappa _k \beta _k \rho _k \mu _I^{(k)}\right) ^2}\sum _{k=1}^J \beta _k^2 z_k \left[ \sigma _{I,k}^2+\frac{\rho _k(\mu _I^{(k)})^2}{\gamma _k}\right] . \end{aligned}$$

Lengthy algebra shows that (7.1) is consistent with the asymptotic covariance matrix given in Andersson and Britton (2000), Sect. 6.2, which is obtained from Ball and Clancy (1993), equations (4.9), (4.10) and Sect. 4.1. Note that Andersson and Britton (2000) and Ball and Clancy (1993) consider the asymptotic distribution of $\tilde{\boldsymbol{Z}}^{(v)}=(\tilde{Z}^{(v)}_1, \tilde{Z}^{(v)}_2, \dots , \tilde{Z}^{(v)}_J)$, where, in our notation, $\tilde{Z}^{(v)}_i=(Z_i^{(v)}-N_i^{(v)} \tilde{z}_i)/\sqrt{N_i^{(v)}}$ $(i \in \mathcal {J})$. The asymptotic covariance matrix in Andersson and Britton (2000) and Ball and Clancy (1993) involves the inverse of a $J \times J$ matrix, which can be found explicitly when mixing is proportionate.

## Concluding comments

We have shown that the assumption of proportionate global mixing leads to considerable simplification in the calculation of reproduction numbers and asymptotic properties for stochastic multitype SIR epidemics among a population of households. Our original motivation for investigating the multitype households model with proportionate global mixing was to study disease-induced herd immunity for a model with both household structure and activity levels. As mentioned in the introduction, Britton et al. (2020) show that heterogeneities in activity can lower appreciably the disease-induced herd immunity level. Household structure generally has the opposite effect on disease-induced herd immunity (Ball et al. 2023). In a manuscript in preparation, we study disease-induced herd immunity for a model which includes both features, where having a single-variable equation that determines the final size of a major outbreak is crucial for obtaining analytical results.

For ease of exposition, we have not presented the results in full generality. For example, we can allow the overall rate that an individual makes global contacts to depend also on household category, i.e. replace the proportionate mixing assumption $\lambda ^G_{ij}=\beta _i\kappa _j$ by $\lambda ^G_{ij}(\boldsymbol{n})=\beta _i(\boldsymbol{n})\kappa _j$ $(i, j \in \mathcal {J})$. Under this generalisation, the approximating branching processes $\mathcal {B}$ and $\mathcal {B}_B$ are still effectively single-type and the key processes underlying the embedding construction in the proof of the central limit theorem (Supplementary Information, Sect. 9.2.1) still have a one-dimensional index set, so all the results and proofs continue to hold with minor modification.

As noted in Sect. 2, our results can be extended to models with nonconstant infectivity profiles. Suppose that, for $i \in \mathcal {J}$, a typical type-*i* infective has infectivity profile, $\mathcal {I}^{(i)}(t)$ $(t \ge 0)$, a nonnegative stochastic process describing the infectiousness of an infective as a function of the time *t* since they were infected; cf.  Goldstein et al. (2009). Conditional upon their infectivity profile, a type-*i* infective, $i_*$ say, residing in a category-$\boldsymbol{n}$ household, makes local contacts with any given individual in their household at the points of a Poisson process having rate $\lambda ^L_{ij}(\boldsymbol{n})\mathcal {I}^{(i)}(t)$ and global contacts with any given type-*j* individual in the population at the points of a Poisson process having rate $N^{-1}\lambda ^G_{ij}\mathcal {I}^{(i)}(t)$, where *t* is the time since $i_*$ was infected. The infectivity profiles of different infectives are mutually independent, with those of the same type being identically distributed. As before, we assume that $\lambda ^G_{ij}=\beta _i\kappa _j$ $(i,j \in \mathcal {J})$. All the results of the paper can be extended to this model. With every result apart from the early exponential growth rate *r*, we simply let $I^{(i)}=\int _0^{\infty } \mathcal {I}^{(i)}(t)\,\textrm{d}t$ $(i \in \mathcal {J})$ and proceed as before. The early exponential growth rate *r* is still given by the unique positive solution of the Lotka-Euler equation (4.9), with $\beta (t)$ given by (4.10), but note that $\beta _{\boldsymbol{n}, i}(t)$ depends on $\mathcal {I}^{(j)}(u)$ $(0 \le u \le t, j \in \mathcal {J})$ and is typically hard to calculate. Again these results can be extended to the case when $\lambda ^G_{ij}$ depends on household category, as above. They can also be extended, at least in principle, to the case where an infective has different, possibly dependent, infectivity profiles for local and global infections; by using results in Ball (2019), Sect. 4.4.4, for general final state random variables defined on multitype collective Reed-Frost epidemics (for results other than *r*).

The multitype households model is very general and by suitable choice of the type space includes, for example, models with more than two levels of mixing, such as a population of villages, each partitioned into households (cf.  Britton et al. (2007) and Ouboter et al. (2016)).

The central limit theorem for final state random variables is also very general and is applicable to more complex SIR models. For example, in the carrier-borne model of Downton (1968), infected susceptibles are detected immediately independently with a specified probability, so in a single-type setting the effective infectious period distribution is a mixture of a point mass at zero and a strictly positive random variable *I*. This can be extended to the present multitype households model setting, with the detection probability possibly depending on both an individual’s type *i* and the category $\boldsymbol{n}$ of their household. Suitably defined final state random variables can be used to obtain a multivariate central limit theorem for the numbers infected and the numbers detected immediately of the *J* types of individuals. The model can be extended further so that infected susceptibles are split into more than just two types of infectives; cf. Picard and Lefèvre (1990). Another extension to the standard SIR model considered in Picard and Lefèvre (1990) concerns models in which infectives pass through stages of infection, possibly having different levels of infectiousness. Again this can be extended to the multitype households model setting and, under suitable conditions, Theorem 5.1 can be used to obtain, for example, a multivariate central limit theorem for the total severity in the different stages of infection for the *J* types of individuals. See Ball (2019) for further detail about such extensions in the context of exact results for the final outcome of SIR epidemics in populations without household structure.

## Supplementary Information

Below is the link to the electronic supplementary material.

Supplementary file 1 (pdf 483 KB)

## Declarations

### Data availability

No new data were created during this study.
