# Supplementary material for: Multitype SIR epidemics among a population partitioned into households with proportionate global mixing
Source: J Math Biol. 2026 Apr 26;92(5):77. doi: 10.1007/s00285-026-02359-5 (PMC13111495; doi:10.1007/s00285-026-02359-5)
Supplement: Supplementary file 1 — (pdf 483 KB) [file 285_2026_2359_MOESM1_ESM.pdf]

Supplementary Information: Multitype SIR epidemics among a population partitioned into households with proportionate global mixing

Frank Ball[1*] and Liam Critcher[1,2]

[1]School of Mathematical Sciences, University of Nottingham, University Park, Nottingham, NG7 2RD, UK.
[2]Department of Mathematics, University of Manchester, Alan Turing Building, Oxford Road, Manchester, M13 9PL, State, UK.

*Corresponding author(s). E-mail(s): frank.ball@nottingham.ac.uk;
Contributing authors: liam.critcher@manchester.ac.uk;

In the Supplementary Information we provide a rigorous justification of the results in the main text, including a proof of the central limit theorem for the final outcome of a major outbreak. We also outline calculation of properties of some single-population multitype SIR epidemics, required to implement the asymptotic results. The section counter continues from the main text, with the first section being Section 9. All references to sections, theorems and equations etc. with a section number less than 9 are to the main text.

9 Rigorous results and proofs

Recall the epidemic model $\mathcal{E}$ defined in Section 2. In order to state and prove rigorous results we consider a sequence of such epidemics, $\mathcal{E}^{(v)}$ $(v = 1, 2, \dots)$, with the epidemic $\mathcal{E}^{(v)}$ being among a population of $m^{(v)}$ households comprising $m_{\boldsymbol{n}}^{(v)}$ households of category $\boldsymbol{n}$ $(\boldsymbol{n} \in \mathcal{N})$. Let $\alpha_{\boldsymbol{n}}^{(v)} = m_{\boldsymbol{n}}^{(v)}/m^{(v)}$ $(\boldsymbol{n} \in \mathcal{N})$. We assume that $\lim_{v\to\infty} m^{(v)} = \infty$ and $\lim_{v\to\infty} \alpha_{\boldsymbol{n}}^{(v)} = \alpha_{\boldsymbol{n}}$ $(\boldsymbol{n} \in \mathcal{N})$. We assume also that the configuration of initial infectives (i.e. their number and distribution among the household categories) is the same for all v. Further assumptions are stated as required. Notation concerning household structure used in earlier sections indexed by v means the same quantity for the epidemic $\mathcal{E}^{(v)}$, with un-indexed notation being the limiting value as $v \to \infty$. For example, $\gamma_i^{(v)} = N_i^{(v)}/N^{(v)}$ is the fraction of the population that are of

type i in $\mathcal{E}^{(v)}$ and $\gamma_i = \lim_{n\to\infty} \gamma_i^{(v)}$; note that this limit necessarily exists. We assume that $\min_{i\in\mathcal{J}} \gamma_i > 0$.

9.1 Branching process approximation

Let $\mathcal{B}^{(v)}$ denote the branching process defined in Section 4.1 but using the household structure of $\mathcal{E}^{(v)}$ and now let $\mathcal{B}$ be the branching process using the limiting household structure. Let $Z^{(v)}$ and Z be the total progeny of $\mathcal{B}^{(v)}$ and $\mathcal{B}$, respectively. (We assume throughout that total progeny includes the ancestor(s).) Let $Z_E^{(v)}$ be the total number of households infected in $\mathcal{E}^{(v)}$, including the initially infected households. We denote the occurrence of a major outbreak by the event $G^{(v)} = \{Z_E^{(v)} \geqslant \log m^{(v)}\}$. Let p_{ext} denote the extinction probability of $\mathcal{B}$.

Theorem 9.1 *We have* $\lim_{v\to\infty} \mathbb{P}(G^{(v)}) = 1 - p_{\text{ext}}$.

Proof For ease of presentation, we assume that there is one initial infective in $\mathcal{E}^{(v)}$, who is chosen by first sampling its type j from $P_i^{(v)}$ $(i \in \mathcal{J})$ and choosing an individual uniformly at random from the $N_j^{(v)}$ type-j individuals in the population. This implies that in $\mathcal{B}^{(v)}$ the ancestor has the same offspring distribution as all subsequent individuals. A realisation of $\mathcal{B}^{(v)}$ can be obtained using independent copies of the augmented single-household epidemics $\hat{\mathcal{E}}_{\boldsymbol{n},i}(\Lambda^L(\boldsymbol{n}), \boldsymbol{\beta})$ $(i \in \mathcal{J}, \boldsymbol{n} \in \mathcal{N}_i)$, analogously to $\mathcal{B}$ in Section 4.1, where for each global contact in $\mathcal{B}^{(v)}$, the type is distributed according to $P_i^{(v)}$ $(i \in \mathcal{J})$ and given its type is j, the household category is obtained by that of an individual chosen independently and uniformly from the $N_j^{(v)}$ type-j individuals in the population underlying $\mathcal{E}^{(v)}$.

A realisation of $\mathcal{E}^{(v)}$ can be defined in a similar fashion. However, if a global contact (of type i say) is with a household that has been infected previously in the epidemic then the ensuing local epidemic and the number of global contacts that emanate from it is not distributed as $\hat{\mathcal{E}}_{\boldsymbol{n},i}(\Lambda^L(\boldsymbol{n}), \boldsymbol{\beta})$, so the construction of the realisation of $\mathcal{E}^{(v)}$ then needs modifying accordingly. Let $K^{(v)}$ be the number of global contacts made in $\mathcal{B}^{(v)}$ when a previously-used household is sampled for a global contact for the first time, where $K^{(v)} = \infty$ if all global contacts in the construction of $\mathcal{B}^{(v)}$ are with previously unused households. The realisations of $\mathcal{E}^{(v)}$ and $\mathcal{B}^{(v)}$ can be coupled so that they coincide while fewer than $K^{(v)}$ households have been infected in $\mathcal{E}^{(v)}$. Now

$$\mathbb{P}\left(Z_E^{(v)} < \log m^{(v)}\right) = \mathbb{P}\left(Z_E^{(v)} < \log m^{(v)}, K^{(v)} \leqslant \log m^{(v)}\right) + \mathbb{P}\left(Z_E^{(v)} < \log m^{(v)}, K^{(v)} > \log m^{(v)}\right)$$

and

$$\mathbb{P}\left(Z^{(v)} < \log m^{(v)}\right) = \mathbb{P}\left(Z^{(v)} < \log m^{(v)}, K^{(v)} \leqslant \log m^{(v)}\right) + \mathbb{P}\left(Z^{(v)} < \log m^{(v)}, K^{(v)} > \log m^{(v)}\right).$$

Under the above coupling,

$$\mathbb{P}\left(Z_E^{(v)} < \log m^{(v)}, K^{(v)} > \log m^{(v)}\right) = \mathbb{P}\left(Z^{(v)} < \log m^{(v)}, K^{(v)} > \log m^{(v)}\right),$$

so

$$\begin{aligned}&\left|\mathbb{P}\left(Z_E^{(v)} < \log m^{(v)}\right) - \mathbb{P}\left(Z^{(v)} < \log m^{(v)}\right)\right| \\ &\qquad = \left|\mathbb{P}\left(Z_E^{(v)} < \log m^{(v)}, K^{(v)} \leqslant \log m^{(v)}\right) - \mathbb{P}\left(Z^{(v)} < \log m^{(v)}, K^{(v)} \leqslant \log m^{(v)}\right)\right| \\ &\qquad \leqslant \mathbb{P}\left(K^{(v)} \leqslant \lfloor \log m^{(v)} \rfloor\right),\end{aligned}$$

where $\lfloor \log m^{(v)} \rfloor$ is the greatest integer $\leqslant \log m^{(v)}$. The probability that a global contact is with any given household is bounded above by

$$\max_{i\in\mathcal{J}} \max_{\boldsymbol{n}\in\mathcal{N}} \frac{n_i}{N_i^{(v)}} \leqslant \frac{n_{\max}}{N^{(v)} \min_{i\in\mathcal{J}} \gamma_i^{(v)}}. \tag{9.1}$$

Thus, noting the connection with the birthday problem,

$$\mathbb{P}\left(K^{(v)} \leqslant \lfloor \log m^{(v)} \rfloor\right) \leqslant \binom{\lfloor \log m^{(v)} \rfloor}{2} \frac{n_{\max}}{N^{(v)} \min_{i\in\mathcal{J}} \gamma_i^{(v)}} \to 0 \qquad \text{as } v \to \infty, \tag{9.2}$$

since $N^{(v)} \geqslant m^{(v)}$, $\lim_{v\to\infty} m^{(v)} = \infty$ and $\lim_{v\to\infty} \min_{i\in\mathcal{J}} \gamma_i^{(v)} = \min_{i\in\mathcal{J}} \gamma_i > 0$. Hence,

$$\lim_{v\to\infty} \left|\mathbb{P}\left(Z_E^{(v)} < \log m^{(v)}\right) - \mathbb{P}\left(Z^{(v)} < \log m^{(v)}\right)\right| = 0.$$

To complete the proof, we show that $\mathbb{P}\left(Z^{(v)} < \log m^{(v)}\right) \to p_{\text{ext}}$ as $v \to \infty$. Let $p_{\text{ext}}^{(v)}$ be the extinction probability of $\mathcal{B}^{(v)}$ and note that $p_{\text{ext}}^{(v)} \to p_{\text{ext}}$ as $v \to \infty$ by Britton et al. [5], Lemma 4.1, since the offspring distribution of $\mathcal{B}^{(v)}$ converges to that of $\mathcal{B}$. Hence,

$$\limsup_{v\to\infty} \mathbb{P}\left(Z^{(v)} < \log m^{(v)}\right) \leqslant \limsup_{v\to\infty} \mathbb{P}\left(Z^{(v)} < \infty\right) = \limsup_{v\to\infty} p_{\text{ext}}^{(v)} = p_{\text{ext}}. \tag{9.3}$$

Note also that for any $k \in \mathbb{N}$, we have $\mathbb{P}\left(Z^{(v)} < k\right) \to \mathbb{P}(Z < k)$ as $v \to \infty$, since there are a finite number of paths of $\mathcal{B}^{(v)}$ yielding $Z^{(v)} < k$. Hence, for $k \in \mathbb{N}$,

$$\liminf_{v\to\infty} \mathbb{P}\left(Z^{(v)} < \log m^{(v)}\right) \geqslant \liminf_{v\to\infty} \mathbb{P}\left(Z^{(v)} < k\right) = \mathbb{P}(Z < k). \tag{9.4}$$

Now $\mathbb{P}(Z < k) \uparrow p_{\text{ext}}$ as $k \uparrow \infty$, so letting $k \uparrow \infty$ in (9.4) yields

$$\liminf_{v\to\infty} \mathbb{P}\left(Z^{(v)} < \log m^{(v)}\right) \geqslant p_{\text{ext}},$$

which together with (9.3) gives $\mathbb{P}(Z^{(v)} < \log m^{(v)}) \to p_{\text{ext}}$ as $v \to \infty$, as required. □

Remark 9.1 *Note from* (9.2) *that Theorem 9.1 holds if the definition of a major outbreak is replaced by $G^{(v)} = \{Z_E^{(v)} \geqslant f(m^{(v)})\}$, where $f : \mathbb{N} \to \mathbb{R}_+$ is any nondecreasing function satisfying $f(m) \to \infty$ and $m^{-1} f(m)^2 \to 0$ as $m \to \infty$. In fact, Theorem 9.1 holds under stricter definitions of a major outbreak, see Remark 9.5 at the end of Section 9.2.2, though the current proof breaks down.*

Remark 9.2 *A similar argument using* (9.2) *shows that other properties of the epidemic $\mathcal{E}^{(v)}$, for example the total number infected of each type, converge in distribution to corresponding properties of $\mathcal{B}$ as $v \to \infty$. Depending on the property, these asymptotic distributions may be defective if $R_* > 1$.*

9.2 Final outcome of major outbreak

We prove a law of large numbers and a multivariate central limit theorem for the final outcome of a major outbreak. The key tool is an embedding construction of the final outcome of $\mathcal{E}^{(v)}$, which in the special case of proportionate global mixing has a one-dimensional index set (rather than J-dimensional in the general case). This leads to simplifications both in the proofs and in computing the asymptotic mean vector and variance matrix. The embedding construction is described in Section 9.2.1, with the law of large numbers and multivariate central limit theorem being proved in Sections 9.2.2 and 9.2.3, respectively.

9.2.1 Embedding construction.

Consider the epidemic model $\mathcal{E}$ described in Section 2. An alternative but equivalent way of treating global infection in that model is via a Sellke [10] construction. Each susceptible individual in the population is assigned an independent exponential random variable, having rate κ_i for a type-i individual, giving the critical amount of global infection they need to be exposed to before they become infected. During the epidemic, each susceptible accumulates exposure to global infection from each infective, at rate β_j/N for a type-j infective, and provided they have not previously been infected locally, a susceptible is infected globally if and when their total exposure to global infection reaches their critical amount. This way of treating global infection is faithful to the original model description in Section 2, owing to the lack-of-memory property of the exponential distribution. Suppose at a given time, t say, there are a_i type-i infectives ($i \in \mathcal{J}$) and consider a type-j susceptible, having critical exposure to global infection L_j, that has already been exposed to b units of global infection. The probability that this susceptible is infected in the time interval $(t, t + \Delta t)$ is

$$\mathbb{P}\left(L_j \in \left(b, b + N^{-1} \sum_{i \in \mathcal{J}} a_i \beta_i \Delta t\right) | L_j > b\right) = \kappa_j N^{-1} \sum_{i \in \mathcal{J}} a_i \beta_i \Delta t + o(\Delta t),$$

as in the formulation in Section 2.

To analyse the asymptotic distribution of the final outcome of $\mathcal{E}^{(v)}$, we use the embedding approach of Scalia-Tomba [8, 9]. The construction here is closely related to those in Ball et al. [4] and Ball and Lyne [3]. Consider first a single category-$\boldsymbol{n}$ household and define a process $\tilde{\mathcal{E}}_{\boldsymbol{n}} = \{\tilde{\mathcal{E}}_{\boldsymbol{n}}(t) : t \geqslant 0\}$ as follows. Note that here and in the sequel t refers to global infectious pressure and not to time. For $i \in \mathcal{J}$, label the type-i individuals in the household (i, j) $(j = 1, 2, \ldots, n_i)$. Let $\mathcal{G}_{\boldsymbol{n}}(\Lambda^L(\boldsymbol{n}))$ be a realisation of the random directed graph defined in Section 3.3. For $i \in \mathcal{J}$ and $j = 1, 2, \ldots, n_i$, let I_{ij} be the infectious period of individual (i, j) if they were to become infected. Let L_{ij} $(i \in \mathcal{J}, j = 1, 2, \ldots, n_i)$ be an independent collection of independent exponential random variables, where L_{ij} has rate κ_i. For each $t \geqslant 0$, we construct an epidemic $\tilde{\mathcal{E}}_{\boldsymbol{n}}(t)$ as follows. We determine first who is infected globally (i.e. externally); specifically, individual (i, j) is infected globally if and only if $L_{ij} \leqslant t$. We then use the random directed graph $\mathcal{G}_{\boldsymbol{n}}(\Lambda^L(\boldsymbol{n}))$ to determine who is infected locally; these local infections take place instantaneously with respect to t.

For $t \geqslant 0$ and $i \in \mathcal{J}$, let $\tilde{A}_i^{(\boldsymbol{n})}(t)$ denote the sum of the infectious periods of all type-i individuals infected in $\tilde{\mathcal{E}}_{\boldsymbol{n}}(t)$, and let $A^{(\boldsymbol{n})}(t) = \sum_{i=1}^J \beta_i \tilde{A}_i^{(\boldsymbol{n})}(t)$ be the weighted severity of $\tilde{\mathcal{E}}_{\boldsymbol{n}}(t)$. Note that a single realisation of $\mathcal{G}_{\boldsymbol{n}}(\Lambda^L(\boldsymbol{n}))$ and (L_{ij}, I_{ij}) $(i \in \mathcal{J}, j = 1, 2, \dots, n_i)$ is used to define $\tilde{\mathcal{E}}_{\boldsymbol{n}}(t)$, and hence also $A^{(\boldsymbol{n})}(t)$, for all $t \geqslant 0$, and that by construction $A^{(\boldsymbol{n})}(t)$ is piecewise constant and nondecreasing in t. Observe that $\tilde{\mathcal{E}}_{\boldsymbol{n}}(t)$ is a realisation of the epidemic $\tilde{\mathcal{E}}_{\boldsymbol{n}}(\Lambda^L(\boldsymbol{n}), \mathrm{e}^{-t\boldsymbol{\kappa}})$ defined in Section 3.2, where $\mathrm{e}^{-t\boldsymbol{\kappa}} = (\mathrm{e}^{-t\kappa_1}, \mathrm{e}^{-t\kappa_2}, \dots, \mathrm{e}^{-t\kappa_J})$. Thus, $\mathbb{E}\left[\tilde{A}_i^{(\boldsymbol{n})}(t)\right] = \tilde{\mu}_{\boldsymbol{n},i}(\Lambda^L(\boldsymbol{n}), \mathrm{e}^{-t\boldsymbol{\kappa}})\mu_I^{(i)}$ $(i \in \mathcal{J})$.

Let $\tilde{\mathcal{E}}_{\boldsymbol{n},k}$ $(\boldsymbol{n} \in \mathcal{N}, k = 1, 2, \dots)$ be independent, with $\tilde{\mathcal{E}}_{\boldsymbol{n},k} = \{\tilde{\mathcal{E}}_{\boldsymbol{n},k} : t \geqslant 0\}$ distributed as $\tilde{\mathcal{E}}_{\boldsymbol{n}}$, and for $t \geqslant 0$, let $A^{(\boldsymbol{n},k)}(t)$ be the weighted severity of $\tilde{\mathcal{E}}_{\boldsymbol{n},k}(t)$. For $v = 1, 2, \dots,$ we use $\tilde{\mathcal{E}}_{\boldsymbol{n},k}$ $(\boldsymbol{n} \in \mathcal{N}, k = 1, 2, \dots, m_{\boldsymbol{n}}^{(v)})$ to construct a process $\hat{\mathcal{E}}^{(v)} = \{\hat{\mathcal{E}}^{(v)}(t) : t \geqslant 0\}$ among a population comprising $m_{\boldsymbol{n}}^{(v)}$ households of category $\boldsymbol{n}$ $(\boldsymbol{n} \in \mathcal{N})$, i.e. among a population having the same household structure as $\mathcal{E}^{(v)}$. For $t \geqslant 0$, the epidemic $\hat{\mathcal{E}}^{(v)}(t)$ assumes that each individual in the population is exposed to t units of global infectious pressure, in the above sense, and that the epidemics in the $m^{(v)} = \sum_{\boldsymbol{n} \in \mathcal{N}} m_{\boldsymbol{n}}^{(v)}$ households behave independently, with the epidemic in the household $(\boldsymbol{n}, k)$ being given by $\tilde{\mathcal{E}}_{\boldsymbol{n},k}(t)$. Let

$$A_{\bullet}^{(v)}(t) = \sum_{\boldsymbol{n} \in \mathcal{N}} \sum_{k=1}^{m_{\boldsymbol{n}}^{(v)}} A^{(\boldsymbol{n},k)}(t) \qquad (t \geqslant 0). \tag{9.5}$$

This defines a process $A_{\bullet}^{(v)} = \{A_{\bullet}^{(v)}(t) : t \geqslant 0\}$, which is nondecreasing by construction.

The realisation of $\hat{\mathcal{E}}^{(v)}$ can be used to construct the final outcome of an epidemic $\tilde{\mathcal{E}}^{(v)}$, which is closely related to the epidemic $\mathcal{E}^{(v)}$, as follows. The epidemic $\tilde{\mathcal{E}}^{(v)}$ is initiated by exposing the population to $T_0^{(v)}$ units of global infectious pressure, so each individual is exposed to $\bar{T}_0^{(v)} = T_0^{(v)}/N^{(v)}$ units. Individuals with tolerance $L_{ij} \leqslant \bar{T}_0^{(v)}$ will be infected by this initial global pressure. Such individuals will then trigger local epidemics in their households, which in total will give rise to a further $A_{\bullet}^{(v)}(\bar{T}_0^{(v)})$ units of global infectious pressure. Hence, the population is now exposed to a total of $T_1^{(v)} = T_0^{(v)} + A_{\bullet}^{(v)}(\bar{T}_0^{(v)})$ units of global infectious pressure. The process can be continued in the obvious fashion and stops when there is an iterative step which yields no additional global infectious pressure. Note that this happens in a finite number of steps as the population is finite. For $k = 0, 1, \dots,$ let $T_{k+1}^{(v)} = T_0^{(v)} + A_{\bullet}^{(v)}(\bar{T}_k^{(v)})$, where $\bar{T}_k^{(v)} = T_k^{(v)}/N^{(v)}$. Let $k_*^{(v)} = \min\{k \geqslant 0 : T_{k+1}^{(v)} = T_k^{(v)}\}$, $T_\infty^{(v)} = T_{k_*^{(v)}}^{(v)}$ and $\bar{T}_\infty^{(v)} = T_\infty^{(v)}/N^{(v)}$. Note that

$$\bar{T}_\infty^{(v)} = \inf\left\{t > 0 : t = \bar{T}_0^{(v)} + \frac{A_{\bullet}^{(v)}(t)}{N^{(v)}}\right\}. \tag{9.6}$$

Let $Z_{\bullet}^{(v)}(t)$ and $\tilde{Z}_E^{(v)}$ be the total number of households infected in the epidemics $\hat{\mathcal{E}}^{(v)}(t)$ and $\tilde{\mathcal{E}}^{(v)}$, respectively. Observe that $\tilde{Z}_E^{(v)} = Z_{\bullet}^{(v)}(\bar{T}_\infty^{(v)})$. Other final outcome

quantities associated with $\tilde{\mathcal{E}}^{(v)}$, for example the total number of individuals infected of a given type or the number of fully infected households, are given by the corresponding quantities of $\hat{\mathcal{E}}^{(v)}(\bar{T}_\infty^{(v)})$.

Remark 9.3 *The only difference between the epidemics $\mathcal{E}^{(v)}$ and $\tilde{\mathcal{E}}^{(v)}$ is in their initial conditions. The epidemic $\mathcal{E}^{(v)}$ is started by a set of initial infectives, while the epidemic $\tilde{\mathcal{E}}^{(v)}$ is initiated by exposing the population to $T_0^{(v)}$ units of global infection. Asymptotic properties of the epidemic $\tilde{\mathcal{E}}^{(v)}$ as $v \to \infty$ are readily available, since $\tilde{\mathcal{E}}^{(v)}$ is derived from the process $\hat{\mathcal{E}}^{(v)}$, which is the sum of independent processes. The construction of $\hat{\mathcal{E}}^{(v)}$ can be changed so that the final outcome of $\tilde{\mathcal{E}}^{(v)}$ has the same distribution as the final outcome of $\mathcal{E}^{(v)}$. To do that we simply set $T_0^{(v)}$ and the tolerances (L_{ij}s) of the initial infectives all to zero. The distribution of $\tilde{\mathcal{E}}_{\boldsymbol{n},k}$ for those households that contain initial infectives is changed but as there are only finitely many such households that change does not affect our asymptotic analysis of major outbreaks.*

9.2.2 Law of large numbers.

For $t \geqslant 0$, let

$$a_{\boldsymbol{n}}(t) = \mathbb{E}\left[A^{(\boldsymbol{n})}(t)\right] = \sum_{i=1}^{J} \beta_i \mathbb{E}\left[\tilde{A}_i^{(\boldsymbol{n})}(t)\right] = \sum_{i=1}^{J} \tilde{\mu}_{\boldsymbol{n},i}(\Lambda^L(\boldsymbol{n}), \mathrm{e}^{-t\boldsymbol{\kappa}})\mu_I^{(i)}\beta_i \quad (\boldsymbol{n} \in \mathcal{N}) \tag{9.7}$$

and $a(t) = \sum_{\boldsymbol{n}\in\mathcal{N}} \alpha_{\boldsymbol{n}} a_{\boldsymbol{n}}(t)$. Let $m_H = \lim_{v\to\infty} N^{(v)}/m^{(v)} = \sum_{\boldsymbol{n}\in\mathcal{N}} \alpha_{\boldsymbol{n}} \|\boldsymbol{n}\|$ be the asymptotic mean household size.

Theorem 9.2 *The function $a(t)$ is strictly increasing and concave on $[0,\infty)$ and satisfies*

(a) $a(0) = 0$ *and* $a(\infty) = \sum_{\boldsymbol{n}\in\mathcal{N}} \alpha_{\boldsymbol{n}} \sum_{i=1}^{J} \beta_i n_i \mu_I^{(i)}$,
(b) $a'(0) = m_H R_*$.

Proof To prove the theorem, it is fruitful to obtain an alternative expression to (9.7) for $a_{\boldsymbol{n}}(t)$, using the local susceptibility set $\mathcal{S}_{ij}^{(\boldsymbol{n})}$ of individual (i,j) in the directed random graph $\mathcal{G}_{\boldsymbol{n}}(\Lambda^L(\boldsymbol{n}))$; see Section 5.2. For ease of readability, we repeat here the definition and associated notation. Fix $\boldsymbol{n} \in \mathcal{N}$ and consider the directed graph $\mathcal{G}_{\boldsymbol{n}}(\Lambda^L(\boldsymbol{n}))$, with individuals labelled as in Section 9.2.1. Let $\mathcal{V}^{(\boldsymbol{n})} = \{(i,j) : i = 1,2,\dots,J, j = 1,2,\dots,n_i\}$ be the vertex set of $\mathcal{G}_{\boldsymbol{n}}(\Lambda^L(\boldsymbol{n}))$ and, for $i \in \mathcal{J}$, let $\mathcal{V}_i^{(\boldsymbol{n})} = \{(i,j) : j = 1,2,\dots,n_i\}$ be the set of type-i vertices. For $((i,j),(i',j')) \in (\mathcal{V}^{(\boldsymbol{n})})^2$, write $(i,j) \rightsquigarrow (i',j')$ if there is a chain of directed edges from (i,j) to (i',j') in $\mathcal{G}_{\boldsymbol{n}}(\Lambda^L(\boldsymbol{n}))$, with the convention that $(i,j) \rightsquigarrow (i,j)$. For $(i,j) \in \mathcal{V}^{(\boldsymbol{n})}$, let $\mathcal{S}_{ij}^{(\boldsymbol{n})} = \{(i',j') \in \mathcal{V}^{(\boldsymbol{n})} : (i',j') \rightsquigarrow (i,j)\}$ be the local susceptibility set of individual (i,j) and, for $k \in \mathcal{J}$, let $\mathcal{S}_{ij}^{(\boldsymbol{n})}(k) = \mathcal{S}_{ij}^{(\boldsymbol{n})} \cap \mathcal{V}_k^{(\boldsymbol{n})}$ be the set of type-k individuals in (i,j)'s susceptibility set and $S_{ij}^{(\boldsymbol{n})}(k) = |\mathcal{S}_{ij}^{(\boldsymbol{n})}(k)|$. (Note that $\mathcal{S}_{ij}^{(\boldsymbol{n})}$ is defined only if $\boldsymbol{n} \in \mathcal{N}_i$ and $\mathcal{S}_{ij}^{(\boldsymbol{n})}(k)$ is the empty set (and $S_{ij}^{(\boldsymbol{n})}(k) = 0$) if $\boldsymbol{n} \notin \mathcal{N}_k$). In the following we use the convention that

$S_{ij}^{(\boldsymbol{n})}(k) = 0$ and $\mu_{\boldsymbol{n},i,k}(\Lambda^L(\boldsymbol{n})) = 0$ if $\boldsymbol{n} \notin \mathcal{N}_i \cap \mathcal{N}_k$, where $\mu_{\boldsymbol{n},i,k}(\Lambda^L(\boldsymbol{n}))$ is defined in Section 3.1.) Also let

$$\chi_{ij}^{(\boldsymbol{n})}(t) = \begin{cases} 1 & \text{if } (i,j) \text{ is infected in } \tilde{\mathcal{E}}_{\boldsymbol{n}}(t), \\ 0 & \text{otherwise,} \end{cases}$$

so

$$\tilde{A}_i^{(\boldsymbol{n})}(t) = \sum_{j=1}^{n_i} I_{ij}\chi_{ij}^{(\boldsymbol{n})}(t) \qquad (i \in \mathcal{J}, t \geqslant 0).$$

Note that I_{ij} is independent of $\chi_{ij}^{(\boldsymbol{n})}(t)$ and that $\chi_{ij}^{(\boldsymbol{n})}(t) = 0$ if and only if all members of $\mathcal{S}_{ij}^{(\boldsymbol{n})}$ avoid global infection, which, conditional upon $\mathcal{S}_{ij}^{(\boldsymbol{n})}$, occurs with probability $\exp\left(-t\sum_{k=1}^{J}\kappa_k S_{ij}^{(\boldsymbol{n})}(k)\right)$. Thus, by symmetry,

$$\mathbb{E}\left[\tilde{A}_i^{(\boldsymbol{n})}(t)\right] = n_i\mu_I^{(i)}\mathbb{E}\left[\chi_{i1}^{(\boldsymbol{n})}(t)\right] = n_i\mu_I^{(i)}\left(1 - \mathbb{E}\left[\exp\left(-t\sum_{k=1}^{J}\kappa_k S_{i1}^{(\boldsymbol{n})}(k)\right)\right]\right),$$

whence

$$\begin{aligned} a(t) &= \sum_{\boldsymbol{n}\in\mathcal{N}} \alpha_{\boldsymbol{n}} \sum_{i=1}^{J} \beta_i \mathbb{E}\left[\tilde{A}_i^{(\boldsymbol{n})}(t)\right] \\ &= \sum_{\boldsymbol{n}\in\mathcal{N}} \alpha_{\boldsymbol{n}} \sum_{i=1}^{J} n_i\mu_I^{(i)}\beta_i\left(1 - \mathbb{E}\left[\exp\left(-t\sum_{j=1}^{J}\kappa_j S_{i1}^{(\boldsymbol{n})}(j)\right)\right]\right). \end{aligned} \tag{9.8}$$

Differentiating (9.8) yields $a'(t) > 0$ and $a''(t) < 0$, so $a(t)$ is strictly increasing and concave on $[0,\infty)$. Setting $t = 0$ and $t = \infty$ in (9.8) yields part (a). Finally,

$$a'(0) = \sum_{\boldsymbol{n}\in\mathcal{N}} \alpha_{\boldsymbol{n}} \sum_{i=1}^{J} n_i\mu_I^{(i)}\beta_i \sum_{j=1}^{J} \kappa_j \mathbb{E}\left[S_{i1}^{(\boldsymbol{n})}(j)\right]. \tag{9.9}$$

Returning to the random directed graph $\mathcal{G}_{\boldsymbol{n}}(\Lambda^L(\boldsymbol{n}))$, note that

$$\begin{aligned} n_i\mathbb{E}\left[S_{i1}^{(\boldsymbol{n})}(j)\right] &= \sum_{k=1}^{n_i}\mathbb{E}\left[S_{ik}^{(\boldsymbol{n})}(j)\right] = \sum_{k=1}^{n_i}\sum_{\ell=1}^{n_j}\mathbb{E}\left[\mathbb{1}_{\{(j,\ell)\rightsquigarrow(i,k)\}}\right] \\ &= n_j\sum_{k=1}^{n_i}\mathbb{E}\left[\mathbb{1}_{\{(j,1)\rightsquigarrow(i,k)\}}\right] = n_j\mu_{\boldsymbol{n},j,i}(\Lambda^L(\boldsymbol{n})). \end{aligned} \tag{9.10}$$

Substituting (9.10) into (9.9) yields, after switching the indices i and j,

$$a'(0) = \sum_{\boldsymbol{n}\in\mathcal{N}} \alpha_{\boldsymbol{n}} \sum_{i=1}^{J}\sum_{j=1}^{J} n_i\kappa_i\mu_{\boldsymbol{n},i,j}(\Lambda^L(\boldsymbol{n}))\mu_I^{(j)}\beta_j. \tag{9.11}$$

Comparing (9.11) with (4.1) shows that $a'(0) = m_H R_*$, since

$$\gamma_i\alpha_i(\boldsymbol{n}) = \lim_{v\to\infty}\gamma_i^{(v)}\alpha_i^{(v)}(\boldsymbol{n}) = \lim_{v\to\infty}\frac{N_i^{(v)}}{N^{(v)}}\frac{n_i m_{\boldsymbol{n}}^{(v)}}{N_i^{(v)}} = \lim_{v\to\infty} n_i\alpha_{\boldsymbol{n}}^{(v)}\frac{m^{(v)}}{N^{(v)}} = m_H^{-1}n_i\alpha_{\boldsymbol{n}}.$$

□

Recall that $Z_\bullet^{(v)}(t)$ is the total number of households infected in the epidemic $\hat{\mathcal{E}}^{(v)}(t)$. Hence, for $t \geqslant 0$,

$$Z_\bullet^{(v)}(t) = \sum_{\boldsymbol{n}\in\mathcal{N}} \sum_{k=1}^{m_{\boldsymbol{n}}^{(v)}} \mathbb{1}_{\{\text{household } (\boldsymbol{n},k) \text{ is infected in } \tilde{\mathcal{E}}_{\boldsymbol{n},k}(t)\}},$$

so

$$\mathbb{E}\left[Z_\bullet^{(v)}(t)\right] = \sum_{\boldsymbol{n}\in\mathcal{N}} m_{\boldsymbol{n}}^{(v)} \left(1 - \mathrm{e}^{-t\boldsymbol{n}\boldsymbol{\kappa}^\top}\right).$$

Let

$$z_E(t) = \lim_{v\to\infty} \frac{1}{m^{(v)}} \mathbb{E}\left[Z_\bullet^{(v)}(t)\right] = \sum_{\boldsymbol{n}\in\mathcal{N}} \alpha_{\boldsymbol{n}} (1 - \mathrm{e}^{-t\boldsymbol{n}\boldsymbol{\kappa}^\top}). \tag{9.12}$$

Theorem 9.3 *As $v \to \infty$,*

$$\sup_{t\geqslant 0} \left| \frac{1}{m^{(v)}} A_\bullet^{(v)}(t) - a(t) \right| \xrightarrow{\text{a.s.}} 0 \qquad \textit{and} \qquad \sup_{t\geqslant 0} \left| \frac{1}{m^{(v)}} Z_\bullet^{(v)}(t) - z_E(t) \right| \xrightarrow{\text{a.s.}} 0. \tag{9.13}$$

Proof We prove the first result in (9.13). The second result is proved in the same fashion. The strong law of large numbers can be applied to each household category separately to give that, for each $t \in [0, \infty)$ and each $\boldsymbol{n} \in \mathcal{N}$,

$$\frac{1}{m^{(v)}} \sum_{k=1}^{m_{\boldsymbol{n}}^{(v)}} A^{(\boldsymbol{n},k)}(t) = \alpha_{\boldsymbol{n}}^{(v)} \frac{1}{m_{\boldsymbol{n}}^{(v)}} \sum_{k=1}^{m_{\boldsymbol{n}}^{(v)}} A^{(\boldsymbol{n},k)}(t) \xrightarrow{\text{a.s.}} \alpha_{\boldsymbol{n}} a_{\boldsymbol{n}}(t) \quad \text{as } v \to \infty.$$

Since there are a finite number of household categories, we sum these to obtain

$$\frac{1}{m^{(v)}} A_\bullet^{(v)}(t) \xrightarrow{\text{a.s.}} a(t) \text{ as } v \to \infty. \tag{9.14}$$

Note that $A_\bullet^{(v)}(\infty)$ is a weighted sum of the infectious periods of all individuals in the population, and hence almost surely finite, and $a(\infty) < \infty$, so (9.14) holds also when $t = \infty$. Further, both $A_\bullet^{(v)}(t)$ and $a(t)$ are nondecreasing in t. A similar argument to the proof of the Glivenko-Cantelli theorem shows that the convergence in (9.14) holds uniformly in t; see, for example, the proof of Ball and Britton [2], Lemma 1. □

Suppose that $\bar{T}_0^{(v)} \xrightarrow{\text{a.s.}} 0$ as $v \to \infty$. Recall that $N^{(v)}/m^{(v)} \to m_H$ as $v \to \infty$. Letting $v \to \infty$ in the equation satisfied by $\bar{T}_\infty^{(v)}$ in (9.6) and using the first result in Theorem 9.3 leads to the following equation

$$t = m_H^{-1} a(t). \tag{9.15}$$

Lemma 9.1 *If $R_* \leqslant 1$, then $t = 0$ is the only solution in $[0, \infty)$ of (9.15). If $R_* > 1$, then there is a unique solution, τ say, in $(0, \infty)$ and $a'(\tau) < m_H$.*

Proof Recall from Theorem 9.2 that $a(0) = 0$, $a'(t)$ is strictly decreasing on $[0, \infty)$ and $a'(0) = m_H R_*$. If $R_* \leqslant 1$ then, for $t > 0$,

$$a(t) = \int_0^t a'(u)\,\mathrm{d}u < \int_0^t m_H R_*\,\mathrm{d}u \leqslant m_H t,$$

so $t = 0$ is the only solution in $[0, \infty)$ of (9.15).

Suppose $R_* > 1$. Then, there is a unique solution (τ) of (9.15) in $(0, \infty)$, since, by Theorem 9.2, $a(t)$ is concave with $a(0) = 0$, $a(\infty) < \infty$ and $a'(0) = m_H R_* > m_H$. Suppose, for contradiction, that $a'(\tau) \geqslant m_H$. Then, since $a'(t)$ is strictly decreasing on $[0, \infty)$,

$$a(\tau) = \int_0^\tau a'(u)\,\mathrm{d}u > \int_0^\tau m_H\,\mathrm{d}u = m_H \tau,$$

which is a contradiction as $a(\tau) = m_H \tau$. □

Remark 9.4 *Note that (9.15) is the same equation as (5.1), since* $\frac{\tilde{\alpha}_{\boldsymbol{n}}}{\|\boldsymbol{n}\|} = \frac{m_{\boldsymbol{n}}}{N} = m_H^{-1}\frac{m_{\boldsymbol{n}}}{m} = m_H^{-1}\alpha_{\boldsymbol{n}}$. *Thus, properties of the solutions of (5.1) claimed in Section 5.1 follow immediately from Lemma 9.1.*

We now assume that $R_* > 1$ and $\bar{T}_0^{(v)} \xrightarrow{\text{a.s.}} 0$ as $v \to \infty$. The first result in (9.13) implies that

$$\sup_{t \geqslant 0} \left| \bar{T}_0^{(v)} + A_\bullet^{(v)}(t) N^{(v)} - m_H^{-1} a(t) \right| \xrightarrow{\text{a.s.}} 0 \qquad \text{as } v \to \infty. \tag{9.16}$$

Let $\epsilon \in (0, \tau/2)$. It follows from Theorem 9.2 and $a'(\tau) < m_H$ that there exists $\delta > 0$ such that $m_H^{-1} a(t) > t + \delta$ for $t \in [\epsilon, \tau - \epsilon]$ and $m_H^{-1} a(t) < t - \delta$ for $t \in [\tau + \epsilon, \infty)$. Using (9.16) and the definition of $\bar{T}_\infty^{(v)}$ at (9.6), it follows that, almost surely, for all $\epsilon \in (0, \tau/2)$, $\bar{T}_\infty^{(v)} \notin [\epsilon, \tau - \epsilon] \cup [\tau + \epsilon, \infty)$ for all sufficiently large v. Thus,

$$\min\{\bar{T}_\infty^{(v)}, |\bar{T}_\infty^{(v)} - \tau|\} \xrightarrow{\text{a.s.}} 0 \qquad \text{as } v \to \infty. \tag{9.17}$$

Further, recalling that $\tilde{Z}_E^{(v)} = Z_\bullet^{(v)}(\bar{T}_\infty^{(v)})$ and using the second result in Theorem 9.3,

$$\min\{(m^{(v)})^{-1} \tilde{Z}_E^{(v)}, |(m^{(v)})^{-1} \tilde{Z}_E^{(v)} - z_E|\} \xrightarrow{\text{a.s.}} 0 \qquad \text{as } v \to \infty, \tag{9.18}$$

where $z_E = z_E(\tau)$, with $z_E(t)$ being given by (9.12).

Recall that $Z_E^{(v)}$ is the total number of households infected in $\mathcal{E}^{(v)}$ and $G^{(v)} = \{Z_E^{(v)} \geqslant \log m^{(v)}\}$. Let $\bar{Z}_E^{(v)} = Z_E^{(v)}/m^{(v)}$.

Theorem 9.4 *Suppose $R_* > 1$. Then,*

$$\mathcal{L}\left(\bar{Z}_E^{(v)} \mid G^{(v)}\right) \xrightarrow{\text{D}} \delta_{z_E} \qquad \text{as } v \to \infty,$$

where δ_{z_E} is the point mass at z_E.

Proof Following Remark 9.3, we construct a realisation of the final outcome of $\mathcal{E}^{(v)}$ from the process $\hat{\mathcal{E}}^{(v)}$ by setting $T_0^{(v)}$ and the tolerances of the initial infectives to zero. Recall that the configuration of initial infectives is assumed to be the same for all v. For $\boldsymbol{n} \in \mathcal{N}$, let $h_{\boldsymbol{n}}$ be the number of households of category $\boldsymbol{n}$ that contain initial infectives. We construct a process $\hat{\mathcal{E}}^{(v)'} = \{\hat{\mathcal{E}}^{(v)'}(t) : t \geqslant 0\}$, in the same way as $\hat{\mathcal{E}}^{(v)}$, except for $\boldsymbol{n} \in \mathcal{N}$ and $k = 1, 2, \ldots, h_{\boldsymbol{n}}$, the process $\tilde{\mathcal{E}}_{\boldsymbol{n},k}$ is replaced by the process $\tilde{\mathcal{E}}'_{\boldsymbol{n},k}$, which is defined similarly to $\tilde{\mathcal{E}}_{\boldsymbol{n},k}$ except the tolerances corresponding to initial infectives are set to zero. Note that $\tilde{\mathcal{E}}'_{\boldsymbol{n},k}$ ($\boldsymbol{n} \in \mathcal{N}, k = 1, 2, \ldots, h_{\boldsymbol{n}}$) are independent but $\tilde{\mathcal{E}}'_{\boldsymbol{n},k}$ ($k = 1, 2, \ldots, h_{\boldsymbol{n}}$) may not be identically distributed, since the configuration of initial infectives in these households may not all be the same.

In an obvious notation, let

$$A_{\bullet}^{(v)'}(t) = \sum_{\boldsymbol{n}\in\mathcal{N}} \sum_{k=1}^{h_{\boldsymbol{n}}} A^{(\boldsymbol{n},k)'}(t) + \sum_{\boldsymbol{n}\in\mathcal{N}} \sum_{k=h_{\boldsymbol{n}}+1}^{m_{\boldsymbol{n}}^{(v)}} A^{(\boldsymbol{n},k)}(t) \qquad (t \geqslant 0) \tag{9.19}$$

(cf. (9.5)) and define $Z_{\bullet}^{(v)'}(t)$ $(t \geqslant 0)$ similarly. The first sum in (9.19) is over finitely many households and is independent of v, as is the corresponding sum in $Z_{\bullet}^{(v)'}(t)$. It follows that Theorem 9.3 holds with $A_{\bullet}^{(v)}(t)$ replaced by $A_{\bullet}^{(v)'}(t)$ and $Z_{\bullet}^{(v)}(t)$ replaced by $Z_{\bullet}^{(v)'}(t)$. The argument leading to (9.18), applied to the process $\hat{\mathcal{E}}^{(v)'}$, then yields

$$\min\{\bar{Z}_E^{(v)}, |\bar{Z}_E^{(v)} - z_E|\} \xrightarrow{\text{a.s.}} 0 \qquad \text{as } v \to \infty. \tag{9.20}$$

Now

$$\limsup_{v\to\infty} \mathbb{P}\left(\bar{Z}_E^{(v)} \geqslant \frac{z_E}{2}\right) \leqslant \limsup_{v\to\infty} \mathbb{P}\left(Z_E^{(v)} \geqslant \log m^{(v)}\right) = 1 - p_{\text{ext}}, \tag{9.21}$$

by Theorem 9.1. Turning to $\liminf_{v\to\infty} \mathbb{P}\left(\bar{Z}_E^{(v)} \geqslant \frac{z_E}{2}\right)$, first note that (9.20) implies that, for any $\delta \in (0, z_E/2)$,

$$\liminf_{v\to\infty} \mathbb{P}\left(\bar{Z}_E^{(v)} \geqslant \frac{z_E}{2}\right) = \liminf_{v\to\infty} \mathbb{P}\left(\bar{Z}_E^{(v)} \geqslant \delta\right). \tag{9.22}$$

To obtain a lower bound for $\mathbb{P}(\bar{Z}_E^{(v)} \geqslant \delta)$ we use a device first introduced by Whittle [12]. Using the bound at (9.1), note that, while the total number of infected households in $\mathcal{E}^{(v)}$ is not more than $\delta m^{(v)}$, the probability that a global contact is with a previously uninfected household is at least $1 - \delta_*^{(v)}$, where

$$\delta_*^{(v)} = \min\left\{1, \delta n_{\max}/\gamma_{\min}^{(v)}\right\},$$

with $\gamma_{\min}^{(v)} = \min_{i\in\mathcal{J}} \gamma_i^{(v)}$. Thus, while the total number of infected households in $\mathcal{E}^{(v)}$ is not more than $\delta m^{(v)}$, the process of infected households in $\mathcal{E}^{(v)}$ can be bounded below by a branching process, $\mathcal{B}^{(v)}(\delta)$, which is defined analogously to $\mathcal{B}^{(v)}$ in Section 9.1 except, apart from the ancestors, individuals are deleted at birth independently with probability $\delta_*^{(v)}$. (Individuals deleted at birth have no offspring and do not contribute to the total progeny.) Let $Z^{(v)}(\delta)$ and $p_{\text{ext}}^{(v)}(\delta)$ be respectively the total progeny and extinction probability of $\mathcal{B}^{(v)}(\delta)$. Then,

$$\liminf_{v\to\infty} \mathbb{P}\left(\bar{Z}_E^{(v)} \geqslant \delta\right) \geqslant \liminf_{v\to\infty} \mathbb{P}\left(Z^{(v)}(\delta) \geqslant \delta m^{(v)}\right) \geqslant \liminf_{v\to\infty}(1 - p_{\text{ext}}^{(v)}(\delta)) = 1 - p_{\text{ext}}(\delta), \tag{9.23}$$

where $p_{\text{ext}}(\delta)$ is the extinction probability of the limiting branching process, $\mathcal{B}(\delta)$, derived from $\mathcal{B}$ by independently deleting individuals at birth with probability $\delta_* =$

$\min\{1, \delta n_{\max}/\gamma_{\min}\}$. Now $p_{\text{ext}}(\delta) \downarrow p_{\text{ext}}$ as $\delta \downarrow 0$. Thus, combining (9.22) and (9.23) and letting $\delta \downarrow 0$ yields

$$\liminf_{v\to\infty} \mathbb{P}\left(\bar{Z}_E^{(v)} \geqslant \frac{z_E}{2}\right) \geqslant 1 - p_{\text{ext}},$$

which together with (9.21) gives

$$\lim_{v\to\infty} \mathbb{P}\left(\bar{Z}_E^{(v)} \geqslant \frac{z_E}{2}\right) = 1 - p_{\text{ext}}. \tag{9.24}$$

It follows from (9.20) and (9.24) that for any $\epsilon \in (0, z_E/2)$,

$$\lim_{v\to\infty} \mathbb{P}\left(z_E - \epsilon < \bar{Z}_E^{(v)} < z_E + \epsilon\right) = 1 - p_{\text{ext}}.$$

Now $m^{(v)}(z_E - \epsilon) \geqslant \log m^{(v)}$ for all sufficiently large v, so

$$\lim_{v\to\infty} \mathbb{P}\left(z_E - \epsilon < \bar{Z}_E^{(v)} < z_E + \epsilon, G^{(v)}\right) = \lim_{v\to\infty} \mathbb{P}\left(z_E - \epsilon < \bar{Z}_E^{(v)} < z_E + \epsilon\right).$$

Hence, since $\mathbb{P}\left(G^{(v)}\right) \to 1 - p_{\text{ext}}$ as $v \to \infty$, by Theorem 9.1, and $p_{\text{ext}} < 1$ as $R_* > 1$, we have that for any $\epsilon \in (0, z_E/2)$,

$$\begin{aligned}
\lim_{v\to\infty} \mathbb{P}\left(z_E - \epsilon < \bar{Z}_E^{(v)} < z_E + \epsilon \mid G^{(v)}\right) &= \lim_{v\to\infty} \frac{\mathbb{P}\left(z_E - \epsilon < \bar{Z}_E^{(v)} < z_E + \epsilon, G^{(v)}\right)}{\mathbb{P}\left(G^{(v)}\right)} \\
&= \frac{\lim_{v\to\infty} \mathbb{P}\left(z_E - \epsilon < \bar{Z}_E^{(v)} < z_E + \epsilon, G^{(v)}\right)}{\lim_{v\to\infty} \mathbb{P}(G^{(v)})} \\
&= \frac{1 - p_{\text{ext}}}{1 - p_{\text{ext}}} = 1,
\end{aligned}$$

proving the theorem. □

Remark 9.5 *Note that Theorem 9.4 holds if the definition of a major outbreak is replaced by the one given in Remark 9.1. Further, it is clear from Theorem 9.4 that $G^{(v)}$ can be replaced by $G^{(v)} = \{Z_E^{(v)} \geqslant f(m^{(v)})\}$, where $f : \mathbb{N} \to \mathbb{R}_+$ is any nondecreasing function satisfying $f(m) \to \infty$ and $m^{-1}f(m) \to 0$ as $m \to \infty$. Indeed, a major outbreak infects at least a fraction $z_E > 0$ of households. However, z_E depends on the parameters of the model and can be arbitrarily close to zero.*

Remark 9.6 *Analogous results to Theorem 9.4 for other properties of a major outbreak, such as those mentioned in Remark 9.2, can be proved in a similar fashion.*

9.2.3 Central limit theorem.

We now prove a multivariate central limit theorem for quantities associated with the final outcome of a major outbreak. For $\boldsymbol{n} \in \mathcal{N}$, consider the process $\tilde{\mathcal{E}}_{\boldsymbol{n}} = \{\tilde{\mathcal{E}}_{\boldsymbol{n}}(t) : t \geqslant 0\}$ defined in Section 9.2.1. For $t \geqslant 0$, let $R_1^{(\boldsymbol{n})}(t), R_2^{(\boldsymbol{n})}(t), \ldots, R_p^{(\boldsymbol{n})}(t)$ be a collection of p final outcome quantities defined on the epidemic $\tilde{\mathcal{E}}_{\boldsymbol{n}}(t)$. Some examples are given in Section 5.3. Let $\boldsymbol{R}^{(\boldsymbol{n})}(t) = (R_1^{(\boldsymbol{n})}(t), R_2^{(\boldsymbol{n})}(t), \ldots, R_p^{(\boldsymbol{n})}(t), A^{(\boldsymbol{n})}(t))^\top$. (Note that this notation is slightly different from that in Section 5.3, where $\boldsymbol{R}^{(\boldsymbol{n})}(t) = (R_1^{(\boldsymbol{n})}(t), R_2^{(\boldsymbol{n})}(t), \ldots, R_p^{(\boldsymbol{n})}(t))^\top$. For the proof, it is convenient to absorb the weighted

severity, which is another final outcome quantity, into the vector of final outcome quantities.) As in Section 9.2.1, a single realisation of $\mathcal{G}_{\boldsymbol{n}}(\Lambda^L(\boldsymbol{n}))$ and (L_{ij}, I_{ij}) $(i \in \mathcal{J}, j = 1, 2, \ldots, n_i)$ is used to define $\boldsymbol{R}^{(\boldsymbol{n})}(t)$ for all $t \geqslant 0$, and hence the process $\{\boldsymbol{R}^{(\boldsymbol{n})}(t)\} = \{\boldsymbol{R}^{(\boldsymbol{n})}(t) : t \geqslant 0\}$. Consequently, the jumps of the process $\{R_i^{(\boldsymbol{n})}(t)\}$ can occur only when $t = L_{ij}$ for some (i, j), i.e. only when a new individual is infected externally in the process of epidemics $\tilde{\mathcal{E}}_{\boldsymbol{n}}$.

Let $\{\boldsymbol{R}^{(\boldsymbol{n},k)}(t)\}$ $(\boldsymbol{n} \in \mathcal{N}, k = 1, 2, \ldots)$ be independent with $\{\boldsymbol{R}^{(\boldsymbol{n},k)}(t)\}$ distributed as $\{\boldsymbol{R}^{(\boldsymbol{n})}(t)\}$. For $v = 1, 2, \ldots$, let $\{\boldsymbol{R}_\bullet^{(v)}(t)\}$ be the process defined by

$$\boldsymbol{R}_\bullet^{(v)}(t) = \sum_{\boldsymbol{n} \in \mathcal{N}} \sum_{k=1}^{m_{\boldsymbol{n}}^{(v)}} \boldsymbol{R}^{(\boldsymbol{n},k)}(t) \qquad (t \geqslant 0). \tag{9.25}$$

For each $t \geqslant 0$, the elements of $\boldsymbol{R}_\bullet^{(v)}(t)$ contain final outcome quantities for the epidemic $\hat{\mathcal{E}}^{(v)}(t)$. The corresponding final outcome quantities for the epidemics $\tilde{\mathcal{E}}^{(v)}$ and $\mathcal{E}^{(v)}$ can be obtained via the embedding construction described in Section 9.2.1.

We assume that there exists $\zeta > 0$ such that

$$\mathbb{E}[I_{i1}^{2+\zeta}] < \infty \qquad (i \in \mathcal{J}) \tag{9.26}$$

and

$$\mathbb{E}\left[\left(\sup_{t \geqslant 0} \left|R_j^{(\boldsymbol{n})}(t)\right|\right)^{2+\zeta}\right] < \infty \qquad (\boldsymbol{n} \in \mathcal{N}, j = 1, 2, \ldots, p). \tag{9.27}$$

We now introduce some more notation. For conciseness, we define $R_{p+1}^{(\boldsymbol{n})}(t) = A^{(\boldsymbol{n})}(t)$. For $j = 1, 2, \ldots, p+1$, $\boldsymbol{n} \in \mathcal{N}$ and $t \geqslant 0$, let $r_j^{(\boldsymbol{n})}(t) = \mathbb{E}[R_j^{(\boldsymbol{n})}(t)]$, $r_j^{(v)}(t) = \sum_{\boldsymbol{n} \in \mathcal{N}} \alpha_{\boldsymbol{n}}^{(v)} r_j^{(\boldsymbol{n})}(t)$ and $r_j(t) = \sum_{\boldsymbol{n} \in \mathcal{N}} \alpha_{\boldsymbol{n}} r_j^{(\boldsymbol{n})}(t)$. Let $\boldsymbol{r}(t) = (r_1(t), r_2(t), \ldots, r_{p+1}(t))^\top$. Let $\boldsymbol{C}^{(\boldsymbol{n})}(t, s) = [c_{ij}^{(\boldsymbol{n})}(t, s)]$ be the covariance function with elements

$$c_{ij}^{(\boldsymbol{n})}(t, s) = \operatorname{cov}\left(R_i^{(\boldsymbol{n})}(t), R_j^{(\boldsymbol{n})}(s)\right), \qquad 0 \leqslant t, s < \infty, \ i, j = 1, 2, \ldots, p+1.$$

Conditions (9.26) and (9.27) ensure that these covariances are all finite. Note that $\boldsymbol{C}^{(\boldsymbol{n})}(t, t) = \boldsymbol{C}^{(\boldsymbol{n})}(t)$, where $\boldsymbol{C}^{(\boldsymbol{n})}(t)$ is defined at (5.8).

Note that the final outcome of the epidemic $\tilde{\mathcal{E}}^{(v)}$ is given by $\boldsymbol{R}_\bullet^{(v)}(\bar{T}_\infty^{(v)})$. For $T > 0$, let $\boldsymbol{R}_{\bullet,T}^{(v)} = \{\boldsymbol{R}_\bullet^{(v)}(t) : 0 \leqslant t \leqslant T\}$.

Theorem 9.5 *For any $T > 0$,*

$$\frac{1}{\sqrt{m^{(v)}}}\left(\boldsymbol{R}_{\bullet,T}^{(v)} - \mathbb{E}[\boldsymbol{R}_{\bullet,T}^{(v)}]\right) \xrightarrow{\text{w}} \boldsymbol{X}_T \ \text{as } v \to \infty,$$

where $\boldsymbol{X}_T = \{(X_1(t), X_2(t), \ldots, X_p(t), X_A(t))^\top : 0 \leqslant t \leqslant T\}$ is a zero-mean Gaussian process with covariance function $\boldsymbol{C}(t, s) = \sum_{\boldsymbol{n} \in \mathcal{N}} \alpha_{\boldsymbol{n}} \boldsymbol{C}^{(\boldsymbol{n})}(t, s)$ $(t, s \in [0, T])$ and $\xrightarrow{\text{w}}$ denotes weak convergence in the space of bounded functions from $[0, T]$ to $\mathbb{R}^{p+1}$ endowed with the supremum metric.

Proof The proof uses the usual method of showing convergence of finite-dimensional distributions and asymptotic tightness (see, for example, van der Vaart and Wellner [11], Theorem 1.5.4). It is quite long, so details are given in Section 9.2.4. □

Suppose $R_* > 1$ and $\bar{T}_0^{(v)} \xrightarrow{\text{a.s.}} 0$ as $v \to \infty$. For $\epsilon \in (0, \tau)$, define $\bar{T}_{\infty,\epsilon}^{(v)}$ to be the smallest solution in $[\epsilon, \infty)$ of $t = \bar{T}_0^{(v)} + \frac{1}{N^{(v)}} A_\bullet^{(v)}(t)$, provided one exists, with $\bar{T}_{\infty,\epsilon}^{(v)} = \bar{T}_\infty^{(v)}$ otherwise. We use Theorem 9.5 and the crossing problem satisfied by $\bar{T}_{\infty,\epsilon}^{(v)}$ to obtain a central limit theorem for $\boldsymbol{R}_\bullet^{(v)}(\bar{T}_{\infty,\epsilon}^{(v)})$. Let

$$\boldsymbol{H} = \begin{pmatrix} \boldsymbol{I}_p & \frac{1}{m_H - a'(\tau)} \tilde{\boldsymbol{r}}'(\tau) \\ \boldsymbol{0}_p^T & \frac{m_H}{m_H - a'(\tau)} \end{pmatrix}, \tag{9.28}$$

where $\tilde{\boldsymbol{r}}'(\tau) = (r_1'(\tau), r_2'(\tau), \dots, r_p'(\tau))^\top$. Note that $\boldsymbol{H}$ is well-defined since Lemma 9.1 implies $m_H > a'(\tau)$.

Theorem 9.6 *Suppose that $R_* > 1$ and, as $v \to \infty$,*

(i) $\sqrt{m^{(v)}} \left(\alpha_{\boldsymbol{n}}^{(v)} - \alpha_{\boldsymbol{n}} \right) \to 0 \quad (\boldsymbol{n} \in \mathcal{N})$,

(ii) $\sqrt{m^{(v)}} \bar{T}_0^{(v)} \xrightarrow{\text{p}} 0$.

Then, for any $\epsilon \in (0, \tau)$,

$$m^{(v)^{-\frac{1}{2}}} \left(\boldsymbol{R}_\bullet^{(v)}(\bar{T}_{\infty,\epsilon}^{(v)}) - m^{(v)} \boldsymbol{r}(\tau) \right) \xrightarrow{\text{D}} \mathrm{N}(\boldsymbol{0}_{p+1}, \boldsymbol{H}\boldsymbol{C}(\tau,\tau)\boldsymbol{H}^\top) \qquad \textit{as } v \to \infty. \tag{9.29}$$

Proof For $j = 1, 2, \dots, p+1$ and $t \geqslant 0$,

$$\mathbb{E}[R_{\bullet,j}^{(v)}(t)] = m^{(v)} r_j^{(v)}(t) = m^{(v)} \sum_{\boldsymbol{n} \in \mathcal{N}} \alpha_{\boldsymbol{n}}^{(v)} r_j^{(\boldsymbol{n})}(t).$$

For $\boldsymbol{n} \in \mathcal{N}$ and any $T > 0$, the function $r_j^{(\boldsymbol{n})}(t)$ is continuous, and hence bounded, on $[0, T]$, so $\sqrt{m^{(v)}} \max_{0 \leqslant t \leqslant T} |r_j^{(v)}(t) - r_j(t)| \to 0$ as $v \to \infty$ by condition (i). Thus, using Theorem 9.5 and Slutsky's theorem (e.g. van der Vaart and Wellner [11], Example 1.4.7), we have that, for any $T > 0$,

$$\frac{1}{\sqrt{m^{(v)}}} \left(\boldsymbol{R}_{\bullet,T}^{(v)} - m^{(v)} \boldsymbol{r}_T \right) \xrightarrow{\text{w}} \boldsymbol{X}_T \qquad \text{as } v \to \infty, \tag{9.30}$$

where $\boldsymbol{r}_T = \{\boldsymbol{r}(t) : 0 \leqslant t \leqslant T\}$.

For $j = 1, 2, \dots, p+1$,

$$\begin{aligned} (m^{(v)})^{-\frac{1}{2}} \left[R_{\bullet,j}^{(v)}(\bar{T}_{\infty,\epsilon}^{(v)}) - m^{(v)} r_j(\tau) \right] =& (m^{(v)})^{-\frac{1}{2}} \left[R_{\bullet,j}^{(v)}(\bar{T}_{\infty,\epsilon}^{(v)}) - m^{(v)} r_j(\bar{T}_{\infty,\epsilon}^{(v)}) \right] \\ &+ (m^{(v)})^{\frac{1}{2}} \left[r_j(\bar{T}_{\infty,\epsilon}^{(v)}) - r_j(\tau) \right] \\ =& A_j^{(v)} + B_j^{(v)}, \text{ say.} \end{aligned} \tag{9.31}$$

Since condition (ii) implies that $\bar{T}_0^{(v)} \xrightarrow{\text{p}} 0$ as $v \to \infty$, it follows from Theorem 9.3 and the definition of $\bar{T}_{\infty,\epsilon}^{(v)}$ that $\bar{T}_{\infty,\epsilon}^{(v)} \xrightarrow{\text{p}} \tau$ as $v \to \infty$. Hence, using (9.30), with $T > \tau$, and the

continuous mapping theorem (e.g. van der Vaart and Wellner [11], Theorem 1.3.6), we have that

$$A_j^{(v)} \xrightarrow{\text{D}} X_j(\tau) \qquad \text{as } v \to \infty, \tag{9.32}$$

where $X_{p+1}(\tau) = X_A(\tau)$.

Turning to $B_j^{(v)}$, using the mean value theorem we have that

$$B_j^{(v)} = (m^{(v)})^{\frac{1}{2}} r_j'(\xi_j^{(v)}) \left(\bar{T}_{\infty,\epsilon}^{(v)} - \tau\right), \tag{9.33}$$

for some $\xi_j^{(v)}$ lying between $\bar{T}_{\infty,\epsilon}^{(v)}$ and τ. Now $\bar{T}_{\infty,\epsilon}^{(v)} \xrightarrow{\text{p}} \tau$ as $v \to \infty$, so by the sandwich theorem, $\xi_j^{(v)} \xrightarrow{\text{p}} \tau$ as $v \to \infty$. Also, using $\tau = m_H^{-1} a(\tau)$ and the definition of $\bar{T}_{\infty,\epsilon}^{(v)}$, and letting $m_H^{(v)} = N^{(v)}/m^{(v)}$, we have that

$$\begin{aligned}
(m^{(v)})^{\frac{1}{2}} \left(\bar{T}_{\infty,\epsilon}^{(v)} - \tau\right) &= (m^{(v)})^{\frac{1}{2}} \bar{T}_0^{(v)} + (m^{(v)})^{\frac{1}{2}} \left(\frac{1}{N^{(v)}} A_\bullet^{(v)}(\bar{T}_{\infty,\epsilon}^{(v)}) - m_H^{-1} a(\tau)\right) \\
&= (m^{(v)})^{\frac{1}{2}} \bar{T}_0^{(v)} + (m^{(v)})^{-\frac{1}{2}} \left(\frac{1}{m_H^{(v)}} A_\bullet^{(v)}(\bar{T}_{\infty,\epsilon}^{(v)}) - m_H^{-1} a(\tau) m^{(v)}\right) \\
&= (m^{(v)})^{\frac{1}{2}} \bar{T}_0^{(v)} + (m^{(v)})^{-\frac{1}{2}} \left(A_\bullet^{(v)}(\bar{T}_{\infty,\epsilon}^{(v)}) - m^{(v)} a(\tau)\right) m_H^{-1} \\
&\quad + (m^{(v)})^{-\frac{1}{2}} A_\bullet^{(v)}(\bar{T}_{\infty,\epsilon}^{(v)}) \left(\frac{1}{m_H^{(v)}} - \frac{1}{m_H}\right).
\end{aligned} \tag{9.34}$$

Now

$$(m^{(v)})^{-\frac{1}{2}} A_\bullet^{(v)}(\bar{T}_{\infty,\epsilon}^{(v)}) \left(\frac{1}{m_H^{(v)}} - \frac{1}{m_H}\right) = (m^{(v)})^{-1} A_\bullet^{(v)}(\bar{T}_{\infty,\epsilon}^{(v)}) (m^{(v)})^{\frac{1}{2}} \left(\frac{m_H - m_H^{(v)}}{m_H m_H^{(v)}}\right).$$

Theorem 9.3 implies that $\mathbb{P}\left((m^{(v)})^{-1} A_\bullet^{(v)}(\bar{T}_{\infty,\epsilon}^{(v)}) < a(\infty)\right) \to 1$ as $v \to \infty$ and condition (i) implies that $(m^{(v)})^{\frac{1}{2}} \left(\frac{m_H - m_H^{(v)}}{m_H m_H^{(v)}}\right) \to 0$ as $v \to \infty$. Thus,

$$(m^{(v)})^{-\frac{1}{2}} A_\bullet^{(v)}(\bar{T}_{\infty,\epsilon}^{(v)}) \left(\frac{1}{m_H^{(v)}} - \frac{1}{m_H}\right) = o_p(1),$$

where $o_p(1)$ denotes any random variable X_v satisfying $X_v \xrightarrow{\text{p}} 0$ as $v \to \infty$. Thus, using (9.33) and (9.34),

$$B_j^{(v)} = (m^{(v)})^{-\frac{1}{2}} \left[A_\bullet^{(v)}(\bar{T}_{\infty,\epsilon}^{(v)}) - m^{(v)} a(\tau)\right] m_H^{-1} r_j'(\xi_j^{(v)}) + o_p(1),$$

which, together with (9.31) and (9.32), gives

$$\begin{aligned}
&(m^{(v)})^{-\frac{1}{2}} \left[R_{\bullet,j}^{(v)}(\bar{T}_{\infty,\epsilon}^{(v)}) - m^{(v)} r_j(\tau)\right] \\
&\qquad = X_j(\tau) + (m^{(v)})^{-\frac{1}{2}} \left[A_\bullet^{(v)}(\bar{T}_{\infty,\epsilon}^{(v)}) - m^{(v)} a(\tau)\right] m_H^{-1} r_j'(\xi_j^{(v)}) + o_p(1).
\end{aligned}$$

For $j = 1, 2, \ldots, p+1$, we have $r_j'(\xi_j^{(v)}) \xrightarrow{\text{p}} r_j'(\tau)$ as $v \to \infty$, since the function $r_j'(t)$ is continuous and $\xi_j^{(v)} \xrightarrow{\text{p}} \tau$ as $v \to \infty$. Recall that $R_{\bullet,p+1}^{(v)}(t) = A_\bullet^{(v)}(t)$ and $r_{p+1}(t) = a(t)$. Then, a further application of Slutsky's theorem yields

$$(m^{(v)})^{-\frac{1}{2}} \boldsymbol{G} \left[\boldsymbol{R}_\bullet^{(v)}(\bar{T}_{\infty,\epsilon}^{(v)}) - m^{(v)} \boldsymbol{r}(\tau)\right] \xrightarrow{\text{D}} \boldsymbol{X}(\tau) \qquad \text{as } v \to \infty,$$

where

$$\boldsymbol{G} = \begin{pmatrix} \boldsymbol{I}_p & -m_H^{-1}\tilde{\boldsymbol{r}}'(\tau) \\ \boldsymbol{0}_p^T & 1 - m_H^{-1}a'(\tau) \end{pmatrix}.$$

Thus, $(m^{(v)})^{-\frac{1}{2}}\left[\boldsymbol{R}_\bullet^{(v)}(\bar{T}_{\infty,\epsilon}^{(v)}) - \boldsymbol{r}(\tau)\right] \xrightarrow{\mathrm{D}} \boldsymbol{G}^{-1}\boldsymbol{X}(\tau)$ as $v \to \infty$. It is easily checked that $\boldsymbol{G}^{-1} = \boldsymbol{H}$, where $\boldsymbol{H}$ is given by (9.28). Hence, $\boldsymbol{G}^{-1}\boldsymbol{X}(\tau) \sim \mathrm{N}(\boldsymbol{0}_{p+1}, \boldsymbol{HC}(\tau,\tau)\boldsymbol{H}^\top)$ and (9.29) follows. □

Armed with Theorem 9.6, we derive a central limit theorem for a vector of final outcome quantities defined on the epidemic $\mathcal{E}^{(v)}$, conditional upon a major outbreak. Note that this step is missing in Ball and Lyne [3]. Let $\boldsymbol{R}^{(v)} = (R_1^{(v)}, R_2^{(v)}, \dots, R_{p+1}^{(v)})^\top$ be final outcome quantities corresponding to $\boldsymbol{R}^{(v)}(t)$, but defined on $\mathcal{E}^{(v)}$ rather than $\hat{\mathcal{E}}^{(v)}(t)$. Thus, for example, $R_{p+1}^{(v)}$ is the weighted severity of $\mathcal{E}^{(v)}$. Again, the notation is slightly different from that in Section 5.3, where the weighted severity had its own notation.

Theorem 9.7 *Suppose that* $R_* > 1$ *and* $\lim_{v\to\infty}\sqrt{m^{(v)}}\left(\alpha_{\boldsymbol{n}}^{(v)} - \alpha_{\boldsymbol{n}}\right) = 0$, *for each* $\boldsymbol{n} \in \mathcal{N}$. *Then, as* $v \to \infty$, *conditional upon the event* $G^{(v)}$ *occurring, the law of* ${m^{(v)}}^{-\frac{1}{2}}\left(\boldsymbol{R}^{(v)} - m^{(v)}\boldsymbol{r}(\tau)\right)$ *converges to the multivariate normal distribution* $\mathrm{N}(\boldsymbol{0}_{p+1}, \boldsymbol{HC}(\tau,\tau)\boldsymbol{H}^\top)$.

Proof We construct a realisation of the final outcome of $\mathcal{E}^{(v)}$ from the process $\hat{\mathcal{E}}^{(v)}$ by setting $T_0^{(v)}$ and the tolerances of the initial infectives to zero, as in the proof of Theorem 9.4. With this change, $\boldsymbol{R}^{(v)} = \boldsymbol{R}_\bullet^{(v)}(\bar{T}_\infty^{(v)})$ and, for $t \geqslant 0$, $Z_\bullet^{(v)}(t)$ and $Z_E^{(v)}$ are the total number of households infected in the epidemics $\hat{\mathcal{E}}^{(v)}(t)$ and $\mathcal{E}^{(v)}$, respectively. Moreover, $Z_E^{(v)} = Z_\bullet^{(v)}(\bar{T}_\infty^{(v)})$. Let $S^{(v)} = \inf\{t : Z_\bullet^{(v)}(t) \geqslant \log m^{(v)}\}$, which is finite almost surely since $Z_\bullet^{(v)}(t) \xrightarrow{\text{a.s.}} m^{(v)}$ as $t \to \infty$. It follows from (9.6) and $Z_E^{(v)} = Z_\bullet^{(v)}(\bar{T}_\infty^{(v)})$ that $Z_E^{(v)} \geqslant \log m^{(v)}$, i.e. the event $G^{(v)}$ occurs, if and only if

$$\bar{T}_0^{(v)} + \frac{A_\bullet^{(v)}(t)}{N^{(v)}} > t \text{ for all } t \in [0, S^{(v)}).$$

Suppose that $G^{(v)}$ occurs. We continue the embedding construction after the population has been exposed to $S^{(v)}$ units of global infection, but resetting the tolerances and initial condition so that it starts again as follows. For an individual who is infected in $\hat{\mathcal{E}}^{(v)}(S^{(v)})$, whose original tolerance was L_{ij} say, their reset tolerance is $\check{L}_{ij} = \infty$. For an individual who is not infected in $\hat{\mathcal{E}}^{(v)}(S^{(v)})$, whose original tolerance was $L_{i'j'}$ say, their reset tolerance is $\check{L}_{i'j'} = L_{i'j'} - S^{(v)}$. We now consider the embedding construction among the population of $m^{(v)}$ households, with the reset tolerances and the population initially being exposed to $\check{T}_0^{(v)} = A_\bullet^{(v)}(S^{(v)}) - S^{(v)}$ units of global infection. The lack-of-memory property of the exponential distribution ensures that the reset tolerances have the required distribution. Let $\bar{\check{T}}_0^{(v)} = \check{T}_0^{(v)}/N^{(v)}$ and $\bar{\check{T}}_\infty^{(v)}$ be given by the right-hand side of (9.6) with $\bar{T}_0^{(v)}$ replaced by $\bar{\check{T}}_0^{(v)}$. Then, $\boldsymbol{R}_\bullet^{(v)}(\bar{\check{T}}_\infty^{(v)}) \stackrel{D}{=} \boldsymbol{R}^{(v)} \mid G^{(v)}$.

Note that $\breve{T}_0^{(v)}$ is bounded above by the sum of the infectious periods of *all* individuals in the $\lceil \log m^{(v)} \rceil$ households infected in $\hat{\mathcal{E}}^{(v)}(T'^{(v)})$. Since $\mu_I^{(i)} < \infty$ for $i \in \mathcal{J}$, a simple application of Markov's inequality yields $(m^{(v)})^{-\frac{1}{2}}\breve{T}_0^{(v)} \xrightarrow{\text{P}} 0$ as $v \to \infty$. Moreover, this convergence also holds conditional upon the event $G^{(v)}$ occurring, since $\mathbb{P}(G^{(v)}) \to 1 - p_{\text{ext}}$ as $v \to \infty$ and $1 - p_{\text{ext}} > 0$ as $R_* > 1$. Thus condition (ii) of Theorem 9.6 is satisfied and Theorem 9.6 can be applied to the reset embedding process. (A similar argument to the above, using (9.27), shows that the change to the tolerances in the $\lceil \log m^{(v)} \rceil$ households infected by $\hat{\mathcal{E}}^{(v)}(S^{(v)})$ and to the contributions of those households to $\{\boldsymbol{R}_\bullet^{(v)}(t)\}$ do not affect the conclusions of Theorems 9.5 and 9.6.) Therefore, setting $\epsilon = \tau/2$ in Theorem 9.6 yields, in an obvious notation,

$$(m^{(v)})^{-\frac{1}{2}} \left(\boldsymbol{R}_\bullet^{(v)}(\bar{\bar{T}}_{\infty,\tau/2}^{(v)}) - m^{(v)}\boldsymbol{r}(\tau) \right) \xrightarrow{\text{D}} \text{N}(\boldsymbol{0}_{p+1}, \boldsymbol{HC}(\tau,\tau)\boldsymbol{H}^\top) \qquad \text{as } v \to \infty.$$

The theorem follows since Theorem 9.4 implies that $\mathbb{P}(\bar{\bar{T}}_\infty^{(v)} > \tau/2 | G^{(v)}) \to 1$ as $v \to \infty$. □

9.2.4 Proof of Theorem 9.5.

We show that the finite-dimensional distributions of $(m^{(v)})^{-\frac{1}{2}} \left(\boldsymbol{R}_{\bullet,T}^{(v)} - \mathbb{E}[\boldsymbol{R}_{\bullet,T}^{(v)}] \right)$ converge to those of $\boldsymbol{X}_T$ and that the sequence $(m^{(v)})^{-\frac{1}{2}} \left(\boldsymbol{R}_{\bullet,T}^{(v)} - \mathbb{E}[\boldsymbol{R}_{\bullet,T}^{(v)}] \right)$ $(v = 1, 2, \dots)$ is asymptotically tight. The required weak convergence then follows by van der Vaart and Wellner [11], Theorem 1.5.4.

We use the Cramér-Wold device to show convergence of the finite-dimensional distributions. For $i = 1, 2, \dots, (p+1)$, let $q_i \in \mathbb{N}$ and, for $j = 1, 2, \dots, q_i$, let $\lambda_{ij} \in \mathbb{R}$ and $t_{ij} \geqslant 0$. For $v = 1, 2, \dots$, let

$$\begin{aligned} R_\lambda^{(v)} &= \frac{1}{\sqrt{m^{(v)}}} \sum_{\boldsymbol{n} \in \mathcal{N}} \sum_{k=1}^{m_{\boldsymbol{n}}^{(v)}} \sum_{i=1}^{p+1} \sum_{j=1}^{q_i} \lambda_{ij} \left(R_i^{(\boldsymbol{n},k)}(t_{ij}) - \mathbb{E}[R_i^{(\boldsymbol{n},k)}(t_{ij})] \right) \\ &= \sum_{\boldsymbol{n} \in \mathcal{N}} \sqrt{\alpha_{\boldsymbol{n}}^{(v)}} Y_{\boldsymbol{n}}^{(v)}, \end{aligned}$$

where

$$\begin{aligned} Y_{\boldsymbol{n}}^{(v)} &= \frac{1}{\sqrt{m_{\boldsymbol{n}}^{(v)}}} \sum_{k=1}^{m_{\boldsymbol{n}}^{(v)}} \left\{ \sum_{i=1}^{p+1} \sum_{j=1}^{q_i} \lambda_{ij} \left(R_i^{(\boldsymbol{n},k)}(t_{ij}) - \mathbb{E}[R_i^{(\boldsymbol{n},k)}(t_{ij})] \right) \right\} \\ &= \frac{1}{\sqrt{m_{\boldsymbol{n}}^{(v)}}} \sum_{k=1}^{m_{\boldsymbol{n}}^{(v)}} W_{\boldsymbol{n},k} \qquad \text{say.} \end{aligned}$$

Now $W_{\boldsymbol{n},k}$ $(k = 1, 2, \dots)$ are independent and identically distributed, with zero mean, so by the central limit theorem, $Y_{\boldsymbol{n}}^{(v)} \xrightarrow{\text{D}} \text{N}(0, \sigma_{\boldsymbol{n}}^2)$ as $n \to \infty$, where

$$\sigma_{\boldsymbol{n}}^2 = \sum_{i=1}^{p+1} \sum_{j=1}^{q_i} \sum_{a=1}^{p+1} \sum_{b=1}^{q_a} \lambda_{ij}\lambda_{ab} \text{cov}\left(R_i^{(\boldsymbol{n},1)}(t_{ij}), R_a^{(\boldsymbol{n},1)}(t_{ab}) \right).$$

Further, $(Y_{\boldsymbol{n}}^{(v)})$ $(\boldsymbol{n} \in \mathcal{N})$ are independent and $\alpha_{\boldsymbol{n}}^{(v)} \to \alpha_{\boldsymbol{n}}$ as $v \to \infty$, so

$$R_\lambda^{(v)} \xrightarrow{\mathrm{D}} \mathrm{N}\left(0, \sum_{\boldsymbol{n}\in\mathcal{N}} \alpha_{\boldsymbol{n}} \sigma_{\boldsymbol{n}}^2\right) \qquad \text{as } v \to \infty.$$

Recalling the covariance function of $\boldsymbol{X}_T$ in Theorem 9.5, we also have that

$$\sum_{i=1}^{p+1} \sum_{j=1}^{q_i} \lambda_{ij} X_i(t_{ij}) \sim \mathrm{N}\left(0, \sum_{\boldsymbol{n}\in\mathcal{N}} \alpha_{\boldsymbol{n}} \sigma_{\boldsymbol{n}}^2\right).$$

Hence,

$$R_\lambda^{(v)} \xrightarrow{\mathrm{D}} \sum_{i=1}^{p+1} \sum_{j=1}^{q_i} \lambda_{ij} X_i(t_{ij}) \qquad \text{as } v \to \infty. \tag{9.35}$$

Thus, by the Cramér-Wold device, the finite-dimensional distributions of $(m^{(v)})^{-\frac{1}{2}}\left(\boldsymbol{R}_{\bullet,T}^{(v)} - \mathbb{E}[\boldsymbol{R}_{\bullet,T}^{(v)}]\right)$ converge to those of $\boldsymbol{X}_T$, since (9.35) holds for any choice of q_i and (λ_{ij}, t_{ij}) $(i = 1, 2, \dots, p+1, j = 1, 2, \dots, q_i)$.

Turning to asymptotic tightness, by van der Vaart and Wellner [11], Lemma 1.4.3, it is sufficient to show this for each component of $\boldsymbol{R}$ separately. Note that $\{R_{p+1}^{(\boldsymbol{n})}(t)\} = \{A^{(\boldsymbol{n})}(t)\}$ satisfies the condition (9.27) by Minkowski's inequality. Let R denote a typical component of $\boldsymbol{R}$. For $\epsilon > 0$, the bracketing number $N_{[]}^{(v)}(\epsilon, T)$ is defined as the minimum number of sets N_ϵ in a partition $[0, T] = \bigcup_{i=1}^{N_\epsilon} \mathcal{A}_{\epsilon j}^{(v)}$ such that, for each set $\mathcal{A}_{\epsilon j}^{(v)}$, we have

$$\sum_{\boldsymbol{n}\in\mathcal{N}} \sum_{k=1}^{m_{\boldsymbol{n}}^{(v)}} \mathbb{E}\left[\max_{t,s\in\mathcal{A}_{\epsilon j}^{(v)}} \left|\frac{1}{\sqrt{m^{(v)}}}\left(R^{(\boldsymbol{n},k)}(t) - R^{(\boldsymbol{n},k)}(s)\right)\right|^2\right] \leqslant \epsilon^2. \tag{9.36}$$

Also, for $T > 0$ and $f : [0, \infty) \to \mathbb{R}$, let

$$\|f\|_T = \sup_{0\leqslant t\leqslant T} |f(t)|.$$

By van der Vaart and Wellner [11], Theorem 2.11.9, $(m^{(v)})^{-\frac{1}{2}}\left(R_T^{(v)} - \mathbb{E}[R_T^{(v)}]\right)$ $(v = 1, 2, \dots)$ is asymptotically tight if the following three conditions hold.

(i) For any $d > 0$, we have

$$\sum_{\boldsymbol{n}\in\mathcal{N}} \sum_{k=1}^{m_{\boldsymbol{n}}^{(v)}} \mathbb{E}\left[\left\|\frac{1}{\sqrt{m^{(v)}}} R^{(\boldsymbol{n},k)}\right\|_T \mathbb{1}_{\left\{\left\|\frac{1}{\sqrt{m^{(v)}}} R^{(\boldsymbol{n},k)}\right\|_T > d\right\}}\right] \to 0 \text{ as } v \to \infty.$$

(ii) For any sequence (δ_v) satisfying $\delta_v \downarrow 0$ as $v \to \infty$, we have

$$\sup_{s,t\in[0,T]:|s-t|<\delta_v} \sum_{\boldsymbol{n}\in\mathcal{N}} \sum_{k=1}^{m_{\boldsymbol{n}}^{(v)}} \frac{1}{m^{(v)}} \mathbb{E}\left[\left(R^{(\boldsymbol{n},k)}(t) - R^{(\boldsymbol{n},k)}(s)\right)^2\right] \to 0 \text{ as } v \to \infty.$$

(iii) For any sequence (δ_v) satisfying $\delta_v \downarrow 0$ as $v \to \infty$, we have

$$\int_0^{\delta_v} \sqrt{\log N_{[]}^{(v)}(\epsilon, T)}\, \mathrm{d}\epsilon \to 0 \text{ as } v \to \infty.$$

To show condition (i), for $\boldsymbol{n} \in \mathcal{N}$, let $X_{\boldsymbol{n}} = \frac{1}{\sqrt{m^{(v)}}} \sup_{t\geqslant 0} \left|R^{(\boldsymbol{n})}(t)\right|$ and note that (9.27) implies $(m^{(v)})^{1+\frac{\zeta}{2}} \mathbb{E}[X_{\boldsymbol{n}}^{2+\zeta}] < \infty$. Now

$$\sum_{\boldsymbol{n}\in\mathcal{N}} \sum_{k=1}^{m_{\boldsymbol{n}}^{(v)}} \mathbb{E}\left[\left\|\frac{1}{\sqrt{m^{(v)}}} R^{(\boldsymbol{n},k)}(t)\right\|_T \mathbb{1}_{\left\{\left\|\frac{1}{\sqrt{m^{(v)}}} R^{(\boldsymbol{n},k)}(t)\right\|_T > d\right\}}\right] \leqslant \sum_{\boldsymbol{n}\in\mathcal{N}} m_{\boldsymbol{n}}^{(v)} \mathbb{E}\left[X_{\boldsymbol{n}} \mathbb{1}_{\{X_{\boldsymbol{n}}>d\}}\right],$$

since $\{R^{(\boldsymbol{n},k)}(t)\}$ $(k = 1, 2, \ldots, m_{\boldsymbol{n}}^{(v)})$ are identically distributed. Arguing as in a proof of Markov's inequality yields

$$\mathbb{E}\left[X_{\boldsymbol{n}} \mathbb{1}_{\{X_{\boldsymbol{n}}>d\}}\right] = d^{-(1+\zeta)} \mathbb{E}\left[X_{\boldsymbol{n}} \mathbb{1}_{\{X_{\boldsymbol{n}}>d\}} d^{1+\zeta}\right] \leqslant d^{-(1+\zeta)} \mathbb{E}\left[X_{\boldsymbol{n}}^{2+\zeta}\right].$$

Hence,

$$\begin{aligned}
\sum_{\boldsymbol{n}\in\mathcal{N}} m_{\boldsymbol{n}}^{(v)} \mathbb{E}\left[X_{\boldsymbol{n}} \mathbb{1}_{\{X_{\boldsymbol{n}}>d\}}\right] &\leqslant d^{-(1+\zeta)} \sum_{\boldsymbol{n}\in\mathcal{N}} \alpha_{\boldsymbol{n}}^{(v)} m^{(v)} \mathbb{E}\left[X_{\boldsymbol{n}}^{2+\zeta}\right] \\
&\leqslant d^{-(1+\zeta)} (m^{(v)})^{-\frac{\zeta}{2}} \sum_{\boldsymbol{n}\in\mathcal{N}} \alpha_{\boldsymbol{n}}^{(v)} (m^{(v)})^{1+\frac{\zeta}{2}} \mathbb{E}\left[X_{\boldsymbol{n}}^{2+\zeta}\right] \\
&\to 0 \text{ as } v \to \infty,
\end{aligned}$$

so, condition (i) is satisfied.

Turning next to condition (ii), let (δ_v) be any sequence satisfying $\delta_v \downarrow 0$ as $v \to \infty$ and, without loss of generality, assume that $0 < s < t$. For $\boldsymbol{n} \in \mathcal{N}$, consider the process $\tilde{\mathcal{E}}_{\boldsymbol{n}} = \{\tilde{\mathcal{E}}_{\boldsymbol{n}}(t) : t \geqslant 0\}$ defined in Section 9.2.1. We construct the tolerances (L_{ij}s) as follows. Let ξ_{ij} $(i \in \mathcal{J}, j = 1, 2, \ldots, n_i)$ be independent Poisson processes, where ξ_{ij} has rate κ_i. Then, for $i \in \mathcal{J}$ and $j = 1, 2, \ldots, n_i$, we set L_{ij} to be the time of the first point in ξ_{ij}. Let $F_{\boldsymbol{n}}(s, t)$ be the event that at least one of these ξ_{ij}s has a point in the interval $(s, t]$. Then, since $R^{(\boldsymbol{n})}(t)$ only jumps when $t = L_{ij}$ for some (i, j), we have

$$\mathbb{E}\left[\left(R^{(\boldsymbol{n})}(t) - R^{(\boldsymbol{n})}(s)\right)^2\right] \leqslant 4\mathbb{E}\left[\left(\sup_{t\geqslant 0}\left|R^{(\boldsymbol{n})}(t)\right|\right)^2 \mathbb{1}_{\{F_{\boldsymbol{n}}(s,t)\}}\right]$$

$$
\begin{aligned}
&= 4\mathbb{E}\left[\left(\sup_{t\geqslant 0}\left|R^{(\boldsymbol{n})}(t)\right|\right)^2\right]\left(1-\exp\left(-\sum_{i=1}^{J} n_i\kappa_i(t-s)\right)\right) \\
&\leqslant 4\mathbb{E}\left[\left(\sup_{t\geqslant 0}\left|R^{(\boldsymbol{n})}(t)\right|\right)^2\right]\sum_{i=1}^{J} n_i\kappa_i(t-s) \\
&= (t-s)K_{\boldsymbol{n}}, \text{ say,}
\end{aligned}
$$

where $K_{\boldsymbol{n}} < \infty$ by (9.27). It then follows easily that condition (ii) is satisfied. Finally, note that (9.36) is satisfied if $[0,T]$ is partitioned into intervals of length $L_\epsilon = \frac{\epsilon^2}{\sum_{\boldsymbol{n}\in\mathcal{N}} K_{\boldsymbol{n}}}$ (the final interval may have a shorter length). Therefore, $N_{[]}^{(v)}(\epsilon, T) \leqslant \frac{c}{\epsilon^2}$, where $c = 2T\sum_{\boldsymbol{n}\in\mathcal{N}} K_{\boldsymbol{n}}$. It follows that

$$
\begin{aligned}
\int_0^{\delta_v}\sqrt{\log N_{[]}^{(v)}(\epsilon,T)}\,\mathrm{d}\epsilon &\leqslant \int_0^{\delta_v}\sqrt{\log\left(\frac{c}{\epsilon^2}\right)}\,\mathrm{d}\epsilon \\
&= \frac{\sqrt{c}}{2}\int_{\log\left(\frac{c}{\delta_v^2}\right)}^{\infty}\sqrt{u}\exp\left(-\frac{u}{2}\right)\,\mathrm{d}u \to 0 \text{ as } v\to\infty,
\end{aligned}
$$

so condition (iii) is satisfied.

10 Expressions for properties of the epidemics $\mathcal{E}_{\boldsymbol{n},i}(\boldsymbol{\Lambda}^{\boldsymbol{L}}(\boldsymbol{n}))$ and $\tilde{\mathcal{E}}_{\boldsymbol{n}}(\boldsymbol{\Lambda}^{\boldsymbol{L}}(\boldsymbol{n}), \boldsymbol{\pi})$

We give expressions for $\mu_{\boldsymbol{n},i,j}(\Lambda^L(\boldsymbol{n}))$, $\tilde{\mu}_{\boldsymbol{n},j}(\Lambda^L(\boldsymbol{n}),\boldsymbol{\pi})$ and $\phi_{\boldsymbol{n},i}(\boldsymbol{\theta})$ in terms of multivariate Gontcharoff polynomials, which were introduced by Lefèvre and Picard [6]. We give also a similar expression for the Laplace transform of $\boldsymbol{R}^{(\boldsymbol{n})}(t)$ for final outcome vectors that are additive over individuals. We first give some notation that is used throughout this section.

For vectors $\boldsymbol{x} = (x_1, x_2, \ldots, x_J), \boldsymbol{y} = (y_1, y_2, \ldots, y_J) \in \mathbb{R}^J$, $\boldsymbol{x}^{\boldsymbol{y}}$ denotes the product $\prod_{k=1}^J x_k^{y_k}$, and $\boldsymbol{x} \leqslant \boldsymbol{y}$ means $x_k \leqslant y_k$ for all $k = 1, 2, \ldots, J$. For $\boldsymbol{k} = (k_1, k_2, \ldots, k_J)$ and $\boldsymbol{n} = (n_1, n_2, \ldots, n_J) \in \mathbb{Z}_+^J$, $\boldsymbol{n}_{[\boldsymbol{k}]} = \prod_{i=1}^J n_{i[k_i]}$, where $n_{[k]} = n(n-1)\ldots(n-k+1)$ is a falling factorial with the convention $n_{[0]} = 1$. The row vectors consisting of J zeros and J ones are denoted by $\mathbf{0}$ and $\mathbf{1}$, respectively. Finally, $\sum_{\boldsymbol{k}=\boldsymbol{i}}^{\boldsymbol{n}}$ denotes $\sum_{k_1=i_1}^{n_1}\sum_{k_2=i_2}^{n_2}\cdots\sum_{k_J=i_J}^{n_J}$.

Let $\boldsymbol{U} = \left\{\boldsymbol{u_i} : \boldsymbol{i} \in \mathbb{Z}_+^J\right\}$ be a collection of vectors in $\mathbb{R}^J$. The family of multivariate Gontcharoff polynomials associated with $\boldsymbol{U}$, i.e. $G_{\boldsymbol{i}}(\boldsymbol{x}|\boldsymbol{U})$ ($\boldsymbol{i} \in \mathbb{Z}_+^J$), where $\boldsymbol{x} = (x_1, x_2, \ldots, x_J)$, is defined recursively by

$$
\sum_{\boldsymbol{i}=\boldsymbol{0}}^{\boldsymbol{k}} \boldsymbol{k}_{[\boldsymbol{i}]}\boldsymbol{u}_{\boldsymbol{i}}^{\boldsymbol{k}-\boldsymbol{i}} G_{\boldsymbol{i}}(\boldsymbol{x}|\boldsymbol{U}) = \boldsymbol{x}^{\boldsymbol{k}} \qquad (\boldsymbol{k} \in \mathbb{Z}_+^J).
$$

For $\boldsymbol{i},\boldsymbol{j} \in \mathbb{Z}_+^J$, let $G_{\boldsymbol{i}}^{(\boldsymbol{j})}(\boldsymbol{x}|\boldsymbol{U})$ denote the partial derivative of $G_{\boldsymbol{i}}(\boldsymbol{x}|\boldsymbol{U})$ of order $j_1, j_2, \ldots, j_J$ in $x_1, x_2, \ldots, x_J$, respectively. Then, for $\mathbf{0} \leqslant \boldsymbol{j} \leqslant \boldsymbol{i}$,

$$G_{\boldsymbol{i}}^{(\boldsymbol{j})}(\boldsymbol{x}|\boldsymbol{U}) = G_{\boldsymbol{i}-\boldsymbol{j}}(\boldsymbol{x})|E^{\boldsymbol{j}}\boldsymbol{U}),$$

where $E^{\boldsymbol{j}}\boldsymbol{U} = \left\{\boldsymbol{u}_{\boldsymbol{i}+\boldsymbol{j}} : \boldsymbol{i} \in \mathbb{Z}_+^J\right\}$ (Lefèvre and Picard [6], Property 4.4).

For $\boldsymbol{n} \in \mathcal{N}$ and $\boldsymbol{k} \in \mathbb{Z}_+^J$, let $\boldsymbol{q}_{\boldsymbol{k}}(\boldsymbol{n}) = \left(q_{\boldsymbol{k}}^{(1)}(\boldsymbol{n}), q_{\boldsymbol{k}}^{(2)}(\boldsymbol{n}), \ldots, q_{\boldsymbol{k}}^{(J)}(\boldsymbol{n})\right)$, where

$$q_{\boldsymbol{k}}^{(i)}(\boldsymbol{n}) = \phi_{I^{(i)}}\left(\lambda_{i1}^L(\boldsymbol{n})k_1 + \lambda_{i2}^L(\boldsymbol{n})k_2 + \cdots + \lambda_{iJ}^L(\boldsymbol{n})k_J\right)$$

with $\phi_{I^{(i)}}(\theta) = \mathbb{E}[\exp(-\theta I^{(i)})]$ $(\theta \geqslant 0)$. Note that $q_{\boldsymbol{k}}^{(i)}(\boldsymbol{n})$ is the probability that a type-i infective in a category-$\boldsymbol{n}$ household does not contact anyone in a given set of size $k_1 + k_2 + \cdots + k_J$ consisting of k_j type-j individuals $(j \in \mathcal{J})$.

Recall that, for $i \in \mathcal{J}$, $\boldsymbol{e}_i$ denotes the unit vector of length J, in which the ith element is 1 and all other elements are 0. The following results follow immediately from the first display in the proof of Ball [1], Theorem 4.1. For $i, j \in \mathcal{J}$ and $\boldsymbol{n} \in \mathcal{N}_i$,

$$\mu_{\boldsymbol{n},i,j}(\Lambda^L(\boldsymbol{n})) = n_j - \sum_{\boldsymbol{k}=\mathbf{0}}^{\boldsymbol{n}-\boldsymbol{e}_i} (\boldsymbol{n} - \boldsymbol{e}_i)_{[\boldsymbol{k}]} \left(\boldsymbol{q}_{\boldsymbol{k}}(\boldsymbol{n})\right)^{\boldsymbol{n}-\boldsymbol{k}} G_{\boldsymbol{k}}^{(\boldsymbol{e}_j)}(\mathbf{1}|\boldsymbol{U}(\boldsymbol{n})), \tag{10.1}$$

where $\boldsymbol{U}(\boldsymbol{n}) = \left\{\boldsymbol{u}_{\boldsymbol{i}}(\boldsymbol{n}) : \boldsymbol{i} \in \mathbb{Z}_+^J\right\}$ with $\boldsymbol{u}_{\boldsymbol{i}}(\boldsymbol{n}) = \boldsymbol{q}_{\boldsymbol{i}}(\boldsymbol{n})$ $(\boldsymbol{i} \in \mathbb{Z}_+^J)$. For $\boldsymbol{n} \in \mathcal{N}$, $j \in \mathcal{J}$ and $\boldsymbol{\pi} \in [0,1]^J$,

$$\tilde{\mu}_{\boldsymbol{n},j}(\Lambda^L(\boldsymbol{n}), \boldsymbol{\pi}) = n_j - \sum_{\boldsymbol{k}=\mathbf{0}}^{\boldsymbol{n}} \boldsymbol{n}_{[\boldsymbol{k}]} \left(\boldsymbol{q}_{\boldsymbol{k}}(\boldsymbol{n})\right)^{\boldsymbol{n}-\boldsymbol{k}} \boldsymbol{\pi}^{\boldsymbol{k}} G_{\boldsymbol{k}}^{(\boldsymbol{e}_j)}(\mathbf{1}|\boldsymbol{U}(\boldsymbol{n})). \tag{10.2}$$

Recall from Section 4.4 that $\phi_{\boldsymbol{n},i}(\boldsymbol{\theta})$ $(\boldsymbol{\theta} = (\theta_1, \theta_2, \ldots, \theta_J)^\top \in \mathbb{R}_+^J)$ is the joint Laplace transform of $(A_1, A_2, \ldots, A_J)$, defined in Section 3.1 for the epidemic $\mathcal{E}_{\boldsymbol{n},i}(\Lambda^L(\boldsymbol{n}))$. For $\boldsymbol{n} \in \mathcal{N}$ and $\boldsymbol{\theta} \in \mathbb{R}_+^p$, let

$$\boldsymbol{q}_{\boldsymbol{n},\boldsymbol{k}}(\boldsymbol{\theta}) = \left(q_{\boldsymbol{n},\boldsymbol{k}}^{(1)}(\boldsymbol{\theta}), q_{\boldsymbol{n},\boldsymbol{k}}^{(2)}(\boldsymbol{\theta}), \ldots, q_{\boldsymbol{n},\boldsymbol{k}}^{(J)}(\boldsymbol{\theta})\right) \qquad (\boldsymbol{k} \geqslant \mathbf{0}),$$

where, for $i \in \mathcal{J}$,

$$q_{\boldsymbol{n},\boldsymbol{k}}^{(i)}(\boldsymbol{\theta}) = \phi_{I^{(i)}}(\theta_i + \lambda_{i1}^L(\boldsymbol{n})k_1 + \lambda_{i2}^L(\boldsymbol{n})k_2 + \cdots + \lambda_{iJ}^L(\boldsymbol{n})k_J).$$

Then it follows from Picard and Lefèvre [7], Proposition 4.3, or Ball [1], Theorem 4.2, that for $i \in \mathcal{J}$ and $\boldsymbol{n} \in \mathcal{N}_i$,

$$\phi_{\boldsymbol{n}.i}(\boldsymbol{\theta}) = \sum_{\boldsymbol{k}=\mathbf{0}}^{\boldsymbol{n}-\boldsymbol{e}_i} (\boldsymbol{n} - \boldsymbol{e}_i)_{[\boldsymbol{k}]} \left(q_{\boldsymbol{n},\boldsymbol{k}}(\boldsymbol{\theta})\right)^{\boldsymbol{n}-\boldsymbol{k}} G_{\boldsymbol{k}}(\mathbf{1}|\boldsymbol{U}(\boldsymbol{n},\boldsymbol{\theta})) \qquad (\boldsymbol{\theta} \in \mathbb{R}_+^p), \tag{10.3}$$

where $\boldsymbol{U}(\boldsymbol{n},\boldsymbol{\theta}) = \{\boldsymbol{u}_{\boldsymbol{i}}(\boldsymbol{n},\boldsymbol{\theta}) : \boldsymbol{i} \in \mathbb{Z}_+^J\}$ with $\boldsymbol{u}_{\boldsymbol{i}}(\boldsymbol{n},\boldsymbol{\theta}) = \boldsymbol{q}_{\boldsymbol{n},\boldsymbol{i}}(\boldsymbol{\theta})$ $(\boldsymbol{i} \in \mathbb{Z}_+^J)$.

For $\boldsymbol{n} \in \mathcal{N}$, consider the final outcome vector process $\{\boldsymbol{R}^{(\boldsymbol{n})}(t)\} = \{(R_1^{(\boldsymbol{n})}(t), R_2^{(\boldsymbol{n})}(t), \ldots, R_p^{(\boldsymbol{n})}(t))^\top : t \geqslant 0\}$ defined on the process $\tilde{\mathcal{E}}_{\boldsymbol{n}} = \{\tilde{\mathcal{E}}_{\boldsymbol{n}}(t) : t \geqslant 0\}$. We assume that $\{\boldsymbol{R}^{(\boldsymbol{n})}(t)\}$ is additive over individuals in the household; more precisely, if the individuals in the household are labelled (i,j) $(i \in \mathcal{J}, j = 1, 2, \ldots, n_i)$, as in Section 9.2.1, then

$$\boldsymbol{R}^{(\boldsymbol{n})}(t) = \sum_{i=1}^{J} \sum_{j=1}^{n_i} \boldsymbol{R}_{ij}^{(\boldsymbol{n})} \mathbb{1}_{\{\text{individual } (i,j) \text{ is infected in } \tilde{\mathcal{E}}_{\boldsymbol{n}}(t)\}} \qquad (t \geqslant 0),$$

where $\boldsymbol{R}_{ij}^{(\boldsymbol{n})}$ $(i \in \mathcal{J}, j = 1, 2, \ldots, n_i)$ are independent, with $\boldsymbol{R}_{i1}^{(\boldsymbol{n})}, \boldsymbol{R}_{i2}^{(\boldsymbol{n})}, \ldots, \boldsymbol{R}_{in_i}^{(\boldsymbol{n})}$ being identically distributed, for each $i \in \mathcal{J}$. The elements of $\boldsymbol{R}_{ij}^{(\boldsymbol{n})}$ may be dependent and may also depend on the infectious period I_{ij} of individual (i,j). We assume that $\boldsymbol{R}_{ij}^{(\boldsymbol{n})}$ is independent of the event $\{(i,j)$ is infected in $\tilde{\mathcal{E}}_{\boldsymbol{n}}(t)\}$, so

$$\boldsymbol{r}_{\boldsymbol{n}}(t) = \mathbb{E}\left[\boldsymbol{R}^{(\boldsymbol{n})}(t)\right] = \sum_{i=1}^{J} \tilde{\mu}_{\boldsymbol{n},j}(\Lambda^L(\boldsymbol{n}), \mathrm{e}^{-t\boldsymbol{\kappa}}) \mathbb{E}\left[\boldsymbol{R}_{i1}^{(\boldsymbol{n})}\right] \qquad (\boldsymbol{n} \in \mathcal{N}, t \geqslant 0),$$

since $\tilde{\mathcal{E}}_{\boldsymbol{n}}(t)$ is a realisation of the epidemic $\tilde{\mathcal{E}}_{\boldsymbol{n}}(\Lambda^L(\boldsymbol{n}), \mathrm{e}^{-t\boldsymbol{\kappa}})$ (see Section 9.2.1).

Let $\boldsymbol{\theta} = (\theta_1, \theta_2, \ldots, \theta_p)^\top$ and, for $t \geqslant 0$, let

$$\tilde{\phi}_{\boldsymbol{n}}(\boldsymbol{\theta}, t) = \mathbb{E}\left[\exp\left(-\boldsymbol{\theta}^\top \boldsymbol{R}^{(\boldsymbol{n})}(t)\right)\right] \qquad (\boldsymbol{\theta} \in \mathbb{R}_+^p)$$

be the Laplace transform of $\boldsymbol{R}^{(\boldsymbol{n})}(t)$. We give below an expression for $\tilde{\phi}_{\boldsymbol{n}}(\boldsymbol{\theta}, t)$, suitable differentiation of which enables the covariance matrix $\boldsymbol{C}^{(\boldsymbol{n})}(t)$ (see (5.8)) to be calculated. (The weighted severity $A^{(\boldsymbol{n})}(t)$ is a final outcome quantity that is additive over individuals.)

For $\boldsymbol{n} \in \mathcal{N}$ and $\boldsymbol{\theta} \in \mathbb{R}_+^p$, let

$$\tilde{\boldsymbol{q}}_{\boldsymbol{n},\boldsymbol{k}}(\boldsymbol{\theta}) = \left(\tilde{q}_{\boldsymbol{n},\boldsymbol{k}}^{(1)}(\boldsymbol{\theta}), \tilde{q}_{\boldsymbol{n},\boldsymbol{k}}^{(2)}(\boldsymbol{\theta}), \ldots, \tilde{q}_{\boldsymbol{n},\boldsymbol{k}}^{(J)}(\boldsymbol{\theta})\right) \qquad (\boldsymbol{k} \geqslant \boldsymbol{0}),$$

where, for $i \in \mathcal{J}$,

$$\tilde{q}_{\boldsymbol{n},\boldsymbol{k}}^{(i)}(\boldsymbol{\theta}) = \mathbb{E}\left[\exp\left(-\boldsymbol{\theta}^\top \boldsymbol{R}_{i1} + \sum_{j=1}^{J} \lambda_{ij}^L(\boldsymbol{n}) k_j I_{i1}\right)\right].$$

Then, using Ball [1], Theorem 4.2, for $\boldsymbol{n} \in \mathcal{N}$ and $t \geqslant 0$,

$$\tilde{\phi}_{\boldsymbol{n}}(\boldsymbol{\theta}, t) = \sum_{\boldsymbol{k}=\boldsymbol{0}}^{\boldsymbol{n}} \boldsymbol{n}_{[\boldsymbol{k}]} \left(\tilde{\boldsymbol{q}}_{\boldsymbol{n},\boldsymbol{k}}(\boldsymbol{\theta})\right)^{\boldsymbol{n}-\boldsymbol{k}} \mathrm{e}^{-t\boldsymbol{k}\boldsymbol{\kappa}^\top} G_{\boldsymbol{k}}(\boldsymbol{1} | \tilde{\boldsymbol{U}}(\boldsymbol{n}, \boldsymbol{\theta})) \qquad (\boldsymbol{\theta} \in \mathbb{R}_+^p),$$

where $\tilde{\boldsymbol{U}}(\boldsymbol{n}, \boldsymbol{\theta}) = \{\tilde{\boldsymbol{u}}_{\boldsymbol{i}}(\boldsymbol{n}, \boldsymbol{\theta}) : \boldsymbol{i} \in \mathbb{Z}_+^J\}$ with $\tilde{\boldsymbol{u}}_{\boldsymbol{i}}(\boldsymbol{n}, \boldsymbol{\theta}) = \tilde{\boldsymbol{q}}_{\boldsymbol{n},\boldsymbol{i}}(\boldsymbol{\theta})$ $(\boldsymbol{i} \in \mathbb{Z}_+^J)$.

References

[1] Ball F (2019) Susceptibility sets and the final outcome of collective Reed-Frost epidemics. Methodol Comput Appl Probab 21(2):401-421

[2] Ball F, Britton, T (2005) An epidemic model with exposure-dependent severities. J Appl Probab 42(4):932-949

[3] Ball F, Lyne OD (2001) Stochastic multitype SIR epidemics among a population partitioned into households. Adv Appl Probab 33(1):99-123

[4] Ball F, Mollison D, Scalia-Tomba G (1997) Epidemics with two levels of mixing. Ann Appl Probab 7(1):46-89

[5] Britton T, Janson S, Martin-Löf A (2007) Graphs with specified degree distributions, simple epidemics, and local vaccination strategies. Adv Appl Probab 39(4):922-948

[6] Lefèvre C, Picard P (1990) A non-standard family of polynomials and the final size distribution of Reed-Frost epidemic processes. Adv Appl Probab 22(1):25-48

[7] Picard P, Lefèvre C (1990) A unified analysis of the final size and severity distribution in collective Reed-Frost epidemic processes. Adv Appl Probab 22(2):269–294

[8] Scalia-Tomba G (1985) Asymptotic final-size distribution for some chain-binomial processes. Adv Appl Probab 17(3):477-495

[9] Scalia-Tomba G (1990) On the asymptotic final size distribution of epidemics in heterogeneous populations. In Stochastic Processes in Epidemic Theory (Lecture Notes Biomath 86), eds Gabriel J-P, Lefèvre C, Picard P, Springer, Berlin,pp. 189-196

[10] Sellke T (1983) On the asymptotic distribution of the size of a stochastic epidemic. J Appl Probab 20(2):390-394

[11] van der Vaart AW, Wellner JA (1996) Weak Convergence and Empirical Processes, Springer, New York

[12] Whittle P (1955) The outcome of a stochastic epidemic — a note on Bailey's paper. Biometrika 42(1-2):116-122
